# Proceedings of the Thirteenth International Society of Sports Nutrition (ISSN) Conference and Expo: Clearwater Beach, FL, USA. 9-11 June 2016

**DOI:** 10.1186/s12970-016-0144-9

**Published:** 2016-09-12

**Authors:** Lalitha Ramaswamy, Supriya Velraja, Guillermo Escalante, Phil Harvey, Michelle Alencar, Bryan Haddock, Phil Harvey, Guillermo Escalante, Michelle Alencar, Bryan Haddock, Krzysztof Durkalec-Michalski, Jan Jeszka, Bogna Zawieja, Tomasz Podgórski, Ana Paula Trussardi Fayh, Alexandre Hideki Okano, Amanda Maria de Jesus Ferreira, Ralf Jäger, Martin Purpura, Roger C. Harris, Molly M. Krause, Kiley A. Lavanger, Nina O. Allen, Allison E. Lieb, Katie A. Mullen, Joan M. Eckerson, Kiley A. Lavanger, Molly M. Krause, Nina O. Allen, Allison E. Lieb, Katie A. Mullen, Joan M. Eckerson, Elisa Morales, Jeffrey Forsse, Thomas Andre, Sarah McKinley, Paul Hwang, Grant Tinsley, Mike Spillane, Peter Grandjean, Darryn Willoughby, A. Jagim, G. Wright, J. Kisiolek, M. Meinking, J. Ochsenwald, M. Andre, M. T. Jones, J. M. Oliver, Victor Araújo Ferreira, Daniel Costa de Souza, Victor Oliveira Albuquerque dos Santos, Rodrigo Alberto Vieira Browne, Eduardo Caldas Costa, Ana Paula Trussardi Fayh, Suresh T. Mathews, Haley D. Bishop, Clara R. Bowen, Yishan Liang, Emily A. West, Rebecca R. Rogers, Mallory R. Marshall, John K. Petrella, A. Maleah Holland, Wesley C. Kephart, Petey W. Mumford, C. Brooks Mobley, Ryan P. Lowery, Jacob M. Wilson, Michael D. Roberts, Eric T. Trexler, Katie R. Hirsch, Bill I. Campbell, Meredith G. Mock, Abbie E. Smith-Ryan, Kate Zemek, Carol Johnston, C. Brooks Mobley, Petey W. Mumford, David D. Pascoe, Christopher M. Lockwood, Michael E. Miller, Michael D. Roberts, Gabriel J. Sanders, Willard Peveler, Brooke Warning, Corey A. Peacock, Wesley C. Kephart, Petey W. Mumford, Ryan P. Lowery, Michael D. Roberts, Jacob M. Wilson, David Sandler, Sara Perez Ojalvo, James Komorowski, Bill I. Campbell, Danielle Aguilar, Andres Vargas, Laurin Conlin, Amey Sanders, Paola Fink-Irizarry, Layne Norton, Ross Perry, Ryley McCallum, Matthew R. Wynn, Jack Lenton, Bill I. Campbell, Chris Gai, Seth Donelson, Shiva Best, Daniel Bove, Kaylee Couvillion, Jeff Dolan, Dante Xing, Kyshia Chernesky, Michael Pawela, Andres D. Toledo, Rachel Jimenez, M. Rabideau, A. Walker, J. Pellegrino, M. Hofacker, B. McFadden, S. Conway, C. Ordway, D. Sanders, R. Monaco, M. S. Fragala, S. M. Arent, Jason D. Stone, Andreas Kreutzer, Jonathan M. Oliver, Jacob Kisiolek, Andrew R. Jagim, M. Hofacker, A. Walker, J. Pellegrino, M. Rabideau, B. McFadden, S. Conway, D. Sanders, C. Ordway, R. Monaco, M. S. Fragala, S. M. Arent, Ozlem Tok, Joseph K. Pellegrino, Alan J. Walker, David J. Sanders, Bridget A. McFadden, Meaghan M. Rabideau, Sean P. Conway, Chris E. Ordway, Marissa Bello, Morgan L. Hofacker, Nick S. Mackowski, Anthony J. Poyssick, Eddie Capone, Robert M. Monaco, Maren S. Fragala, Shawn M. Arent, Petey W. Mumford, A. Maleah Holland, Wesley C. Kephart, Ryan P. Lowery, C. Brooks Mobley, Romil K. Patel, Annie Newton, Darren T. Beck, Michael D. Roberts, Jacob M. Wilson, Kaelin C. Young, Tobin Silver, Anya Ellerbroek, Richard Buehn, Leo Vargas, Armando Tamayo, Corey Peacock, Jose Antonio, Anya Ellerbroek, Tobin Silver, Richard Buehn, Leo Vargas, Armando Tamayo, Corey Peacock, Jose Antonio, Adam Pollock, Anya Ellerbroek, Tobin Silver, Corey Peacock, Jose Antonio, A. Kreutzer, P. Zavala, S. Fleming, M. Jones, J. M. Oliver, A. Jagim, Cody T. Haun, Petey W. Mumford, Parker N. Hyde, Ciaran M. Fairman, Wesley C. Kephart, Darren T. Beck, Jordan R. Moon, Michael D. Roberts, Kristina L. Kendall, Kaelin C. Young, Geoffrey M. Hudson, Tara Hannings, Kyle Sprow, Loretta DiPietro, Doug Kalman, Sara Perez Ojalvo, James Komorowski, P. Zavala, S. Fleming, M. Jones, J. Oliver, A. Jagim, Brian Wallace, Haley Bergstrom, Kelly Wallace, Matias Monsalves-Alvarez, Sebastian Oyharçabal, Victoria Espinoza, Trisha A. VanDusseldorp, Kurt A. Escobar, Kelly E. Johnson, Nathan Cole, Terence Moriarty, Matthew Stratton, Marvin R. Endito, Christine M. Mermier, Chad M. Kerksick, Matthew A. Romero, C. Brooks Mobley, Melissa Linden, Grace Margaret-Eleanor Meers, R. Scott Rector, Michael D. Roberts, Joshua L Gills, Hocheng Lu, Kimberly Parker, Chris Dobbins, Joshua N. Guillory, Braden Romer, David Szymanski, Jordan Glenn, Daniel E. Newmire, Eric Rivas, Sarah E. Deemer, Robert Wildman, Victor Ben-Ezra, C. Kerksick, B. Gieske, R. Stecker, C. Smith, K. Witherbee, Michael T. Lane, M. Travis Byrd, Zachary Bell, Emily Frith, Lauren M. C. Lane, Michael T. Lane, M. Travis Byrd, Zachary Bell, Emily Frith, Lauren M. C. Lane, Corey A. Peacock, Tobin A. Silver, Megan Colas, Mauricio Mena, Winter Rodriguez, Gabriel J. Sanders, Jose Antonio, Andrea Vansickle, Brittany DiFiore, Stephanie Stepp, Grant Slack, Bridget Smith, Kayla Ruffner, Ronald Mendel, Lonnie Lowery, Katie R. Hirsch, Meredith G. Mock, Malia M. N. Blue, Eric T. Trexler, Erica J. Roelofs, Abbie E. Smith-Ryan, Laurin Conlin, Danielle Aguilar, Bill I. Campbell, Layne Norton, Katie Coles, Eric T. Trexler, Nic Martinez, Jordan M. Joy, Roxanne M. Vogel, Thomas H. Hoover, K. Shane Broughton, R. Dalton, R. Sowinski, T. Grubic, P. B. Collins, A. Colletta, A. Reyes, B. Sanchez, M. Kozehchain, Y. P. Jung, C. Rasmussen, P. Murano, C. P. Earnest, M. Greenwood, R. B. Kreider, T. Grubic, R. Dalton, R. Sowinski, P. B. Collins, A. Colletta, A. Reyes, B. Sanchez, M. Kozehchain, Y. P. Jung, C. Rasmussen, P. Murano, C. P. Earnest, M. Greenwood, R. B. Kreider, R. Sowinski, R. Dalton, T. Grubic, P. B. Collins, A. Colletta, A. Reyes, B. Sanchez, M. Kozehchain, Y. P. Jung, C. Rasmussen, P. Murano, C. P. Earnest, M. Greenwood, R. B. Kreider, Krzysztof Durkalec-Michalski, Jan Jeszka, Tomasz Podgórski, C. Kerksick, B. Gieske, R. Stecker, C. Smith, K. Witherbee, Stacie Urbina, Emily Santos, Katelyn Villa, Alyssa Olivencia, Haley Bennett, Marissa Lara, Cliffa Foster, Colin Wilborn, Lem Taylor, Jason M Cholewa, Amy Hewins, Samantha Gallo, Ashley Micensky, Christian de Angelis, Christopher Carney, Bill Campbell, Laurin Conlin, Layne Norton, Fabricio Rossi, M. S. Koozehchian, P. B. Collins, R. Sowinski, T. Grubic, R. Dalton, A. O’Connor, S. Y. Shin, Y. Peter Jung, B. K. Sanchez, A. Coletta, M. Cho, A. Reyes, C. Rasmussen, C. P. Earnest, P. S. Murano, M. Greenwood, R. B. Kreider

**Affiliations:** 1 Department of Nutrition and Dietetics, PSG College of Arts and Science, Coimbatore, India; 2 Department of Clinical Nutrition, Sri Ramachandra University, Chennai, India; 3 Department of Kinesiology, California State University, San Bernardino, USA; 4 Max Muscle Sports Nutrition, Orange, CA USA; 5 University of Phoenix, San Diego Campus, CA USA; 6 Department of Kinesiology, California State University, Long Beach, CA USA; 7 Max Muscle Sports Nutrition, Orange, CA USA; 8 University of Phoenix, San Diego Campus, CA USA; 9 Department of Kinesiology, California State University, San Bernardino, CA USA; 10 Department of Kinesiology, California State University, Long Beach, CA USA; 11 Department of Hygiene and Human Nutrition, Poznan University of Life Sciences, Wojska Polskiego 31, 60-624 Poznan, Poland; 12 Polish Wrestling Federation, Żelazna 67/73, 00-871 Warsaw, Poland; 13 Department of Mathematical and Statistical Methods, Poznan University of Life Sciences, Wojska Polskiego 31, 60-624 Poznan, Poland; 14 Department of Biochemistry, University School of Physical Education in Poznan, Królowej Jadwigi 27/39, 61-871 Poznań, Poland; 15 Department of Nutrition, Federal University of Rio Grande do Norte, Natal, RN Brazil; 16 Increnovo LLC, 2138 E Lafayette Pl, Milwaukee, WI 53202 USA; 17 Junipa Ltd, Newmarket, Suffolk UK; 18 Department of Exercise Science and Pre-Health Professions, Creighton University, Omaha, NE USA; 19 Department of Exercise Science and Pre-Health Professions, Creighton University, Omaha, NE USA; 20 Department of Health, Human Performance, and Recreation, Baylor University, Waco, TX USA; 21 Exercise & Sport Science Department, University of Wisconsin – La Crosse, La Crosse, WI USA; 22 Division of Health and Human Performance, George Mason University, Fairfax, VA USA; 23 Kinesiology Department, Texas Christian University, Fort Worth, TX USA; 24 Department of Physical Education, Federal University of Rio Grande do Norte, Natal, RN Brazil; 25 Department of Nutrition, Federal University of Rio Grande do Norte, Natal, RN Brazil; 26 Department of Nutrition and Dietetics, Department of Kinesiology, Samford University, Birmingham, AL USA; 27 School of Kinesiology, Auburn University, Auburn, AL USA; 28 Department of Kinesiology, Augusta University, Augusta, GA USA; 29 Applied Science and Performance Institute, Tampa, FL USA; 30 Edward Via College of Osteopathic Medicine, Auburn Campus, Auburn, AL USA; 31 Human Movement Science Curriculum, University of North Carolina, Chapel Hill, NC USA; 32 Department of Exercise and Sport Science, University of North Carolina, Chapel Hill, NC USA; 33 Performance & Physique Enhancement Laboratory, University of South Florida, Tampa, FL USA; 34 Arizona State University, Phoenix, AZ USA; 35 School of Kinesiology, Auburn University, Auburn, AL USA; 36 Harrison School of Pharmacy, Auburn University, Auburn, AL USA; 37 Lockwood, LLC, Salt Lake City, Utah USA; 38 Northern Kentucky University, Highland Heights, KY USA; 39 Nova Southeastern University, Fort Lauderdale, FL USA; 40 School of Kinesiology, Auburn University, Auburn, AL USA; 41 Applied Science and Performance Institute, Tampa, FL USA; 42 StrengthPro Inc., Golden, CO USA; 43 Nutrition 21, LLC, Purchase, NY USA; 44 University of South Florida, Performance & Physique Enhancement Laboratory, Tampa, FL USA; 45 University of South Florida, Performance & Physique Enhancement Laboratory, Tampa, FL USA; 46 IFNH Center for Health & Human Performance, Rutgers University, New Brunswick, New Jersey USA; 47 Department of Intercollegiate Athletics, Rutgers University, New Brunswick, New Jersey USA; 48 Sports and Human Performance Diagnostics, Quest Diagnostics, New Brunswick, New Jersey USA; 49 Exercise & Sport Performance Laboratory, Department of Kinesiology, Texas Christian University, Fort Worth, TX USA; 50 Exercise & Sport Science Department, University of Wisconsin – La Crosse, La Crosse, WI 54603 USA; 51 IFNH Center for Health & Human Performance, Rutgers University, New Brunswick, New Jersey USA; 52 Dept. of Intercollegiate Athletics, Rutgers University, New Brunswick, New Jersey USA; 53 Sports and Human Performance Diagnostics, Quest Diagnostics, New Brunswick, New Jersey USA; 54 Center for Health and Human Performance, Rutgers University, New Brunswick, New Jersey USA; 55 Sport & Human Performance Diagnostics, Quest Diagnostics, New Brunswick, New Jersey USA; 56 Department of Intercollegiate Athletics, Rutgers University, New Brunswick, New Jersey USA; 57 School of Kinesiology, Auburn University, Auburn, AL USA; 58 Department of Kinesiology and Health Sciences, Augusta University, Augusta, GA USA; 59 Applied Science and Performance Institute, Tampa, FL USA; 60 Edward Via College of Osteopathic Medicine – Auburn Campus, Auburn, AL USA; 61 Nova Southeastern University, Exercise and Sport Science Lab, Davie, Florida USA; 62 Nova Southeastern University, Exercise and Sport Science Lab, Davie, Florida USA; 63 Nova Southeastern University, Exercise and Sport Science Lab, Davie, Florida USA; 64 Kokopelli’s Gym Inc., Casselberry, Florida USA; 65 Exercise & Sport Performance Laboratory, Kinesiology Department, Texas Christian University, Fort Worth, TX USA; 66 Exercise & Sport Science Department, University of Wisconsin – La Crosse, La Crosse, WI USA; 67 Division of Health and Human Performance, George Mason University, Fairfax, VA USA; 68 School of Kinesiology, Auburn University, Auburn, AL USA; 69 Department of Human Sciences, Ohio State University, Columbus, OH USA; 70 Edward Via College of Osteopathic Medicine – Auburn Campus, Auburn, AL USA; 71 School of Health sciences, American Public University System, Charles Town, WV USA; 72 Bodybuilding.com, Boise, ID USA; 73 The George Washington University, Washington, D.C., USA; 74 La Salle University, Philadelphia, PA USA; 75 Metavantage Sciences, Inc, Weston, FL USA; 76 Nutrition 21, LLC, Purchase, NY USA; 77 Exercise & Sport Science Department, University of Wisconsin – La Crosse, La Crosse, WI USA; 78 Division of Health and Human Performance, George Mason University, Fairfax, VA USA; 79 Exercise and Sport Performance Laboratory, Kinesiology Department, Texas Christian University, Fort Worth, TX USA; 80 Department of Kinesiology and Health Promotion, University of Kentucky, Lexington, KY 40506 USA; 81 Institute of Nutrition and Food Technology, University of Chile, Santiago, Chile; 82 Exercise Physiology Laboratory. Department of Biological Sciences. Faculty of Biological Sciences, Universidad Andres Bello, Santiago, Chile; 83 School of Kinesiology, Health Research Centre, Faculty of Medicine, Finis Terrae University, Santiago, Chile; 84 Center of Biomedical and Applied Research, Medicine School, Faculty of Medicinal Sciences, University of Santiago de Chile USACH, Santiago, Chile; 85 Department of Health, Exercise, and Sport Sciences, University of New Mexico, Albuquerque, NM USA; 86 Department of Sport, Recreation, and Exercise Sciences, Lindenwood University, St. Charles, MO USA; 87 School of Kinesiology, Auburn University, Auburn, AL USA; 88 Internal Medicine-Division of Gastroenterology and Hepatology/NEP, University of Missouri, Columbia, MO USA; 89 Louisiana Tech University, Ruston, LA USA; 90 High Point University, High Point, NC USA; 91 Omada Health, San Francisco, CA USA; 92 Exercise and Biochemistry Laboratory, Department of Kinesiology, Texas Woman’s University, Denton, TX USA; 93 Institute for Clinical and Translational Science & Department of Pediatrics, The University of California, Irvine, CA USA; 94 Dymatize Nutrition Sport Performance Institute, Dallas, TX USA; 95 School of Sport, Recreation and Exercise Sciences, Lindenwood University, St. Charles, MO USA; 96 Eastern Kentucky University, Richmond, KY USA; 97 University of Kentucky, Lexington, KY USA; 98 Eastern Kentucky University, Richmond, KY USA; 99 University of Kentucky, Lexington, KY USA; 100 Nova Southeastern University, Department of Health and Human Performance, Fort Lauderdale, FL USA; 101 Department of Kinesiology, Northern Kentucky University, Highland Heights, KY USA; 102 University of Mount Union, Alliance, OH USA; 103 Applied Physiology Laboratory, Department of Exercise and Sport Science, University of North Carolina, Chapel Hill, NC USA; 104 Nutrition, Health, and Human Performance Department, Meredith College, Raleigh, NC USA; 105 University of South Florida, Performance & Physique Enhancement Laboratory, Tampa, FL USA; 106 Human Movement Science Curriculum, University of North Carolina, Chapel Hill, NC USA; 107 Department of Nutrition and Food Sciences, Texas Woman’s University, Denton, TX USA; 108 InnovaSolutions LLC, Denton, TX USA; 109 Exercise & Sport Nutrition Lab, Texas A&M University, College Station, TX USA; 110 Institute for Obesity and Program Evaluation, Texas A&M University, College Station, TX USA; 111 Nutrabolt, Bryan, TX USA; 112 Exercise & Sport Nutrition Lab, Texas A&M University, College Station, TX USA; 113 Institute for Obesity and Program Evaluation, Texas A&M University, College Station, TX USA; 114 Nutrabolt, Bryan, TX USA; 115 Exercise & Sport Nutrition Lab, Texas A&M University, College Station, TX USA; 116 Institute for Obesity and Program Evaluation, Texas A&M University, College Station, TX USA; 117 Nutrabolt, Bryan, TX USA; 118 Department of Hygiene and Human Nutrition, Poznan University of Life Sciences, Wojska Polskiego 31, 60-624 Poznan, Poland; 119 Polish Wrestling Federation, Żelazna 67/73, 00-871 Warsaw, Poland; 120 Department of Biochemistry, University School of Physical Education in Poznan, Królowej Jadwigi 27/39, 61-871 Poznań, Poland; 121 School of Sport, Recreation and Exercise Sciences, Lindenwood University, St. Charles, MO USA; 122 Department of Exercise & Sports Science, Human Performance Lab, University of Mary Hardin-Baylor, Belton, TX USA; 123 Department of Kinesiology, Coastal Carolina University, Conway, SC 29528 USA; 124 University of South Florida, Performance & Physique Enhancement Laboratory, Tampa, FL USA; 125 Biolayne, LLC, Lutz, FL USA; 126 Institute of Bioscience, Department of Physical Education, University Estadual Paulista, Rio Claro, São Paulo Brazil; 127 Exercise & Sport Nutrition Lab, Texas A&M University, College Station, TX USA; 128 Institute for Obesity Research & Program Evaluation, Texas A&M University, College Station, TX USA; 129 Nutrabolt, Bryan, TX USA

## Abstract

P1 Impact of antioxidant-enriched nutrient bar supplementation on the serum antioxidant markers and physical fitness components of track and field athletes

Lalitha Ramaswamy, Supriya Velraja

P2 The effects of phosphatidic acid supplementation on fitness levels in resistance trained women

Guillermo Escalante, Phil Harvey, Michelle Alencar, Bryan Haddock

P3 The effects of phosphatidic acid supplementation on cardiovascular risk factors in resistance trained men

Phil Harvey, Guillermo Escalante, Michelle Alencar, Bryan Haddock

P4 The efficacy of sodium bicarbonate supplementation on physical capacity and selected biochemical markers in elite wrestlers

Krzysztof Durkalec-Michalski, Jan Jeszka, Bogna Zawieja, Tomasz Podgórski

P5 Effects of different nutritional strategies in hydration and physical performance in healthy well-trained males

Ana Paula Trussardi Fayh, Alexandre Hideki Okano, Amanda Maria de Jesus Ferreira

P6 Reduction of plasma creatine concentrations as an indicator of improved bioavailability

Ralf Jäger, Martin Purpura, Roger C Harris

P7 Effect of three different breakfast meals on energy intake and nutritional status in college-age women

Molly M. Krause, Kiley A. Lavanger, Nina O. Allen, Allison E. Lieb, Katie A. Mullen, Joan M. Eckerson

P8 Accuracy of the ASA24® Dietary Recall system for assessing actual dietary intake in normal weight college-age women.

Kiley A. Lavanger, Molly M. Krause, Nina O. Allen, Allison E. Lieb, Katie A. Mullen, Joan M. Eckerson

P9 β-aminoisobutyric acid does not regulate exercise induced UCP-3 expression in skeletal muscle

Elisa Morales, Jeffrey Forsse, Thomas Andre, Sarah McKinley, Paul Hwang, Grant Tinsley, Mike Spillane, Peter Grandjean, Darryn Willoughby

P10 The ability of collegiate football athletes to adhere to sport-specific nutritional recommendations

A. Jagim, G. Wright, J. Kisiolek, M. Meinking, J. Ochsenwald, M. Andre, M.T. Jones, J. M. Oliver

P11 A single session of low-volume high intensity interval exercise improves appetite regulation in overweight men

Victor Araújo Ferreira, Daniel Costa de Souza, Victor Oliveira Albuquerque dos Santos, Rodrigo Alberto Vieira Browne, Eduardo Caldas Costa, Ana Paula Trussardi Fayh

P12 Acute effects of oral peppermint oil ingestion on exercise performance in moderately-active college students

Suresh T. Mathews, Haley D. Bishop, Clara R. Bowen, Yishan Liang, Emily A. West, Rebecca R. Rogers, Mallory R. Marshall, John K. Petrella

P13 Associations in body fat and liver triglyceride content with serum health markers in sedentary and exercised rats fed a ketogenic diet, Western diet or standard chow over a 6-week period

A. Maleah Holland, Wesley C. Kephart, Petey W. Mumford, C. Brooks Mobley, Ryan P. Lowery, Jacob M. Wilson, Michael D. Roberts

P14 Physiological changes following competition in male and female physique athletes: A pilot study

Eric T. Trexler, Katie R. Hirsch, Bill I. Campbell, Meredith G. Mock, Abbie E. Smith-Ryan

P15 Relationship between cognition and hydration status in college students at a large Southwestern university

Kate Zemek, Carol Johnston

P16 Whey protein-derived exosomes increase protein synthesis in C2C12 myotubes

C. Brooks Mobley, Petey W. Mumford, David D. Pascoe, Christopher M. Lockwood, Michael E. Miller, Michael D. Roberts

P17 The effect of three different energy drinks on 1.5-mile running performance, oxygen consumption, and perceived exertion

Gabriel J. Sanders, Willard Peveler, Brooke Warning, Corey A. Peacock

P18 The Ketogenic diet improves rotarod performance in young and older rats

Wesley C. Kephart, Petey W. Mumford, Ryan P. Lowery, Michael D. Roberts, Jacob M. Wilson

P19 Absorption of bonded arginine silicate compared to individual arginine and silicon components

David Sandler, Sara Perez Ojalvo, James Komorowski

P20 Effects of a high (2.4 g/kg) vs. low/moderate (1.2 g/kg) protein intake on body composition in aspiring female physique athletes engaging in an 8-week resistance training program

Bill I. Campbell, Danielle Aguilar, Andres Vargas, Laurin Conlin, Amey Sanders, Paola Fink-Irizarry, Layne Norton, Ross Perry, Ryley McCallum, Matthew R. Wynn, Jack Lenton

P21 Effects of a high (2.4 g/kg) vs. low/moderate (1.2 g/kg) protein intake on maximal strength in aspiring female physique athletes engaging in an 8-week resistance training program

Bill I. Campbell, Chris Gai, Seth Donelson, Shiva Best, Daniel Bove, Kaylee Couvillion, Jeff Dolan, Dante Xing, Kyshia Chernesky, Michael Pawela, Andres D. Toledo, Rachel Jimenez

P22 Monitoring of female collegiate athletes over a competitive season reveals changes in nutritional biomarkers

M. Rabideau, A. Walker, J. Pellegrino, M. Hofacker, B. McFadden, S. Conway, C. Ordway, D. Sanders, R. Monaco, M. S. Fragala, S. M. Arent

P23 Comparison of prediction equations to indirect calorimetry in men and women athletes

Jason D. Stone, Andreas Kreutzer, Jonathan M. Oliver, Jacob Kisiolek, Andrew R. Jagim

P24 Regional variations in sweat-based electrolyte loss and changes in plasma electrolyte content in Division I female athletes over the course of a competitive season

M. Hofacker, A. Walker, J. Pellegrino, M. Rabideau, B. McFadden, S. Conway, D. Sanders, C. Ordway, R. Monaco, M. S. Fragala, S. M. Arent

P25 In-season changes in plasma amino acid levels in Division I NCAA female athletes

Ozlem Tok, Joseph K. Pellegrino, Alan J. Walker, David J. Sanders, Bridget A. McFadden, Meaghan M. Rabideau, Sean P. Conway, Chris E. Ordway, Marissa Bello, Morgan L. Hofacker, Nick S. Mackowski, Anthony J. Poyssick, Eddie Capone, Robert M. Monaco, Maren S. Fragala, Shawn M. Arent

P26 Effects of a ketogenic diet with exercise on serum markers of bone metabolism, IGF-1 and femoral bone mass in rats

Petey W. Mumford, A. Maleah Holland, Wesley C. Kephart, Ryan P. Lowery, C. Brooks Mobley, Romil K. Patel, Annie Newton, Darren T. Beck, Michael D. Roberts, Jacob M. Wilson, Kaelin C. Young

P27 Casein supplementation in trained men and women: morning versus evening

Tobin Silver, Anya Ellerbroek, Richard Buehn, Leo Vargas, Armando Tamayo, Corey Peacock, Jose Antonio

P28 A high protein diet has no harmful effects: a one-year crossover study in resistance-trained males

Anya Ellerbroek, Tobin Silver, Richard Buehn, Leo Vargas, Armando Tamayo, Corey Peacock, Jose Antonio

P29 SUP (Stand-up Paddling) athletes: nutritional intake and body composition

Adam Pollock, Anya Ellerbroek, Tobin Silver, Corey Peacock, Jose Antonio

P30 The effects of 8 weeks of colostrum and bio-active peptide supplementation on body composition in recreational male weight lifters

A. Kreutzer, P. Zavala, S. Fleming, M. Jones, J. M. Oliver, A. Jagim

P31 Effects of a Popular Women’s Thermogenic Supplement During an Energy-Restricted High Protein Diet on Changes in Body Composition and Clinical Safety Markers

Cody T. Haun, Petey W. Mumford, Parker N. Hyde, Ciaran M. Fairman, Wesley C. Kephart, Darren T. Beck, Jordan R. Moon, Michael D. Roberts, Kristina L. Kendall, Kaelin C. Young

P32 Three days of caffeine consumption following caffeine withdrawal yields small strength increase in knee flexors

Geoffrey M Hudson, Tara Hannings, Kyle Sprow, Loretta DiPietro

P33 Comparison of cellular nitric oxide production from various sports nutrition ingredients

Doug Kalman, Sara Perez Ojalvo, James Komorowski

P34 The effects of 8 weeks of bio-active peptide supplementation on training adaptations in recreational male weight lifters

P. Zavala, S. Fleming, M. Jones, J. Oliver, A. Jagim

P35 Effects of MusclePharm Assault Black^TM^ on lower extremity spinal excitability and postactivation potentiation: A pilot study

Brian Wallace, Haley Bergstrom, Kelly Wallace

P36 Effects of four weeks of Ketogenic Diet alone and combined with High intensity Interval Training or Continuous-Moderate intensity on body composition, lipid profile and physical performance on healthy males

Matias Monsalves-Alvarez, Sebastian Oyharçabal, Victoria Espinoza

P37 Effect of branched-chain amino acid supplementation on creatine kinase, muscular performance, and perceived muscle soreness following acute eccentric exercise

Trisha A. VanDusseldorp, Kurt A. Escobar, Kelly E. Johnson, Nathan Cole, Terence Moriarty, Matthew Stratton, Marvin R. Endito, Christine M. Mermier, Chad M. Kerksick

P38 Effects of endurance training on markers of ribosome biogenesis in rodents fed a high fat diet

Matthew A. Romero, C. Brooks Mobley, Melissa Linden, Grace Margaret-Eleanor Meers, R. Scott Rector, Michael D. Roberts

P39 The effects of acute citrulline-malate on lower-body isokinetic performance in recreationally active individuals

Joshua L Gills, Hocheng Lu, Kimberly Parker, Chris Dobbins, Joshua N Guillory, Braden Romer, David Szymanski, Jordan Glenn

P40 The effect pre-ingested L-isoleucine and L-leucine on blood glucose responses and glycemic hormones in healthy inactive adults: Preliminary data.

Daniel E. Newmire, Eric Rivas, Sarah E. Deemer, Robert Wildman, Victor Ben-Ezra

P41 Does protein and source impact substrate oxidation and energy expenditure during and after moderate intensity treadmill exercise?

C Kerksick, B Gieske, R Stecker, C Smith, K Witherbee

P42 Effects of a pre-workout supplement on peak power and power maintenance during lower and upper body testing

Michael T. Lane, M. Travis Byrd, Zachary Bell, Emily Frith, Lauren M.C. Lane

P43 Effects of a pre-workout supplement on peak power production during lower and upper body testing in college-age females

Michael T. Lane, M. Travis Byrd, Zachary Bell, Emily Frith, Lauren M.C. Lane

P44 A comparison of whey versus casein protein supplementation on resting metabolic rate and body composition: a pilot study

Corey A. Peacock, Tobin A. Silver, Megan Colas, Mauricio Mena, Winter Rodriguez, Gabriel J. Sanders, Jose Antonio

P45 A novel mixed-tocotrienol intervention enhances recovery after eccentric exercise: preliminary findings

Andrea Vansickle, Brittany DiFiore, Stephanie Stepp, Grant Slack, Bridget Smith, Kayla Ruffner, Ronald Mendel, Lonnie Lowery

P46 The effects of post-exercise ingestion of a high molecular weight glucose on cycle performance in female cyclists

Katie R. Hirsch, Meredith G. Mock, Malia M.N. Blue, Eric T. Trexler, Erica J. Roelofs, Abbie E. Smith-Ryan

P47 Inclusive vs. exclusive dieting and the effects on body composition in resistance trained individuals

Laurin Conlin, Danielle Aguilar, Bill I. Campbell, Layne Norton, Katie Coles, Eric T. Trexler, Nic Martinez

P48 A whey protein hydrolysate may positively augment resting metabolism compared to intact whey protein

Jordan M. Joy, Roxanne M. Vogel, Thomas H. Hoover, K. Shane Broughton

P49 Seven days of high and low dose creatine nitrate supplementation I: hepatorenal, glucose and muscle enzyme function

R Dalton, R Sowinski, T Grubic, PB Collins, A Colletta, A Reyes, B Sanchez, M Kozehchain, YP Jung, C Rasmussen, P Murano, CP Earnest, M Greenwood, RB Kreider

P50 Seven days of high and low dose creatine nitrate supplementation II: performance

T Grubic, R Dalton, R Sowinski, PB Collins, A Colletta, A Reyes, B Sanchez, M Kozehchain, YP Jung, C Rasmussen, P Murano, CP Earnest, M Greenwood, RB Kreider

P51 Seven days of high and low dose creatine nitrate supplementation III: hemodynamics

R Sowinski, R Dalton, T Grubic, PB Collins, A Colletta, A Reyes, B Sanchez, M Kozehchain, YP Jung, C Rasmussen, P Murano, CP Earnest, M Greenwood, RB Kreider

P52 The efficacy of a β-hydroxy-β-methylbutyrate supplementation on physical capacity, body composition and biochemical markers in highly-trained combat sports athletes

Krzysztof Durkalec-Michalski, Jan Jeszka, Tomasz Podgórski

P53 Does protein and source impact substrate oxidation and energy expenditure during and after moderate intensity treadmill exercise?

C Kerksick, B Gieske, R Stecker, C Smith, K Witherbee

P54 Effects of 30 days of Cleanse™ supplementation on measure of body composition, waist circumference, and markers of gastrointestinal distress in females

Stacie Urbina, Emily Santos, Katelyn Villa, Alyssa Olivencia, Haley Bennett, Marissa Lara, Cliffa Foster, Colin Wilborn, Lem Taylor

P55 The effects of moderate- versus high-load training on body composition, muscle growth, and performance in college aged females

Jason M Cholewa, Amy Hewins, Samantha Gallo, Ashley Micensky, Christian De Angelis, Christopher Carney, Bill Campbell, Laurin Conlin, Layne Norton, Fabricio Rossi

P56 Effect of a multi-ingredient preworkout supplement on cognitive function and perceptions of readiness to perform

MS Koozehchian, PB Collins, R Sowinski, T Grubic, R Dalton, A O’Connor, SY Shin, Y Peter Jung, BK Sanchez, A Coletta, M Cho, A Reyes, C Rasmussen, CP Earnest, PS Murano, M Greenwood, RB Kreider

## P1 Impact of antioxidant-enriched nutrient bar supplementation on the serum antioxidant markers and physical fitness components of track and field athletes

### Lalitha Ramaswamy^1^, Supriya Velraja^2^

#### ^1^Department of Nutrition and Dietetics, PSG College of Arts and Science, Coimbatore, India; ^2^Department of Clinical Nutrition, Sri Ramachandra University, Chennai, India

##### **Correspondence:** Lalitha Ramaswamy (lalitharam58@gmail.com) – Department of Nutrition and Dietetics, PSG College of Arts and Science, Coimbatore, India

**Background**

Antioxidant supplementation may provide protection against negative health consequences of oxygen-free radicals caused by aerobic and re-sustained exercise. The aim is to find out the efficacy of antioxidant rich nutrient bar supplementation on the antioxidant status and physical fitness components of athletes.

**Method**

Forty track and field athletes were selected using convenience sampling technique. The Human Research Ethics Committee of PSG College of Arts and Science, Coimbatore approved the study. Voluntary participation of the subjects was emphasized and a written consent was obtained from them in order to be included in the study. An interview schedule was formulated to collect general information such as name, age, gender, and academic qualifications, as well as information pertaining to the types of sporting activity, duration of the activity per day, number of years involved in the respective sports and level of participation (district/state/national). Nutrient bars each weighing 50gm were prepared with rolled oats, pumpkin seeds, dehydrated carrots, flax seeds, peanuts, almonds, honey and date syrup as ingredients. Each athlete was provided with two bars containing 110 mg of antioxidant. The prepared bars were analyzed for their total anti- oxidant content using DPPH method. The experimental group (n = 20) was supplemented with the formulated nutrient bars every day for a period of 3 months, and the control group (n = 20) with a placebo. Bio-chemical parameters namely GSH, GSH-px, SOD, vitamin C, serum LPO and physical fitness tests such as 12 minutes test, speed test, step test, push-ups test, vertical jump test and hexagon agility test were assessed at baseline and after 90 days. Statistical analysis was performed using SPSS (version 15).

**Results**

The mean age was 18 ± 3.2 yrs. Sixty percent of the selected athletes were males and 40 % females. The main source of nutrition information was from coaches (56 %) and magazines (30 %). The athletes of the experimental group, when compared with the control group, showed a significant increase in serum levels from 37.42 ± 12.01 units/min/ml to 42.08 ± 13.16 units/min/ml of SOD (p = 0.000). LPO increased from 2.89 ± 0.82 μg/ml to 3.80 ± 1.37 μg/ml (p = 0.005), GSH increased from 209.76 ± 8.17 μg/ml to 244.58 ± 33.36 μg/ml (p = 0.000). The mean levels of vitamin C and GSH-px decreased minimally in the experimental group but significantly in the control group. Significant improvement in all six physical fitness tests namely 12 minutes test (from 1.44 ± 0.142 to 1.56 ± 0.134) , speed test (from 6.5940 ± 0.258 to 6.7435 ± 0.25) step test (from 68.65 ± 5.51 to 78.35 ± 4.004) push-ups (from 22.25 ± 3.97 to 29.70 ± 3.40), vertical jumps test (from 60.00 ± 5.620 to 63.75 ± 6.043) and hexagon agility tests (from 12.255 ± 0.42 to 12.400 ± 0.51) were observed in the experimental group). However in the control group, the improvements were significant only in the 12 minutes test, step test and speed test.

**Conclusion**

Supplementation of athletes with antioxidant-rich nutrient bars clearly improved their bio-markers and physical fitness. Future research may explore the effect of other nutritional antioxidants that could benefit the athletes.

## P2 The effects of phosphatidic acid supplementation on fitness levels in resistance trained women

### Guillermo Escalante^1^, Phil Harvey^2,3^, Michelle Alencar^4^, Bryan Haddock^1^

#### ^1^Department of Kinesiology, California State University, San Bernardino, USA; ^2^Max Muscle Sports Nutrition, Orange, CA, USA; ^3^University of Phoenix, San Diego Campus, CA, USA; ^4^Department of Kinesiology, California State University, Long Beach, CA, USA

##### **Correspondence:** Guillermo Escalante (gescalante23@yahoo.com) – Department of Kinesiology, California State University, San Bernardino, USA

**Background**

Recent research findings have demonstrated a link between phosphatidic acid (PA) and muscle protein synthesis via signaling of a protein kinase known as the mechanistic target of rapamycin (mTOR). No current studies have investigated the effects of PA supplementation on fitness levels in resistance trained women. This study was a double-blind placebo controlled study investigating the effects of a compound containing phosphatidic acid (PA) in enhancing resistance-training induced changes in body composition, strength, and other sports performance markers. MaxxTOR® (MT), which is a supplement that contains 750 mg of PA as the main active ingredient, is the supplement that was used for this investigation.

**Methods**

Initially, ten female subjects were randomly placed in the MaxxTOR® (MT) group and nine female subjects were randomly placed in the placebo (PLA) group, but five subjects dropped from the PLA group dropped out of the study due to time constraints. A total of fourteen subjects (n = 14, age = 24.5 +/- 3.9 yrs, height = 164.63 +/- 2.3 cm, weight = 73.2 +/- 12.9 kg) completed the study. All subjects underwent pre and post exercise testing for 1-RM leg press, 1-RM bench press, body composition/thigh muscle mass via Dual Energy X-ray Absorptiometry (DXA), push-ups to failure, Wingate test, vertical jump, and pro-agility shuttle. All subjects were provided with a customized iso-caloric diet with the same macronutrient breakdown and underwent the same supervised periodized workout regimen. The MT group received the active ingredient for 8 weeks and the PLA group received an identical looking supplement with no active ingredients for the same period.

**Results**

Separate two-way mixed factorial repeated measures Analysis of Variance (time [Pre, Post] x group [MT and PLA] were used to investigate body composition changes (lean body mass, fat mass, thigh muscle mass) strength changes (1 RM Bench and Leg Press), and other performance changes (max push-ups, Wingate power, vertical jump, and agility). When significant main effects were found, a Tukey post-hoc was conducted to determine where the differences occurred. The MT group increased lean body mass to a greater extent (pre: 40.8 ± 4.8 kg; post: 42.5 ± 4.8 kg) when compared to the PLA group (pre: 43.2 ± 2.1 kg; post: 43.4 ± 2.6 kg) (p ≤ 0.001). The MT group also increased 1-RM Leg Press strength significantly greater (pre: 154.5.6 ± 35.7 kg; post: 219.3 ± 41.8 kg) than the PLA group (pre: 186.4 ± 21.3 kg; post: 219.9 ± 35.9 kg) (p ≤ 0.002). Similarly, the MT group increased 1-RM Bench Press significantly greater (pre: 34.1 ± 4.4 kg; post: 44.5 ± 5.7 kg) than the PLA group (pre: 36.4 ± 3.2 kg; post: 40.3 ± 4.7 kg). No significant changes were noted in vertical jump, Wingate power test, maximum push-ups, estimated thigh muscle mass, or fat mass.

**Conclusion**

These results suggest MT can help improve upper body strength, lower body strength, and lean body mass as compared to PLA over an 8-week intervention in resistance trained women.

**Acknowledgements**

The authors would like to acknowledge Max Muscle Sports Nutrition (Orange, CA) and Chemi Nutra (Austin, TX) for their financial support in this investigation.

## P3 The effects of phosphatidic acid supplementation on cardiovascular risk factors in resistance trained men

### Phil Harvey^1,2^, Guillermo Escalante ^3^, Michelle Alencar^4^, Bryan Haddock^3^

#### ^1^Max Muscle Sports Nutrition, Orange, CA, USA; ^2^University of Phoenix, San Diego Campus, CA, USA; ^3^Department of Kinesiology, California State University, San Bernardino, CA, USA; ^4^Department of Kinesiology, California State University, Long Beach, CA, USA

##### **Correspondence:** Guillermo Escalante (gescalante23@yahoo.com) – Department of Kinesiology, California State University, San Bernardino, CA, USA

**Background**

Phosphatidic Acid (PA) is a compound formed by two fatty acids and a phosphate group that are covalently bonded to a glycerol molecule through ester linkages. PA is a precursor for the production of other lipids, it can act as a signaling lipid, and it is a major component of cell membranes. Recent research findings have demonstrated a link between PA and muscle protein synthesis through signaling of a protein kinase known as the mechanistic target of rapamycin (mTOR). Several studies have found PA to be effective at increasing strength and lean body mass in resistance trained subjects, but its safety has not been investigated. This study was a double-blind placebo controlled study investigating the effects of a compound containing phosphatidic acid (PA) on blood pressure, cholesterol, and triglycerides in resistance trained men. MaxxTOR® (MT), which is a supplement that contains 750 mg of PA as the main active ingredient, is the supplement that was used for this investigation.

**Methods**

Eighteen healthy strength-trained males were randomly assigned to a group that either consumed MT (n = 8, 22.0 +/- 2.5 yrs; 175.8 +/- 11.5 cm; 80.3 +/- 15.1 kg) or a placebo (PLA) (n = 10, 25.6 +/- 4.2 yrs; 174.8 +/- 9.0 cm; 88.6 +/- 16.6 kg) as part of a double-blind, placebo controlled pre/post experimental design. All subjects provided a fasted venipuncture blood sample at the university health center within 5 days prior to and five days after the exercise/supplementation protocol; furthermore, blood pressure was measured at these times prior to the blood draw. The blood was used to test for changes in cholesterol and triglycerides after all subjects closely followed a customized iso-caloric diet with the same macronutrient breakdown and underwent the same supervised periodized workout regimen for 8 weeks. The MT group received the active ingredient for 8 weeks and the PLA group received an identical looking supplement with no active ingredients for the same period.

**Results**

Separate two-way mixed factorial repeated measures Analysis of Variance (time [Pre, Post] x group [MT and PLA] were used to investigate changes in blood pressure, cholesterol, and triglycerides. No significant main effects were found in any of the variables. The systolic blood pressure in the MT group (pre: 122.5 +/- 9.4 mmHg; post 121.0 +/- 9.3 mmHg) and PLA group (pre: 121.8 +/- 6.2 mmHg; post 121.2 +/- 6.5 mmHg) (p = 0.135) did not change pre to post. Similarly, diastolic blood pressure in the MT group (pre: 65.5 +/- 2.6 mmHg; post 65.5 +/- 4.0 mmHg) and PLA group (pre: 70.0 +/- 6.3 mmHg; post 68.6 +/- 5.5 mmHg) (p = 0.407) did not change pre to post. Additionally, total cholesterol in the MT group (pre: 127.8 +/- 29.0 mg/dL; post 122.6 +/- 28.2 mg/dL) and the PLA group (pre: 122.7 +/- 25.3 mg/dL; post 128.8 +/- 25.7 mg/dL) (p = 0.926) did not change pre to post. Finally, triglycerides in the MT group (pre: 124.2 +/- 75.1 mg/dL; post 91.1 +/- 44.0 mg/dL) and the PLA group (pre: 139.2 +/- 72.6 mg/dL; post 174.8 +/- 127.9 mg/dL) (p = 0.960) did not change pre to post.

**Conclusion**

These results suggest MT supplementation does not impact blood pressure, cholesterol, or triglycerides after an 8-week intervention in resistance trained men.

**Acknowledgements**

The authors would like to acknowledge Max Muscle Sports Nutrition (Orange, CA) and Chemi Nutra (Austin, TX) for their financial support in this investigation.

## P4 The efficacy of sodium bicarbonate supplementation on physical capacity and selected biochemical markers in elite wrestlers

### Krzysztof Durkalec-Michalski^1,2^, Jan Jeszka^1^, Bogna Zawieja^3^, Tomasz Podgórski^4^

#### ^1^Department of Hygiene and Human Nutrition, Poznan University of Life Sciences, Wojska Polskiego 31, 60-624 Poznan, Poland; ^2^Polish Wrestling Federation, Żelazna 67/73, 00-871 Warsaw, Poland; ^3^Department of Mathematical and Statistical Methods, Poznan University of Life Sciences, Wojska Polskiego 31, 60-624 Poznan, Poland; ^4^Department of Biochemistry, University School of Physical Education in Poznan, Królowej Jadwigi 27/39, 61-871 Poznań, Poland

##### **Correspondence:** Krzysztof Durkalec-Michalski (durkmich@up.poznan.pl) – Polish Wrestling Federation, Żelazna 67/73, 00-871 Warsaw, Poland

**Background**

Increased the buffer capacity and reduction of muscle acidification could significantly affect athletes’ ability to exercise in combat sports. It seems that the above advantages can be achieved by supplementation of sodium bicarbonate (SB). Drawn attention the fact that the effect of SB uptake has rarely been verified on targeted exercises performance in combat sports. With this in mind the aim of this study was to evaluate the effect of sodium bicarbonate (SB) supplementation on the physical capacity of trained wrestlers.

**Methods**

The study involved 50 highly-trained wrestlers. After randomization procedure 29 athletes during 10 days were supplemented SB – Alkala T (SANUM), according to author's progressive cycle of the doses increase - from 0.025 g · kg^-1^ (days: 1-3) to 0.1 g · kg^-1^ SB (days: 8-10). The other 21 wrestlers received placebo (PLA: maltodextrin). The effectiveness of supplementation was evaluated on the basis of the anaerobic capacity evaluation after two classical Wingate tests (Monark 894E), combined with the analysis of biochemical indicators: lactate (La), pyruvate (Pa), creatine kinase (CK), between which performed a wrestler's special endurance test - projective test with dummy. A normal distribution of variables was tested using the Shapiro-Wilk test. The two way ANOVA for repeated measures for parametric outcomes, and the Scheirer-Ray-Hare extension of the Kruskal-Wallis test for non-normally distributed variables were applied. Moreover, in each group the significance of differences between the baseline (Pre) and the post-intervention (Post) parameters were calculated with the dependent samples t-tests (normally distributed variables) or Wilcoxon-signed rank tests (non-normally distributed variables).

**Results**

Significant differences in the SB group in comparison to PLA group in time at peak power - Tpp (SB: 2.4 ± 1.1 s vs. PLA: 3.3 ± 1.9 s, *P* = 0.02), as well as post exercise La (SB: 13.7 ± 2.8 mmol · L^-1^ vs. PLA: 7.7 ± 1.6 mmol · L^-1^, *P* < 0.0001) and Pa (SB: 0.48 ± 0.09 mmol · L^-1^ vs. PLA: 0.35 ± 0.06 mmol · L^-1^, *P* < 0.0001) concentrations were observed. In comparison to baseline after SB supply an increase in the peak power (~ +1.0 W · kg^-1^, *P* < 0.0001), average power (~ +0.19 W · kg^-1^, *P* = 0.012) power, and La (~ +6.0 mmol · L^-1^, *P* < 0.0001) and Pa (~ +0.15 mmol · L^-1^, *P* < 0.0001) concentrations as well as reducing Tpp (~ -0.75 s, *P* = 0.015) were recorded. In turn, after PLA (compared to baseline in this group) only peak power increased (~ +0.9 W · kg^-1^, *P* = 0.012). There were no significant changes in the results of the dummy projective test and CK activity.

**Conclusion**

The results of these studies indicate that SB supplementation significantly increased indices of anaerobic adaptation and tolerance on muscle acidification, but does not support the wrestler's special projective endurance.

**Acknowledgments**

The authors wish to thank the coaches and athletes for their help and participation in the research project. We gratefully acknowledge financial support for this work provided by the statutory funds of Department of Hygiene and Human Nutrition.

## P5 Effects of different nutritional strategies in hydration and physical performance in healthy well-trained males

### Ana Paula Trussardi Fayh, Alexandre Hideki Okano, Amanda Maria de Jesus Ferreira

#### Department of Nutrition, Federal University of Rio Grande do Norte, Natal, RN, Brazil

##### **Correspondence:** Ana Paula Trussardi Fayh (apfayh@yahoo.com.br) – Department of Nutrition, Federal University of Rio Grande do Norte, Natal, RN, Brazil

**Background**

Fatigue and dehydration are common conditions for high intensity sports. However, there is a gap in the literature about the most appropriate nutritional strategies for events of less than one hour. Thus, this study aimed to evaluate the effect of three different nutritional strategies on hydration variables and physical performance on cycling time trial.

**Methods**

Eleven well-trained males (30.4 ± 6.2 years old, BMI 24.7 ± 2.2 kg/m^2^, BF 11.2 ± 3.4 %) completed 30 km time trial test in cycle ergometer under the influence of three randomized cross-over interventions: CMR = carbohydrate mouth rinse; WWL = water with electrolyte intake according to weight loss; WAL = water intake "Ad Libitum". Variables as heart rate (HR), power (W) and perception of effort (PE) were evaluated during the test. Before and after exercise were measured body weight and urine samples for evaluated weight loss (WL) and dehydration. Statistical analyses were performed utilizing General Estimation Equation test employing a probability level of ≤ 0.05.

**Results**

The performance of the athletes with the time trial results did not differ between the interventions (54.5 ± 2.9 min, 53.6 ± 3.9 min and 54.5 ± 2.5 min in CMR, WWL and WAL, respectively, p = 0.13). The WL in WWL intervention was lower than in other interventions (p <0.05), the group with the highest intake of liquids (p <0.01). Lower urinary pH values were found in the CMR technique, at the end time test (p <0.05), but urinary density was not different between tests.

**Conclusion**

The results of this study further support the use of different nutritional strategies with different amount of fluids during high/moderate intensity exercise, because the hydration status did not influence the performance of athletes during the time trial.

## P6 Reduction of plasma creatine concentrations as an indicator of improved bioavailability

### Ralf Jäger^1^, Martin Purpura^1^, Roger C Harris^2^

#### ^1^Increnovo LLC, 2138 E Lafayette Pl, Milwaukee, WI 53202, USA; ^2^Junipa Ltd, Newmarket, Suffolk, UK

##### **Correspondence:** Ralf Jäger (ralf.jaeger@increnovo.com) – Increnovo LLC, 2138 E Lafayette Pl, Milwaukee, WI 53202, USA

**Background**

The retention of dietary creatine is a two-step process; firstly absorption into blood and secondly uptake into the target tissue, principally muscle. Increases in blood plasma creatine concentration are often interpreted as indicating improved bioavailability. However, an increase in the concentration of creatine in plasma could equally be the result of a lower uptake into the target tissue signifying in fact a decrease in overall bioavailability. An increase in circulating insulin in response to glucose administration has previously been shown to increase creatine retention in the muscle. The aim of this study was to compare the effects of ingesting tricreatine citrate (5 g, TCrC) with or without the co-administration of 75 g of glucose and 200 mg of alpha-lipoic acid (TCrC + Glu + ALA, CELL-TECH RTD, Iovate Health Sciences, Oakville, ON, Canada) on creatine concentrations in plasma and urinary creatine elimination during 8 hours following each of the treatments.

**Methods**

Three male and three female healthy subjects (35.5+/-14.5 yrs, 172.5+/-12.2 cm, 75.3+/-9.0 kg) participated in the study. No subject in this trial was a vegetarian with all subjects reportedly consuming meat in their daily diet. The study used a cross-over design. Each subject received the two treatments, dissolved in 450 ml of water, with 7 days allowed between each treatment. Tricreatine citrate (Creapure™ Citrate, AlzChem, Trostberg, Germany) contains 65 % w/w creatine. Results are shown as means together with standard deviation. Primary and derived variables were analyzed by repeated measures ANOVA. Where a significant effect of treatment was indicated, data were further compared using a Bonferroni post-hoc test. The threshold for significance was set at p < 0.05.

**Results**

**Fig. 1 (abstract P6):**
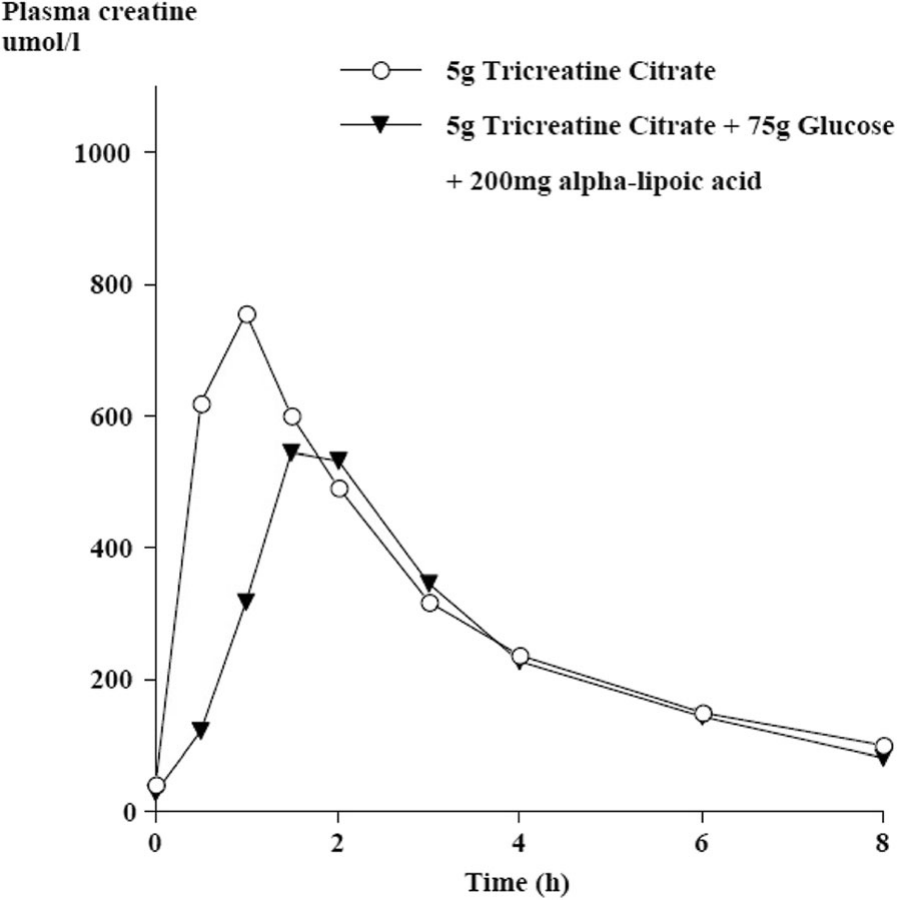
Mean plasma creatine concentration over 8 hours following ingestion of 5g tricreatine citrate (TCrC) and 5g tricreatine citrate + 75g glucose + 200mg alpha-lipoic acid (TCrC+Glu+ALA)

Mean peak concentration and AUC were lower in the TCrC + Glu + ALA group in comparison to TCrC (75.3 %, p < 0.05, and 82.2 % respectively). Figure 1 graphically displays the peak plasma Cr concentrations of the two study groups. The .5 and 1h plasma concentrations were significantly lower in the TCrC+Glu+ALA group in comparison to TCrC. Mean urinary creatine elimination over 8 hours was 26.5 ± 13.9 % of the dose administered (6.8 ± 3.6 mmol creatine) in the TCrC group, whereas co-administration with glucose and alpha-lipoic acid in the TCrC + Glu + ALA group reduced the mean creatine elimination to 17.2 ± 13.0 % (4.4 ± 3.3 mmol). These results are in keeping with previously published results showing an enhanced rate of creatine uptake into the muscle in the presence of raised insulin.

**Conclusion**

Lower plasma concentrations measured at 0.5 and 1 h and lower peak concentration are consistent with increased rate of uptake of creatine into muscle during this period mediated by an increase in circulating insulin in response to the 75 g glucose and 200 mg alpha-lipoic acid administered. It appears that the effect is declining after 1 h and may require a further administration of glucose. Rather than higher plasma concentrations, lower concentrations in this case are consistent with improved bioavailability. Changes in plasma creatine concentrations, either lower or higher, can only serve as an indication of bioavailability; conclusive evidence can only be obtained from muscle biopsies.

**Acknowledgement**

The authors would like to thank AlzChem, Trostberg, Germany for funding this research.

## P7 Effect of three different breakfast meals on energy intake and nutritional status in college-age women

### Molly M. Krause, Kiley A. Lavanger, Nina O. Allen, Allison E. Lieb, Katie A. Mullen, Joan M. Eckerson

#### Department of Exercise Science and Pre-Health Professions, Creighton University, Omaha, NE, USA

##### **Correspondence:** Molly M. Krause (mollykrause@creighton.edu) – Department of Exercise Science and Pre-Health Professions, Creighton University, Omaha, NE, USA

**Background**

Breakfast (BF) is associated with lower body weight and a more favorable nutrient intake profile. However, many young adults choose convenience over nutrition at BF, which may have negative effects on energy consumption and nutrient intake.

**Purpose**

The purpose of the current study was to compare the effect of an egg white (EW) BF to an isocaloric toaster pastry (TP) and habitual BF on energy intake and nutritional status in college-age women.

**Methods**

Using a randomized, crossover design, 31 women (X age ± SD = 20 ± 1 yr; BMI 22.9 ± 2.1 kg/m^2^; weight = 62.1 ± 7.4 kg) who regularly ate BF low in protein (<16 g) consumed two test BF and their habitual BF for 5 days (Tues-Sat): EW (Egg Beaters® Original) and two pieces of toast with spread (350 kcal), 2 low-fat Toaster Pastries (TP) (360 kcal), or their usual self-selected breakfast (SS). Participants completed a 24 hr dietary recall (ASA24®) for 3 of the 5 days during each BF trial to determine nutrient and energy intakes. Differences (p < 0.05) between BF treatments were analyzed using repeated measures ANOVA and compared to the 2015 Dietary Guidelines for Americans (DGA).

**Results**

There were no significant differences in total energy intake between the three BF treatments (SS = 1742 ± 69 kcal; TP = 1735 ± 63 kcal; EW = 1572 ± 63 kcal); however, there were differences in nutritional status. Total carbohydrate (CHO) and sugar intake were significantly (p < 0.05) lower during the EW BF compared to both SS and TP BF conditions. Compared to the TP BF, the EW BF resulted in significantly greater intakes of protein, calcium, and cholesterol, while the SS BF was higher in calcium, fiber, potassium and folate.

**Conclusion**

Although there were no significant differences in total energy intake between the three BF treatments, subjects consumed fewer calories during the EW BF (170 kcal) compared to the SS and TP BF, which may have long-term benefits for weight loss. For each BF condition, subjects did not meet dietary recommendations for several shortfall nutrients including folate, calcium, iron, potassium, fiber, and vitamins A, C, D, and E. Key recommendations of the DGA are to decrease sodium intake, control energy intake, reduce sugar consumption, and increase consumption of fruits, vegetables, whole grains, and fat-free and low-fat dairy products. During the TP BF, subjects consumed lower intakes of the nutrients of public health concern and higher amounts of added sugar and, therefore, a BF high in CHO is not recommended over a SS or EW BF. These results also suggest that additional strategies are needed to help college-age women achieve dietary recommendations established by the DGA.

**Acknowledgments**

Supported by ConAgra Foods, Inc.

## P8 Accuracy of the ASA24® Dietary Recall system for assessing actual dietary intake in normal weight college-age women

### Kiley A. Lavanger, Molly M. Krause, Nina O. Allen, Allison E. Lieb, Katie A. Mullen, Joan M. Eckerson

#### Department of Exercise Science and Pre-Health Professions, Creighton University, Omaha, NE, USA

##### **Correspondence:** Kiley A. Lavanger (KileyLavanger@creighton.edu) – Department of Exercise Science and Pre-Health Professions, Creighton University, Omaha, NE, USA

**Background**

Using trained personnel to obtain dietary recalls and manually enter the data is expensive and time consuming. The Automated Self-Administered 24-hr Dietary Recall (ASA24®) is a free web-based tool developed by the National Cancer Institute that may be a more practical method for collecting dietary data, but limited validation has been reported.

**Purpose**

Determine the accuracy of the ASA24® in a feeding study by comparing participant reported intakes to actual intakes for two different breakfast meals.

**Methods**

Thirty-one normal weight women (20 ± 1 yr, BMI = 22.9 ± 2.1 kg∙m^2^; weight = 62.1 ± 7.4 kg) volunteered to participate in this randomized, crossover study. Prior to data collection, subjects were screened for restrained eating and eating disorders, and were given instruction and a demonstration on how to use the ASA24®. In a free-living environment, participants randomly received two isocaloric breakfast meals for 5 d (Tuesday-Saturday): Egg whites (EW; Egg Beaters Original®) and two pieces of toast with spread (350 kcals) or 2 low-fat strawberry toaster pastries (TP; 360 kcals). The study breakfasts were pre-measured and weighed at an independent laboratory and all empty packaging and any uneaten food was returned to determine how much food was consumed (actual intake). Self-reported breakfast intake was assessed via three 24 hr dietary recalls (ASA24®) during each breakfast condition (two weekdays and one weekend). The nutrient intake for the actual breakfast intakes for the three recall days was analyzed using Eat Right® Dietary Analysis software. Agreement between the reported and actual intakes for total energy, and selected macro and micronutrients were examined by calculating the mean differences and percent differences (actual – reported/actual) for each breakfast. Dependent t-tests were also used to compare the actual energy intake for each breakfast to the self-reported values and differences were considered significant at p ≤ 0.05.

**Results**

For energy, macronutrients, fiber, and selected vitamins (A, E, C, folate) and minerals (calcium, iron, potassium, sodium) of public health concern, there was very good agreement between the actual and reported intakes for both the macro- and micronutrients for both breakfast conditions. The percent differences between the actual and self-reported values were all ≤ 10.5 % for the TP breakfast, and ranged from 1.6 % (Vitamin A; mean difference = -0.4 mcg) to -21.5 % (Fat; mean difference = -1.7 g) for the EW breakfast. Results for the t-tests showed that there was no significant difference in energy intake between the actual (261 ± 72 kcal) and reported (251 ± 105 kcal) values for the EW breakfast. However, the energy intake between actual and reported values for the TP breakfast was significantly different (355 ± 22 kcal vs. 383 ± 14 kcal, respectively). When corrections for energy intake were applied to the results for the ASA24® for the TP breakfast (360 kcal vs. 383 kcal), the differences between the mean values was no longer significant (p = 0.22).

**Conclusions**

Although corrections may be necessary for some food items in the ASA24® database, it appears to be a relatively accurate and inexpensive tool for assessing dietary intakes in large samples of subjects.

**Acknowledgments**

Supported by ConAgra Foods, Inc.

## P9 β-aminoisobutyric acid does not regulate exercise induced UCP-3 expression in skeletal muscle

### Elisa Morales, Jeffrey Forsse, Thomas Andre, Sarah McKinley, Paul Hwang, Grant Tinsley, Mike Spillane, Peter Grandjean, Darryn Willoughby

#### Department of Health, Human Performance, and Recreation, Baylor University, Waco, TX, USA

##### **Correspondence:** Darryn Willoughby – Department of Health, Human Performance, and Recreation, Baylor University, Waco, TX, USA

**Background**

Uncoupling proteins (UCP) are mitochondrial proteins that dissipate the electrochemical potential as heat by transporting protons back into the mitochondrial matrix. Five isoforms have been identified UCP-1, UCP-2, UCP-3, UCP-4, and UCP-5. UCP-1 is the predominant isoform expressed in white adipose tissue, while UCP-3 is the predominant isoform expressed in skeletal muscle. β-aminoisobutyric acid (BAIBA) is a recently discovered myokine induced by the transcription factor PGC-1α that causes browning of the white adipose tissue acting through PPARα_._ In rodents, BAIBA treatment has shown to increase UCP-1 expression in white adipose tissue and to reduce blood glucose and lipid levels, insulin resistance, lipid accumulation, and expression of enzymes involved in lipogenesis. In rodents, three weeks of exercise training increased by 5-fold BAIBA concentration in gastrocnemius and quadriceps and by 25-fold UCP-1 gene expression in WAT. In humans, a significant increase in plasmatic BAIBA concentrations was found after 20 weeks of exercise training_._ Taken this together, the goal of the present study was to evaluate the potential involvement of exercise-induced BAIBA on UCP-3 expression in human skeletal muscle. Furthermore, a secondary goal of the present study was to evaluate the possible effect of consuming a pre-exercise multi-macronutrient meal in the expression of these variables.

**Methods**

In a crossover design, untrained participants performed an endurance exercise session (350 kcal at 70 % of their VO_2_max) after two experimental conditions 1) consumption of a multi-macronutrient shake and 2) a fasting period of 8 h. Blood samples were taken at baseline, pre-exercise, post-exercise, 1 h, and 4 h post-exercise, while muscle biopsies were taken at the last four time points. UCP-3 protein and mRNA expression in skeletal muscle were measured by ELISA and RT-PCR, while BAIBA was measured in serum by using mass spectrometry.

**Results**

Despite a significant increase in UCP-3 mRNA (at Post p = 0.009, 1 h p = 0.013 and 4 h p = 0.028) and protein expression (at 1 h p = 0.040 and 4 h p = 0.007) in the shake condition, no significant time (p = 0.702, effect size = 0.019) or condition (p = 0.257, effect size = 0.012) main effects were observed in serum BAIBA concentration.

**Conclusions**

The expression of human skeletal muscle UCP-3 mRNA and protein expression as a result of exercise might be controlled by factors other than BAIBA. Further studies are needed to corroborate our results.

## P10 The ability of collegiate football athletes to adhere to sport-specific nutritional recommendations

### A. Jagim^1^, G. Wright^1^, J. Kisiolek^1^, M. Meinking^1^, J. Ochsenwald^1^, M. Andre^1^, M.T. Jones^2^, J. M. Oliver^3^

#### ^1^Exercise & Sport Science Department, University of Wisconsin – La Crosse, La Crosse, WI, USA; ^2^Division of Health and Human Performance, George Mason University, Fairfax, VA, USA; ^3^Kinesiology Department, Texas Christian University, Fort Worth, TX, USA

##### **Correspondence:** A. Jagim (ajagim@uwlax.edu) – Exercise & Sport Science Department, University of Wisconsin – La Crosse, La Crosse, WI, USA

**Background**

Pre-season football (FB) training camp typically consists of intense physical training that results in a high caloric expenditure. Limited data are available in regard to the nutritional habits of FB players during a period of pre-season training camp. However, evidence suggests that intake may not meet demand during this intense training period. Therefore, the purpose of this study was to assess the nutritional intake of collegiate FB players and compare them to recommendations put forth by the International Society of Sports Nutrition (ISSN) for strength and power athletes. Further, comparisons were made between linemen (L) and non-linemen (NL).

**Methods**

Seventeen (NL: 11, L: 6) NCAA-Division III football players (180.1 ± 60.1 cm; 99.1±6.01 kg; 19.3±8.6 % body fat) completed a 14-day training camp, which consisted of single and multi-day practices. The pre-season training period was 14 days and totaled 18 FB practices and 4 strength training sessions. Prior to the study, all participants attended an educational meeting in which a sports nutritionist provided verbal and written instructions for recording food intake. Dietary intake was self-reported throughout training camp using a commercially available food tracking program (*MyFitnessPal©, USA*). Daily average values were calculated for total and relative energy, protein, carbohydrate, and fat intake. These values were then compared to nutritional recommendations put forth by the ISSN for strength and power athletes. Recommended values of 60 kilocalories per kilogram of body weight per day (kg/d), 6.5 g/kg/d, and 1.8 g/kg/d for total energy, carbohydrates and protein, respectively, were used. A recommended fat intake equating to 30 % of the recommended energy intake was used for comparison.

**Results**

There was a significant deficit between average intake and recommend values for total energy (-21.0 ± 7.7 kcal/kg/d; p<0.001) and carbohydrates (-2.1 ± 1.2 g/kg/d; p<0.01) when expressed relative to body size for all athletes. There was a significant excess observed for daily fat intake compared to recommended values (22.0 ± 31.0 g/d; p = 0.01) with all players consuming more fat than recommend. There was a significant difference between position groups (L vs. NL) in the adherence to the nutritional guidelines for intake of energy (L: -26.2 ± 6.6 vs. NL: -18.2 ± 6.9 kcal/kg/d; p = 0.036), carbohydrates (L: -2.86 ± 0.87 vs. NL: -1.62 ± 1.22 g/kg/d; p=0.046), and protein (L: -0.487 ± 0.36 vs. NL: 0.15 ± 0.56 g/kg/d; p = 0.024) when expressed relative to body size.

**Conclusions**

The results demonstrate that the current players failed to meet the recommendations of the ISSN for total energy and carbohydrates while overeating fat. When intakes were assessed for position-specific differences, non-linemen were more successful than linemen at meeting the recommendations, specifically for calorie and carbohydrate intakes. Smaller institutions frequently do not have the resources to hire full-time nutritional staff; therefore, it may be beneficial for FB coaches to offer a nutrition-education program to ensure their players are meeting the energy requirements for their body size and level of training. Further, doing so may prevent reductions in training adaptations achieved during the off-season programming period.

## P11 A single session of low-volume high intensity interval exercise improves appetite regulation in overweight men

### Victor Araújo Ferreira Matos^1^, Daniel Costa de Souza^1^, Victor Oliveira Albuquerque dos Santos^1^, Rodrigo Alberto Vieira Browne^1^, Eduardo Caldas Costa^1^, Ana Paula Trussardi Fayh^2^

#### ^1^Department of Physical Education, Federal University of Rio Grande do Norte, Natal, RN, Brazil; ^2^Department of Nutrition, Federal University of Rio Grande do Norte, Natal, RN, Brazil

##### **Correspondence:** Ana Paula Trussardi Fayh (apfayh@yahoo.com.br) – Department of Nutrition, Federal University of Rio Grande do Norte, Natal, RN, Brazil

**Background**

High intensity interval exercise is a potential strategy to improve body composition and induce appetite suppression. Thereby, low-volume high intensity interval exercise (LV-HIIE) may arise as a possible alternative for weight loss in overweight and obese population. The purpose of this study was to determine the acute effect of LV-HIIE using an uphill walking protocol versus steady state exercise (SSE) on appetite regulation in overweight men.

**Methods**

This randomized crossover trial involves ten recreationally active men (Age: 26.4 ± 6.4 years, BMI: 27.4 ± 2.2 kg, waist circumference: 87.6 ± 7.2 cm) who were invited to complete two exercise conditions, separated by one week apart: LV-HIIE consists in 10 bouts x 60 seconds at 100 % peak incremental test speed/grade (S/Gmáx) + 60 seconds passive recovery and SSE consists in 20 minute treadmill walking at 50 % S/Gmáx. After ten hours overnight fasted, participants consumed a standard meal [4.5 kcal x body weight (Kg)] 45 minutes before performing exercise sessions. To determine the effect of exercise on appetite regulation during two exercise conditions, a visual analog scale (VAS) for hunger and satiety was used in four distinct moments: Fasted stated (fasted); Before exercise (pre); Immediately after exercise (post) and 40 minutes after exercise (rec 40’). Data was presented in mean ± SD. ANOVA 2-way with repeated measures and post hoc Bonferoni was used to assess subjective perception in hunger and satiety between moments and exercise conditions. Statistical significance was accepted at a p value of ≤ 0.05.

**Results**

**Fig. 2 (abstract P11):**
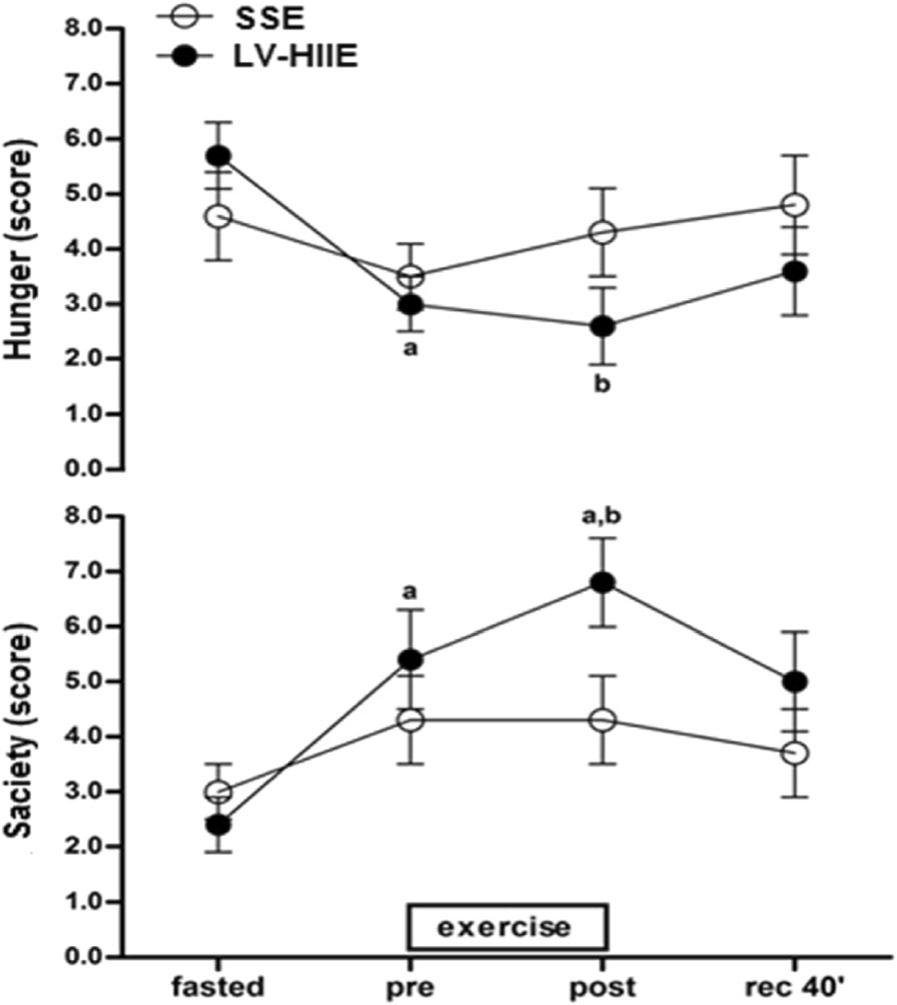
Effect of acute bout of low-volume high intensity interval exercise (LV-HIIE) and steady state exercise (SSE) on subjective perception of hunger and satiety in overweight men (n = 10). Values are presented in mean ± sd. ^a^ P <0.05 relative changes to the same condition in fasted. ^b^ P <0.05 relative changes compared to the same moment in SSE

The mean HR sustained during de LV-HIIE bouts was 167 ± 12 (~90 % HR máx) characterized a high intensity protocol, the speed and grade on treadmill was (6,5-7,5 km/h) and (4°-8°), respectively. There was a significant reduction in the subjective perception of hunger (p = 0.026) and increased satiety levels (p = 0.016) immediately after LV-HIIE compared to SSE. There was no significant difference between two exercise conditions in other moments. Figure 2 graphically contrasts the hunger and satiety scores in response to the SSE and LV-HIIE conditions.

**Conclusion**

A single bout of high-intensity interval exercise using an uphill walking protocol temporarily improves subjective perception of hunger and satiety in overweight subjects.

## P12 Acute effects of oral peppermint oil ingestion on exercise performance in moderately-active college students

### Suresh T. Mathews, Haley D. Bishop, Clara R. Bowen, Yishan Liang, Emily A. West, Rebecca R. Rogers, Mallory R. Marshall, John K. Petrella

#### Department of Nutrition and Dietetics, Department of Kinesiology, Samford University, Birmingham, AL, USA

##### **Correspondence:** Suresh Mathews (smathew1@samford.edu) – Department of Nutrition and Dietetics, Department of Kinesiology, Samford University, Birmingham, AL, USA

**Background**

Peppermint oil, isolated from *Mentha x piperita*, containing menthol and menthone as its major components, has recently attracted attention for its potential effects on improving athletic performance. Earlier studies show that consumption of peppermint oil for ten days improved exercise performance, spirometry parameters, blood pressure and respiratory rate. However, the acute effects of peppermint oil administration were equivocal, with one study demonstrating no effects on time of running, maximal oxygen consumption (VO2max), respiratory ventilation (VE), and respiratory exchange rate (RER), while another study showed improvements in grip force, standing vertical jump, and standing long jump.

**Methods**

In this study, we examined the acute effects of oral peppermint oil ingestion on oxygen consumption on twenty four (12 men and 12 women) healthy, university athletes, using a Velatron RacerMate bicycle coupled to a TrueOne 2400 metabolic cart. All participants initially completed a baseline oxygen consumption test cycling at a standard resistance of 150 watts for ten minutes. After a day’s rest, participants were randomized to receive either a single oral administration of 16 ounces of water or 0.05 ml of pure peppermint oil (Sigma) infused in 16 ounces of water. Immediately after this, participants cycled at a standard resistance of 150 min or ten minutes, and oxygen consumption was analyzed, similar to the baseline test.

**Results**

Participants did not report any discomfort or side-effects from consumption of peppermint water. Data analysis, using repeated measures ANOVA, showed that peppermint oil administration, prior to 10-min cycling at 150 watts, had no significant effect on the distance travelled, heart rate, VO2, VE, and RPE, in either men or women.

**Conclusions**

Our findings suggest that acute oral ingestion of peppermint oil may not have a beneficial effect on exercise performance.

## P13 Associations in body fat and liver triglyceride content with serum health markers in sedentary and exercised rats fed a ketogenic diet, Western diet or standard chow over a 6-week period

### A. Maleah Holland^1,2^, Wesley C. Kephart^1^, Petey W. Mumford^1^, C. Brooks Mobley^1^, Ryan P. Lowery^3^, Jacob M. Wilson^3^, Michael D. Roberts^1,4^

#### ^1^School of Kinesiology, Auburn University; Auburn, AL, USA; ^2^Department of Kinesiology, Augusta University; Augusta, GA, USA; ^3^Applied Science and Performance Institute; Tampa, FL, USA; ^4^Edward Via College of Osteopathic Medicine – Auburn Campus; Auburn, AL, USA

##### **Correspondence:** A. Maleah Holland (angholland@augusta.edu) – School of Kinesiology, Auburn University; Auburn, AL, USA

**Background**

Very low-carbohydrate (ketogenic) diets are gaining popularity as an effective fat loss method but the mechanisms behind this phenomena are not completely known. Our purpose was to examine potential associations between serum health-related biomarkers and fat deposition in sedentary and exercised rats fed a ketogenic (KD), Western (WD), or standard chow (SC) diet over 6 weeks.

**Methods**

Sedentary or exercised male Sprague-Dawley rats (~9-10 weeks of age) were provided isocaloric amounts of either a KD (5.2 kcal/g, 20.2 % protein, 10.3 % carbohydrate, 69.5 % fat; n=50), WD (4.5 kcal/g, 15.2 % protein, 42.7 % carbohydrate, 42.0 % fat; n=66), or SC (3.1 kcal/g, 24.0 % protein, 58.0 % carbohydrate, 18.0 % fat n=10) for 6 weeks with daily food intake and body weights recorded. Following euthanasia, 3 different fat depots were weighed, the liver was weighed and triglyceride content was assessed via the Folch method, and serum was collected for analysis of insulin, beta-hydroxybutyrate (βHB), and glucose levels.

**Results**

Serum insulin was positively associated with relative omental adipose tissue (OMAT) mass (r = 0.57, p < 0.001), and relative subcutaneous adipose tissue (SQ) mass (r = 0.31, p < 0.05). Serum βHB was positively associated with relative brown adipose tissue (BAT) mass (r = 0.38, p < 0.01), although no associations existed between serum βHB and OMAT or SQ mass. Serum glucose was positively associated with relative OMAT mass (r = 0.32, p < 0.05), and liver triglyceride content (r = 0.45, p < 0.01). Additionally, liver triglyceride content was positively associated with relative OMAT mass (r = 0.30, p < 0.05).

**Conclusions**

These data suggest that the positive associations between serum insulin and glucose with OMAT mass, SQ mass and liver triglyceride content implicate the KD-induced decrement in whole-body adiposity is likely due to a decrease in serum insulin and glucose rather than an increase in serum ketones.

## P14 Physiological changes following competition in male and female physique athletes: A pilot study

### Eric T. Trexler^1,2^, Katie R. Hirsch^2^, Bill I. Campbell^3^, Meredith G. Mock^2^, Abbie E. Smith-Ryan^1,2^

#### ^1^Human Movement Science Curriculum, University of North Carolina, Chapel Hill, NC, USA; ^2^Department of Exercise and Sport Science, University of North Carolina, Chapel Hill, NC, USA; ^3^Performance & Physique Enhancement Laboratory, University of South Florida, Tampa, FL, USA

##### **Correspondence:** Eric Trexler, (trexlere@live.unc.edu) – Human Movement Science Curriculum, University of North Carolina, Chapel Hill, NC, USA

**Background**

Previous studies in physique athletes (i.e., bodybuilding, figure, physique, and bikini competitors) have suggested that contest preparation is associated with unfavorable physiological and psychological effects. The purpose of the current study was to evaluate changes in body composition, metabolic rate, circulating hormones, and psychological factors in physique athletes before and following competition.

**Methods**

Natural physique athletes (7 male, 8 female) reported to the laboratory within one week prior to competition (T1), within one week following competition (T2), and 4-6 weeks following the competition (T3). Body composition (body fat percentage [BF%], fat mass [FM], lean mass [LM]) was assessed via amplitude-mode ultrasonography using a 7-site skinfold equation. Resting metabolic rate (RMR) was assessed via indirect calorimetry. Salivary leptin, testosterone, cortisol, ghrelin, and insulin were measured. Dietary intake was recorded prior to each visit. Mood state (Profile of Mood States [POMS]), and eating behaviors (Eating Attitudes Test 26 [EAT-26], Eating Disorder Examination Questionnaire [EDE-Q]) were assessed at T1 and T3. For a subset of athletes (n=8), bioelectric impedance spectroscopy (BIS) was used to measure fluctuations in total body water (TBW) from T1 to T2.

**Results**

Intakes of carbohydrate, fat, and calories increased from T1 to T2 and T3 (p<0.05), but protein intake did not. Changes were observed for weight (T1 = 65.4±12.2 kg, T2 = 67.4±12.6, T3 = 69.3±13.4; T3>T2>T1, p<0.05), BF% (T1 = 12.5±7.1 %, T2 = 12.5±6.9, T3 = 14.9±8.0; T3>T1 and T2, p<0.05), LM (T1 = 57.6±13.9 kg, T2 = 59.4±14.2, T3 = 59.3±14.2; T2 and T3>T1, p<0.01), and FM (T1 = 7.7±4.4 kg, T2 = 8.0±4.4, T3 = 10.0±6.2; T3>T1 and T2, p<0.001). TBW increased from T1 to T2 (Δ = 1.9±1.3 L, p<0.01). Baseline RMR was 1612.1±265.7 kcal/day (92.0 % of predicted; range=73.2–113.8 %), but increased at T2 (1881.1±329.1, 105.3 % predicted; p<0.01) and T3 (1777.6±256.9, 99.6 % predicted; range=80.4–119 %; p<0.001), with no differences between T2 and T3. Cortisol was higher at T2 (0.41±0.31 μg/dL) than T1 (0.34±0.31) and T3 (0.35±0.27; p<0.001). Insulin, ghrelin, and leptin did not change significantly. In males, testosterone at T3 (186.6±41.3 pg/mL) was greater than T2 (148.0±44.6, p=0.04), with T3 vs. T1 (147.4±33.6) approaching significance (p=0.056). Questionnaires indicated improvements from T1 to T3 in mood disturbance (POMS; 20.9±29.0 vs. -6.2±14.7, p<0.001) and disordered eating symptom scores (EDE-Q; 1.75±0.97 vs. 1.16±0.58, p=0.02), and identified amenorrhea in 4 of 8 females. Changes in RMR from T1 to T3 were positively associated with ΔBF% (r=0.59, p=0.04) and T3 protein intake (r=0.60, p=0.04); changes in male testosterone were inversely associated with ΔBF%, ΔFM, and Δweight (r= -0.81– -0.88; p=0.02–0.05).

**Conclusions**

Results demonstrate modest adverse effects of contest preparation on testosterone, RMR, menstrual cycle, and mood state. Increases in TBW were observed within days of competition, while weight gained in the following 4-6 weeks was primarily FM. Pre-competition RMR suppression is widely variable, markedly reversed by acute overfeeding, and appears to revert toward normal levels following competition. Future research should investigate the full time course of physiological recovery, and evaluate dietary strategies to facilitate recovery and optimize body composition.

**Acknowledgements**

This study was supported by a grant from the BioLayne Foundation

## P15 Relationship between cognition and hydration status in college students at a large Southwestern university

### Kate Zemek, Carol Johnston

#### Arizona State University, Phoenix, AZ USA

##### **Correspondence:** Kate Zemek (kzemek@asu.edu) – Arizona State University, Phoenix, AZ USA

**Background**

While water’s essentiality to the human body is widely known, it’s nutrient status is often overlooked. Additionally, it is known that mild dehydration can impair performance, mood and cognitive function. Increased heat stress and insensible water loss makes those living in hot, arid climates particularly at risk for dehydration. However, to date, no intervention trials that target fluid consumption to increase cognitive function among healthy U.S. college students in the desert southwest have been conducted. To inform if there is need for such an intervention, we examined hydration status and cognition in college students at a large Southwest university.

**Methods**

Currently enrolled undergraduate and graduate students aged 18-40 years with a BMI of 18.5-35 kg/m^2^ were recruited over a five-month period (December 2015-April 2016). Cognition was measured using four validated methods: Trail-Making Tests A & B (cognitive flexibility; completion time trial B-completion time trial A), the ruler drop test (reaction time based on distance) the Montreal Cognitive Assessment (MoCA; a battery assessment validated to detect mild cognitive impairments), and Brain Baseline (a tablet based application from which five validated tests were selected). Hydration assessment was measured using four validated tests: urine specific gravity (USG; euhydration <1.020), a validated color chart (color intensity numbered 1 to 8; color matches 4 to 8 indicate dehydration), bioelectrical impedance (Tanita), and hematocrit plus hemoglobin.

**Results**

Of the four cognitive methods utilized, only one produced a statistically significant result. There was a weak relationship between cognitive flexibility and hydration status (r=0.272, p=.039) based on urine color, after controlling for age and gender. Preliminary data on the hydration status of 24 subjects was shared at the Experimental Biology Meeting 2016. The completed study included 60 college students (46 F; 23.6 ± 4.9 y). Thirty percent of students assessed were dehydrated (n=18) using a score >4 on the color chart and 37 % (n=22) using ≥1.020 USG. An additional 17 % (n=10) showed signs of dehydration using a score >3 on the color chart and 30 % (n=18) using USG >1.010. Additionally, the urine specific gravity measure and urine color chart were highly correlated (r=0.875, p<.001).

**Conclusions**

Based upon the findings that potentially 47-67 % of college students in the desert southwest could benefit from increased fluid consumption, we believe there is need for an intervention study in this population. Furthermore, the increased cognitive demands and required cognitive flexibility of typical college students provides additional support for such a trial.

## P16 Whey protein-derived exosomes increase protein synthesis in C2C12 myotubes

### C. Brooks Mobley^1^, Petey W. Mumford^1^, David D. Pascoe^1^, Christopher M. Lockwood^3^, Michael E. Miller^2^, Michael D. Roberts^1^

#### ^1^School of Kinesiology, Auburn University, Auburn, AL, USA; ^2^Harrison School of Pharmacy, Auburn University, Auburn, AL, USA; ^3^Lockwood, LLC, Salt Lake City, Utah, USA

##### **Correspondence:** Michael D. Roberts (mdr0024@auburn.edu) – School of Kinesiology, Auburn University, Auburn, AL, USA

**Background**

Essential amino acids (EAA), L-leucine (LEU), and whey protein (WP) are potent anabolic nutrients that increase muscle protein synthesis (MPS). Previous literature has posited that the driving force behind WP’s anabolic potential is its high LEU content. The primary LEU transporter in skeletal muscle is large neutral amino acid transporter (LAT1). Potential knockdown of LAT1 would, in theory, attenuate the MPS response in WP and LEU-treated myotubes. However, other nanoparticles, specifically exosomes (EXO), are present in WP (WP-EXO) that may also facilitate an anabolic response within skeletal muscle. Herein we tested whether, 1) WP, EAA, or LEU alone (at concentrations equal to that in WP treatments) elicited similar increases in MPS, 2) whether different doses of WP or LEU alone (at concentrations equal to that in WP treatments) elicited similar increases in MPS, 3) whether LAT1 knockdown abrogated WP- and LEU-mediated increases in MPS, and 4) whether WP-EXO promoted alterations in MPS in post-differentiated myotubes.

**Methods**

Specifically, we tested whether: AIM 1) 3-h and 6-h treatments of hydrolyzed WP, EAA, or LEU affected MPS, and whether 6-h treatments of low-, medium-, or high doses of hydrolyzed WP versus LEU affected MPS; AIM 2) knockdown of LAT1 affected hydrolyzed WP- and LEU-mediated changes in MPS and/or mammalian target of rapamycin (mTOR) signaling responses following 6- h treatments; AIM 3) hydrolyzed WP-EXO affected MPS and/or mTOR signaling responses compared to untreated (CTL) myotubes following 6-h, 12-h and 24-h treatments. For all treatments, 7-days post-differentiated C2C12 myotubes were examined.

**Results**

In AIM 1, 6-h hydrolyzed WP treatments increased MPS compared to CTL (+46 %, p=0.001), LEU (+24 %, p<0.05) and EAA (+25 %, p<0.05) treatments. Moreover, the 6-h low-, medium-, and high hydrolyzed WP treatments increased MPS ~40-50 % more compared to corresponding LEU doses treatments (p<0.05, p<0.001 and p<0.001, respectively). In AIM 2 (LAT1 shRNA-transfected myotubes), 6-h hydrolyzed WP treatments increased MPS compared to CTL (+18 %, p<0.05) and LEU (+19 %, p<0.05) treatments. In AIM 3, hydrolyzed WP-EXO treatments increased MPS over CTL treatments at 12-h (+18 %, p<0.001) and 24-h (+45 %, p<0.05), respectively.

**Conclusion**

WP-EXO treatments did not appear to operate through mTOR signaling to increase MPS, although these treatments increased eukaryotic initiation factor (eIF) 4A mRNA and protein levels. Bovine-specific miRNAs following 24-h WP-EXO treatments were enriched in myotubes (chiefly bta-miR-149-3p, bta-miR-214, bta-miR-2881), but were not related to any hypertrophic gene targets that we are aware of. To summarize, whey protein-derived exosomes increase skeletal muscle protein synthesis *in vitro*, and this may be related to an unknown mechanism that increases in translation initiation factors rather than enhancing mTOR signaling or the involvement of bovine-specific miRNAs.

## P17 The effect of three different energy drinks on 1.5-mile running performance, oxygen consumption, and perceived exertion

### Gabriel J. Sanders^1^, Willard Peveler^1^, Brooke Warning^1^, Corey A. Peacock^2^

#### ^1^Northern Kentucky University, Highland Heights, KY, USA; ^2^Nova Southeastern University, Fort Lauderdale, FL, USA

##### **Correspondence:** Gabriel J. Sanders (sandersg1@nku.edu) – Northern Kentucky University, Highland Heights, KY, USA

**Background**

Some energy drink manufacturers claim that their products can increase athletic performance. However, there are no studies to assess and compare the effect of these energy drinks on 1.5-mile time trial performance, oxygen consumption (VO_2_), and ratings of perceived exertion (RPE) while running on a treadmill. If energy drinks improve performance, time trial performance and RPE would likely be reduced during any near maximum effort bout of training.

**Methods**

Thirteen (22.3 ± 4.3 years old) participants completed the study. All participants completed a total of four randomized, 1.5-mile time trial conditions on a treadmill in which the speed and time was blinded. For each condition, participants blindly ingested an energy drink or placebo then resting in a seated position for one hour. Following one hour of rest, participants ran on a treadmill (with time and speed blinded) as fast as possible until they completed 1.5 miles. Throughout each condition, VO_2_ (ml^.^kg^-1.^min^-1^), time (minutes and seconds) and RPE (6-20 Borg Scale) were recorded at every 0.5-mile marker (0.5, 1.0, and 1.5 mile) until each participant reached 1.5 miles. The following drinks were ingested prior to exercise: 12 oz. Placebo (Squirt), 16 oz. Red Bull®, 16 oz. Monster Energy ®, 2 oz. 5-hour ENERGY®.

**Results**

Analysis of variance revealed there was no significant main effect of energy drinks on time trial performance (Placebo 15.9 ± 0.7 minutes; Red Bull 15.4 ± 0.7 minutes; Monster 16.4 ± 0.8 minutes; 5-hour 15.5± 0.7 minutes; *P* = .631), average VO_2_ (Placebo 37.6 ± 1.7 ml^.^kg^-1.^min^-1^; Red Bull 37.6 ± 2.1 ml^.^kg^-1.^min^-1^; Monster 36.9 ± 2.4 ml^.^kg^-1.^min^-1^; 5-hour 36.6 ± 2.1 ml^.^kg^-1.^min^-1^; *P* = .895), and RPE (Placebo 13.6 ± 0.6; Red Bull 13.3 ± 0.5; Monster 13.7 ± 0.5; 5-hour 13.2 ± 0.5; *P* = .265).

**Conclusions**

Energy drinks do not appear to improve running economy measured via VO_2_, 1.5-mile running performance nor do they appear to improve feelings of perceived exertion during a 1.5-mile running time trial on a treadmill. Given that no significant reductions were found in VO_2_, running time and RPE, the current results do not support the notion that energy drinks boost performance, at least aerobic exercise performance. Additional research is needed to assess if drinks can improve anaerobic capacity or high intensity bouts of exercise. Time to exhaustion exercise may also reveal if these energy drinks can, in fact, improve exercise performance.

## P18 The Ketogenic diet improves rotarod performance in young and older rats

### Wesley C. Kephart^1^, Petey W. Mumford^1^, Ryan P. Lowery^2^, Michael D. Roberts^1^, Jacob M. Wilson^2^

#### ^1^School of Kinesiology, Auburn University, Auburn, AL, USA; ^2^Applied Science and Performance Institute, Tampa, FL, USA

##### **Correspondence:** Wesley C. Kephart (wck0007@tigermail.auburn.edu) – School of Kinesiology, Auburn University, Auburn, AL, USA

**Purpose**

Rotarod testing evaluates balance, grip strength and motor coordination in rodents. The purpose of this study was to examine the effects of a 9 week low carbohydrate ketogenic diet (KD) or supplemental ketone salt supplementation on body mass, feed efficiency, and rotarod performance in differentially-aged rodents.

**Methods**

This investigation was performed in a group of young and old rats (n=24 each). Male Fischer rats (4 mo. age and 10 mo. of age) were dually-housed and provided isocaloric amounts of KD (5.2 kcal/g, 23.1 % protein, 9.6 % carbohydrate, and 65.3 % fat, n=8 each age group) or standard rodent chow (3.1 kcal/g, 24 % protein, 58 % carbohydrate, 18 % fat, n=8 each age group) with sodium β-hydroxybutyrate mineral salts (STD+K) or standard chow only (STD, n=8 each age group). The ketone salts (5.8 kcal/g) were added to water bottles attempting to deliver ~1.2 g/ day for the first week, then 0.6 g/day for the remaining duration. Bodyweight was recorded weekly, left-over food was weighed daily, and rotarod performance was tested on the after 9 weeks of each respective diet intervention.

**Results**

For the younger cohort, bodyweights were not different between groups (p=0.429). Dietary fat consumption was as follows: KD>STD>STD+K (p<0.01). Dietary carbohydrate consumption was as follows: STD>STD+K>KD (p<0.01). Protein consumption was not different between KD and STD, but STD+K consumed less overall protein (p<0.01). Kilocalorie consumption was greatest in KD and not differential between STD and STD+K (p<0.01). Feed efficiency was greatest in STD, but similar in KD and STD+K (p<0.01). KD performed the rotarod test 72 s and 93 s longer than STD+K and STD, respectively (p<0.05). For the older cohort, the bodyweights were not different between groups (p=0.162). Fat consumption was the greatest in the KD, but not different between STD and STD+K (p<0.01). Again, KD consumed the least carbohydrates and the other two groups were similar (p<0.01). Protein consumption was not different between groups (p=0.983). Kilocalorie consumption was similar in KD and STD+K, but lowest in STD (p<0.01). Feed efficiency was as follows: KD<STD<STD+K (p<0.01). The KD group performed the rotarod for 12 s longer than STD and STD+K (p=0.064).

**Conclusions**

The KD seems to aid in rotarod performance compared to a control diet and supplemental ketone salts. More research is needed in order to determine if ketogenic diets can reduce muscle strength losses or aid in balance and coordination with aging.

**Acknowledgments**

Funding for this project was provided in large part by J.M.W. and R.P.L. from the Applied Science and Performance Institute and partially from discretionary laboratory funds provided by M.D.R.

## P19 Absorption of bonded arginine silicate compared to individual arginine and silicon components

### David Sandler^1^, Sara Perez Ojalvo^2^, James Komorowski^2^

#### ^1^StrengthPro Inc., Golden, CO, USA; ^2^Nutrition 21, LLC, Purchase, NY, USA

##### **Correspondence:** David Sandler (david@strengthpro.com) – StrengthPro Inc., Golden, CO, USA

**Background**

The purpose of this preclinical study was to compare the absorption of arginine and silicon from inositol-stabilized arginine silicate (ASI; Nitrosigine®) and equivalent amounts of arginine, silicon and inositol given as individual components in a single-dose. ASI is a sports nutrition ingredient that has been clinically shown to significantly enhance blood arginine levels up to 6 hours post-dose, while an equivalent dose of arginine from arginine HCl increased blood arginine for only one hour, showing ASI is a more bioavailable source of arginine. ASI has also been shown to significantly increase silicon levels, as well as nitric oxide, a key factor in increasing blood flow. Prior clinical studies have demonstrated significant improvements in many sports and exercise related endpoints including energy, focus and markers of muscle recovery. The current preclinical study was designed to evaluate the potential differences in arginine and silicon absorption between bonded ASI and the individual components, which may help explain the increased absorption and efficacy in sports and exercise applications.

**Methods**

Female Wistar rats (n=7 in each group; 8 weeks-old) were reared at the temperature of 22 ± 2 °C and a 12/12 hour light/dark cycle and randomized into four groups. An arthritis/joint health model was used in this study to obtain data on absorption and markers of joint health and inflammation, an important sports nutrition area. Only data on absorption is presented in this report. The four groups contained a control group (I), an arthritic control group (II), an arthritic group (III) supplemented with arginine HCl + silicon + inositol (A+S+I) separately but with equivalent doses to those in ASI, and an arthritic group (IV) supplemented with bonded inositol-stabilized arginine silicate (ASI) at a dose of 1.81 g/kg/day, equivalent to a human dose of 1500 mg. Blood was collected from the tail vein pre and post-dose to compare the absorption of the ASI complex (Group IV) and the simple mixture of the components (Group III). Joint tissue samples from Day 29 were also analyzed for arginine and silicon content.

**Results**

Serum arginine, serum silicon, joint tissue arginine and joint tissue silicon were all significantly greater in the ASI group (IV) compared to the control groups (I and II) and the A+S+I group (III) (p<0.05; Table 1). ASI supplementation significantly increased arginine and silicon bioavailability not only in the blood, but also in joint tissue, to a greater degree than the supplementation of equivalent doses of arginine, silicon and inositol as individual components.

**Conclusions**

This preclinical study showed that ASI is a more bioavailable source of arginine and silicon than the individual components, supporting its position as an active sports nutrition ingredient enhancing energy, focus and markers of muscle recovery. These results showing increased absorption of arginine and silicon from ASI, help explain and support the enhanced efficacy of ASI in sports and athletic applications.

**Table 1 (abstract P19):** Serum and Joint Tissue Arginine and Silicon Levels

Variable	Control (I)	Arthritis (II)	Arginine HCl + Silicon + Inositol (III)	ASI Complex (IV)
Serum Arginine (nmol/L)	1.10±0.07^c^	0.77±0.06^d^	1.33±0.03^b^	1.56±0.01^a^
Serum Silicon (mcg/L)	131.86±6.85^c^	109.00±2.14^c^	580.71±20.94^b^	712.86±18.14^a^
Joint Tissue Arginine (pmol/mg)	537.29±24.58^c^	463.71±28.50^c^	682.57±23.77^b^	793.14±15.99^a^
Joint Tissue Silicon (mcg/g)	0.56±0.03^bc^	0.44±0.03^c^	0.64±0.03^b^	0.79±0.04^a^

*Different letters are significant at p<0.05

## P20 Effects of a high (2.4 g/kg) vs. low/moderate (1.2 g/kg) protein intake on body composition in aspiring female physique athletes engaging in an 8-week resistance training program

### Bill I. Campbell, Danielle Aguilar, Andres Vargas, Laurin Conlin, Amey Sanders, Paola Fink-Irizarry, Layne Norton, Ross Perry, Ryley McCallum, Matthew R. Wynn, Jack Lenton.

#### University of South Florida, Performance & Physique Enhancement Laboratory, Tampa, FL, USA

##### **Correspondence:** Bill Campbell (bcampbell@usf.edu) – University of South Florida, Performance & Physique Enhancement Laboratory, Tampa, FL, USA

**Background**

Aspiring female physique athletes are often encouraged to ingest relatively high levels of dietary protein in conjunction with their resistance-training programs. However, there is little to no research investigating higher vs. lower protein intakes in this population. The purpose of this investigation was to compare the effects of a high protein diet vs. a low protein diet in resistance trained, aspiring female physique athletes.

**Methods**

17 resistance-trained female subjects (21.2±2.1 years; 165.1±5.1 cm; 61±6.1 kg) participated in this investigation. At baseline and following 8-weeks of a periodized daily undulating resistance-training program (DUP), participants were assessed for body composition (body weight [BW], fat mass [FM], body fat % [BF%], and lean body mass [LBM]). After baseline testing, participants were matched according to total FM and randomized to the high protein group (HP; n = 8) or the low/moderate protein group (LP; n = 9). Participants in the high protein group were instructed to ingest at least 2.4 grams of protein/kg body mass per day and participants in the low protein group were instructed to ingest no more than 1.2 grams of protein/kg body mass. There were no restrictions or guidelines placed on dietary CHO or Fat intake during the study intervention for either group. Body composition was assessed via ultrasound (A mode, 2.5-MHz transmitter). The DUP program consisted of two lower body and two upper body workouts conducted a total of 4 times per week for 8 weeks. Data were analyzed via a 2-factor [2x2] between-subjects repeated measures analysis of variance (ANOVA). The criterion for significance was set at p ≤ 0.05.

**Results**

No differences existed between the two groups for any body composition measure at baseline. The repeated measures ANOVA revealed a significant group x time interaction for lean body mass (p = 0.009) favoring the high protein group. Specifically, lean body mass increased from 47.1 ± 4.5 kg to 49.2 ± 5.4 kg and from 48.1 ± 2.7 kg to 48.8 ± 2 in the high and low protein groups, respectively. There were no differences between the groups for BW (HP: Pre = 61.2 ± 7.9 kg, Post = 62.2 ± 8.2 kg, LP: Pre = 61.4 ± 4.4 kg, Post = 61.2 ± 4.6 kg, p = 0.120); FM (HP: Pre = 14.1 ± 3.6 kg, Post = 13.0 ± 3.3 kg, LP: Pre = 13.2 ± 3.7 kg, Post = 12.5 ± 3.0 kg, p = 0.678), or BF% (HP: Pre = 22.7 ± 3.0 %, Post = 20.7 ± 3.1 %, LP: Pre = 21.4 ± 5.2 %, Post = 20.3 ± 3.9 %, p = 0.349).

**Conclusions**

In aspiring female physique athletes, it appears as if a higher protein diet (at least 2.4 g/kg day) is superior to a lower protein diet in terms of increasing lean body mass in conjunction with a DUP program. It is important to note that these findings were observed during a non-dieting phase of training – equivalent to a physique athlete’s off-season. There does not appear to be any advantages to a higher protein diet in relation to inducing fat loss under the same conditions.

**Acknowledgments**

This study was supported by Dymatize Nutrition Sport Performance Institute Dymatize® by providing funds and product.

## P21 Effects of a high (2.4 g/kg) vs. low/moderate (1.2 g/kg) protein intake on maximal strength in aspiring female physique athletes engaging in an 8-week resistance training program

### Bill I. Campbell, Chris Gai, Seth Donelson, Shiva Best, Daniel Bove, Kaylee Couvillion, Jeff Dolan, Dante Xing, Kyshia Chernesky, Michael Pawela, Andres D. Toledo, Rachel Jimenez

#### University of South Florida, Performance & Physique Enhancement Laboratory, Tampa, FL, USA

##### **Correspondence:** Bill Campbell (bcampbell@usf.edu) – University of South Florida, Performance & Physique Enhancement Laboratory, Tampa, FL, USA

**Background**

Aspiring female physique athletes are often encouraged to ingest relatively high levels of dietary protein in conjunction with their resistance training programs. However, there is little to no research investigating higher vs. lower protein intakes and its effect on maximal strength in this population. The purpose of this investigation was to compare the effects of a high protein diet vs. a low protein diet on maximal squat and deadlift strength in resistance trained, aspiring female physique athletes.

**Methods**

17 resistance-trained female subjects (21.2 ± 2.1 years; 165.1 ± 5.1 cm; 61 ± 6.1 kg) participated in this investigation. At baseline and following 8-weeks of a periodized daily undulating resistance-training program DUP), participants were assessed for maximal strength on the back squat and deadlift. After baseline testing, participants were matched according to total fat mass and randomized to the high protein group (HP; n = 8) or the low/moderate protein group (LP; n = 9). Participants in the high protein group were instructed to ingest at least 2.4 grams of protein/kg body mass per day and participants in the low protein group were instructed to ingest no more than 1.2 grams of protein/kg body mass. There were no restrictions or guidelines placed on dietary CHO or Fat intake during the study intervention for either group. Maximal back squat and deadlift strength was assessed prior to and following the 8-week DUP training program. The DUP training program consisted of two lower body and two upper body workouts per week for an 8-week period. Data were analyzed via a 2-factor [2x2] between-subjects repeated measures analysis of variance (ANOVA) using SPSS version 22.0. The criterion for significance was set at p ≤ 0.05.

**Results**

No differences existed between the two groups for either strength measure at baseline. The repeated measures ANOVA revealed a main effect for time for both the back squat (p < 0.001) and deadlift (p < 0.001) exercises, but no group x time interactions for either the back squat (p = 0.887) or the deadlift (p = 0.721). Specifically, back squat increased from 69.3 ± 18.4 kg to 78.7 ± 16.0 kg and from 72.0 ± 15.1 kg to 81.8 ± 20.1 kg in the high and low protein groups, respectively. Deadlift increased from 86.9 ± 14.8 kg to 102.8 ± 18.5 kg and from 97.2 ± 16.7 kg to 111.4 ± 17.6 kg in the high and low protein groups, respectively.

**Conclusions**

In aspiring female physique athletes, it appears as if a higher protein diet (at least 2.4 g/kg day) offers no advantage in terms of increasing maximal back squat and deadlift strength.

**Acknowledgments**

This study was supported by Dymatize Nutrition Sport Performance Institute Dymatize® by providing funds and product.

## P22 Monitoring of female collegiate athletes over a competitive season reveals changes in nutritional biomarkers

### M. Rabideau^1^, A. Walker^1^, J. Pellegrino^1^, M. Hofacker^1^, B. McFadden^1^, S. Conway^1^, C. Ordway^1^, D. Sanders^1^, R. Monaco^1,2^, M.S. Fragala^3^, S.M. Arent^1^

#### ^1^IFNH Center for Health & Human Performance, Rutgers University, New Brunswick, New Jersey, USA; ^2^Department of Intercollegiate Athletics, Rutgers University, New Brunswick, New Jersey, USA; ^3^Sports and Human Performance Diagnostics, Quest Diagnostics, New Brunswick, New Jersey, USA

##### **Correspondence:** M. Rabideau (mmr213@scarletmail.rutgers.edu) – IFNH Center for Health & Human Performance, Rutgers University, New Brunswick, New Jersey, USA

**Background**

Recent advancements in biomarker application have created new opportunities for optimizing athletic performance. With these technological advancements, monitoring biomarkers to assess health status is more practical than ever. While typically plasma levels of nutrients are tested after initial suspicions of performance or health issues, generally biomarker monitoring has not been used as a preventative measure. This study was performed to assess the changes in nutritional biomarkers during a competitive season and determine how this technology can be applied to athlete health evaluation.

**Methods**

Division I female collegiate athletes from two power-endurance sports (N=48; M_age_=19.9+/- 1.1 yrs; M_weight_=64.5 kgs; M_height_=65.4 in) participated in blood draws at the start of preseason (T1), the first quarter of the competitive season (T2), the midpoint of the competitive season (T3), and immediately after the competitive season (T4). The athletes arrived fasted and euhydrated in the morning ~18 h after a game. Iron (Fe), Hemoglobin (Hgb), Ferritin (Fer), Percent Saturation (%Sat), Total Vitamin D (VitD), Vitamin B12 (VitB12), Calcium (Ca), and Magnesium (Mg) were assessed using RM ANOVAs with simple contrasts.

**Results**

There were significant changes in Fe, Hgb, Fer, %Sat, VitD, VitB12, Ca, and Mg during the season (p<0.05). Markers of iron status decreased from T1 to T4 (ΔFe=-60.6+/-8.91 mcg/dL, p<0.005; ΔHgb=-0.61+/-0.13 g/dL, p<0.005; ΔFer=-8.69+/-1.53 ng/mL, p<0.005; %Sat=-14.83+/-2.32 %, p<0.005). VitD decreased from the T1 to T4 (ΔVitD=-7.44+/-1.62 ng/mL, p<0.005). VitB12 increased from T1 to T4 (ΔVitB12=44.98+/-18.83 pg/mL, p<0.05). Ca decreased from T1 to T2 (ΔCa= -0.15+/-0.05 mg/dL, p=0.005), increased from T2 to T3 (ΔCa=0.13+/-0.06 mg/dL, p<0.05), and slightly decreased from T3 to T4. Mg increased from T1 to T3 (ΔMg= 0.07+/-0.2 mg/dL, p<0.05) and decreased from T3 to T4 (ΔMg=-0.6+/-0.02 mg/dL, p<0.05).

**Conclusions**

Female athletes are susceptible to chronic iron-deficiency due to gender-specific metabolic demands during an athletic season. Despite the reported values not occurring outside broad general population ranges, athletes may be more sensitive to small fluctuations and require more nuanced recommendations, particularly in light of the impact on oxygen carrying capacity and recovery. In conjunction with the increase in Vitamin B12, the effects on iron status suggest metabolic disruption, potentially beginning preseason. Constant monitoring of biomarkers during an athletes’ training cycle can also provide opportunities for nutritional intervention. It appears that homeostatic disruption is particularly pronounced in the preseason because of the demanding workload and frequency and this effect either persists or increases in magnitude throughout the season. This application of biomarker evaluation could prevent nutritionally-associated performance decrements and negative health outcomes in the female athlete.

**Acknowledgments**

Supported by a grant from the Sports and Human Performance Diagnostics of Quest Diagnostics.

## P23 Comparison of prediction equations to indirect calorimetry in men and women athletes

### Jason D. Stone^1^, Andreas Kreutzer^1^, Jonathan M. Oliver^1^, Jacob Kisiolek^2^ and Andrew R. Jagim^2^

#### ^1^Exercise & Sport Performance Laboratory, Department of Kinesiology, Texas Christian University, Fort Worth, TX, USA; ^2^Exercise & Sport Science Department, University of Wisconsin – La Crosse, La Crosse, WI, 54603, USA

##### **Correspondence:** Jason Stone (j.stone@tcu.edu) **–** Exercise & Sport Performance Laboratory, Department of Kinesiology, Texas Christian University, Fort Worth, TX, USA

**Background**

Resting energy expenditure (REE) is a common component of weight loss and body composition management programs that when measured directly requires expensive laboratory equipment. An alternative approach to implementing REE as a strategy for weight management that does not impose the same financial burden is to use REE prediction equations. The applicability of these predictive equations potentially eludes healthy populations, particularly athletes, as the equations have primarily been developed in overweight or sedentary populations. Therefore, the purpose of this study was to determine the accuracy and potential gender differences of five different REE estimation equations in male and female athletes.

**Methods**

Twenty-one male (n = 21; 20 ± 1 yrs; 180.8 ± 6.3 cm; 97.7 ± 17.3 kg; 17.2 ± 8.5 % BF) and nineteen female (n = 19; 20 ± 1 yrs; 166.1 ± 5.2 cm; 64.5 ± 7.2 kg; 23.4 % ± 4.4 % BF) athletes were recruited to participate in one day of testing. Assessments comprised REE measurements via indirect calorimetry (*TrueOne® 2400 Metabolic Measurement system, ParvoMedics, Sandy, UT)* and body composition analyses via air displacement plethysmography (BODPOD, Cosmed, USA). Body densities acquired from the BODPOD and the Siri Equation were utilized to determine fat and fat-free mass. Athletes were instructed to come to testing fasted (>8 hrs.) as well as remove themselves from any strenuous exercise 48 hrs. prior to. In order for REE to be properly measured, athletes laid (supine) motionless for 15-20 minutes while maintaining conscious awareness. Data recordings were then obtained when criterion variables (i.e., absolute VO_2_) experienced less than a 5 % alteration every 5 minutes. Paired t-tests were selected to determine differences between indirect calorimetry and five REE prediction equations: 1) Nelson Equation; 2) Mifflin-St. Jeor Equation; 3) Cunningham Equation; 4) Harris-Benedict Equation; and 5) De Lorenzo Equation. Relationships between REE assessment strategies were determined via paired t-tests and bivariate Pearson correlations. An alpha level of p < 0.05 was used to determine statistical significance.

**Results**

REE as measured by indirect calorimetry was significantly higher than all five equations (p < 0.001) in the male athletes, although they were all positively correlated (p < 0.007) with indirect calorimetry (r = 0.572 to 0.684). In contrast, only the Nelson (p < 0.001), Harris-Benedict (p = 0.041), and De Lorenzo (p = 0.041) were significantly different from indirect calorimetry in women. The Nelson and Harris-Benedict equations underestimated REE, while the De Lorenzo overestimated. Similar to men, all five equations were positively correlated with indirect calorimetry in women (r = 0.606 to 0.739).

**Conclusions**

Our results suggest that despite being correlated, all REE prediction equations may fail to properly estimate REE within an acceptable range relative to indirect calorimetry in men. However, the Mifflin St-Jeor and Cunningham equation may be appropriately used in women athletes. Practitioners are cautioned against the use of REE prediction equations in athletes.

## P24 Regional variations in sweat-based electrolyte loss and changes in plasma electrolyte content in Division I female athletes over the course of a competitive season

### M. Hofacker^1^, A. Walker^1^, J. Pellegrino^1^, M. Rabideau^1^, B. McFadden^1^, S. Conway^1^, D. Sanders^1^, C. Ordway^1^, R. Monaco^2^, M.S. Fragala^3^, S.M. Arent^1^

#### ^1^IFNH Center for Health & Human Performance, Rutgers University, New Brunswick, New Jersey, USA; ^2^Dept. of Intercollegiate Athletics, Rutgers University, New Brunswick, New Jersey, USA; ^3^Sports and Human Performance Diagnostics, Quest Diagnostics, New Brunswick, New Jersey, USA

##### **Correspondence:** M. Hofacker (mhofacker@scarletknights.com) – IFNH Center for Health & Human Performance, Rutgers University, New Brunswick, New Jersey, USA

**Background**

Recommendations regarding fluid and electrolyte replenishment are primarily based on sweat-loss data obtained in males. Additionally, in an effort to simplify hydration assessment, sweat patches have been developed to replace more arduous whole-body washdowns, though regional differences have not been established. The purpose of the present study was to evaluate whether there are chronic changes in plasma electrolytes as a function of “high” vs “low” electrolyte concentration in sweat in high-level female athletes.

**Methods**

Division I female collegiate athletes (N = 29; M_age_ = 20 ±1.0 yrs; M_weight_ = 64.3 ± 2.7 kg; M_height_ = 166.4 ± 6.5 cm; M_VO2max_ = 48.1 ± 4.7) participated in blood draws at the beginning of preseason (T1), throughout the competitive season (T2 and T3), and at the very end of the season (T4). During a practice session lasting approximately 2 hrs, regional sweat collections were made from the forearm, chest, and navel using sweat patches. For both sweat and plasma, sodium (Na), potassium (K), chloride (Cl), calcium (Ca), and magnesium (Mg) concentrations were determined.

**Results**

There were significant differences in sweat electrolyte content that occurred as a function of patch site (p<.05). The greatest [K], [Ca], and [Mg] were found at the forearm (K = 6.65 ± 1.84 mM; Ca = 1.18 ± 0.53 mg/dL; Mg = 0.36 ± 0.19 mg/dL). The largest [Na] and [Cl] were produced at the navel (67.02 ± 20.25 mM and 56.74 ± 20.40 mM, respectively). [Electrolyte] across sites were summed to represent the total loss of each individual electrolyte. Individuals were classified as “high” or “low” concentration sweaters for each measure after calculating z-scores, using 0 as the split point. There were no differences between groups for any plasma [electrolyte] over the season (p>.05). However, there were significant increases in plasma [Na] and [Cl] from T1 to T3 (ΔNa = 1.57 ± 2.79 mM; ΔCl = 2.78 ± 3.26 mM) and an additional increase from T3 to T4 (ΔNa = 1.71 ± 2.75 mM; ΔCl = 1.08 ± 2.67 mM, p<.05). There was a significant increase in plasma [K] concentrations from T1 to T2 (ΔK = 0.27 ± 0.39 mg/dL) followed by a decrease from T2 to T4 (ΔK = -0.19 ± 0.33, p<.05). Similarly there was a significant increase in plasma [Ca] from T2 to T3 (ΔCa = 0.157 ± 0.34 mg/dL) followed by a decrease from T3 to T4 (ΔCa = -0.196 ± 0.33 mg/dL, p<.05). [K] and [Ca] decreases observed at T4 were still above baseline. There were no significant changes in plasma [Mg] throughout the season.

**Conclusions**

Regional variations in sweat [electrolyte] were observed in female athletes. However acute sweat composition does not appear to effect plasma values across an athletic season. Combined with the fact that most electrolytes were relatively maintained throughout the season or slightly increased, these results may suggest that these athletes adequately chronically replenish any electrolytes lost through sports drinks and sodium content in foods.

**Acknowledgements**

Funding provided by Sports and Human Performance Diagnostics of Quest Diagnostics.

## P25 In-season changes in plasma amino acid levels in Division I NCAA female athletes

### Ozlem Tok^1^, Joseph K. Pellegrino^1^, Alan J. Walker^1^, David J. Sanders^1^, Bridget A. McFadden^1^, Meaghan M. Rabideau^1^, Sean P. Conway^1^, Chris E. Ordway^1^, Marissa Bello^1^, Morgan L. Hofacker^1^, Nick S. Mackowski^1^, Anthony J. Poyssick^1^, Eddie Capone^1^, Robert M. Monaco^1,3^, Maren S. Fragala^2^, Shawn M. Arent^1^

#### ^1^Center for Health and Human Performance, Rutgers University, New Brunswick, New Jersey, USA; ^2^Sport & Human Performance Diagnostics, Quest Diagnostics, New Brunswick, New Jersey, USA; ^3^Department of Intercollegiate Athletics, Rutgers University, New Brunswick, New Jersey, USA

##### **Correspondence:** Ozlem Tok (ozlemtok@hotmail.com) – Center for Health and Human Performance, Rutgers University, New Brunswick, New Jersey, USA

**Background**

Changes in the plasma amino acid (AA) profile have been linked to prolonged fatigue and overreaching in athletes, though little has been done with the female athlete. As appearance of AA’s in the plasma may be indicative of protein turnover, assessment may be of use for helping determine recovery and repair status. The purpose of this study was to investigate changes in essential AA and specific AA associated with overreaching in female collegiate athletes throughout the competitive season.

**Methods**

Blood draws were completed on 48 Division I female collegiate athletes (M_age_=19.9±1.12 yrs, M_weight_=64.7±6.97 kgs, M_height_=166.09±5.38 cm) at the following time points: the start of preseason (T1), 4-weeks into the season (T2), midseason (T3), and immediately after the competitive season (T4). Athletes came in 18-36 hours after a game from T2-T4. All draws were performed following an overnight fast and in a euhydrated condition. Plasma concentrations of the essential amino acids plus glutamine, glutamate, and taurine were determined at each time point. RM ANOVA were conducted and simple contrast follow-ups done using T1 as the comparison.

**Results**

Significant time effects were seen for all AA (P<0.05). Concentrations were elevated for histidine (18 %, 15 %, 15 %) and Isoleucine (11 %, 22 %, 14 %) and reduced for taurine (-29 %, -21 %, -27 %) at all time points (P<0.05). Phenylalanine (11 %, 21 %) and tryptophan (22 %, 40 %) were increased at T3 & T4 (P<0.01); leucine (9 %, 10 %) and glutamine (15 %, 15 %) at T2 & T4 (P<0.05); methionine (17 %) and valine (7 %) at T3 and glutamic acid (4 %) at T4 only (P<0.05). Significant decreases were seen in Lysine (-10 %, -10 %) at T3 and T4 (P<0.05) and threonine (-9 %) at T4 only (P<0.05). All individual AA levels remained within normal ranges throughout the season.

**Conclusions**

While specific AA patterns differed across the season, many increased from baseline. Given the fasted state and time lapse from the last work bout, increased plasma AA levels may be due to endogenous sources as a result of elevated protein turnover and resultant aminoacidemia. Changes in specific amino acids may reveal unique information regarding tolerance to training. For example, elevations in BCAAs suggest the possibility of mobilization in an attempt to meet the energy demands of training. The increased tryptophan may reflect neurotransmitter changes and central fatigue. Decreased taurine may indicate its use in protection against muscle-tissue breakdown associated with free radical production and ischemia-reperfusion incurred from chronic training. Previous research from our laboratory has shown markers of aggregated stress/inflammation and muscle breakdown throughout the competitive season in these same athletes. Elevations in plasma AA within 36-hours of an intense training bout has been observed in previous studies. Provisions of high-quality protein in combination with training may help to maintain nitrogen balance and stave off net catabolism in athletes during a competitive collegiate season.

**Acknowledgements**

Supported by Sport & Human Performance Diagnostics, Quest Diagnostics

## P26 Effects of a ketogenic diet with exercise on serum markers of bone metabolism, IGF-1 and femoral bone mass in rats

### Petey W. Mumford^1^, A. Maleah Holland^1,2^, Wesley C. Kephart^1^, Ryan P. Lowery^3^, C. Brooks Mobley^1^, Romil K. Patel^1^, Annie Newton^4^, Darren T. Beck^4,1^, Michael D. Roberts^1,4^, Jacob M. Wilson^3^, Kaelin C. Young^4,1^

#### ^1^School of Kinesiology, Auburn University, Auburn, AL, USA; ^2^Department of Kinesiology and Health Sciences, Augusta University, Augusta, GA, USA; ^3^Applied Science and Performance Institute, Tampa, FL, USA; ^4^Edward Via College of Osteopathic Medicine – Auburn Campus, Auburn, AL, USA

##### **Correspondence:** Petey W. Mumford (Pwm0009@auburn.edu) – School of Kinesiology, Auburn University, Auburn, AL, USA

**Background**

Very low-carbohydrate (ketogenic) diets are becoming increasingly popular as weight loss interventions and have historically been used in the treatment of epileptic seizures. However, evidence in sedentary rodent models suggest that ketogenic diets (KD) are detrimental to the skeleton. It is unclear if exercising while on a KD can minimize bone-related deficits. Therefore, this study examined the effects of a KD, western (WD), and standard chow (SC) diet on markers of bone metabolism and femoral bone mass in exercising rodents.

**Methods**

Male Sprague-Dawley rodents (~9-10 weeks of age) were provided isocaloric amounts of either a KD (5.2 kcal/g, 20.2 % protein, 10.3 % carbohydrate, 69.5 % fat; n=50), WD (4.5 kcal/g, 15.2 % protein, 42.7 % carbohydrate, 42.0 % fat; n=66), or SC (3.1 kcal/g, 24.0 % protein, 58.0 % carbohydrate, 18.0 % fat n=10) for 6 weeks with energy intake (kcal) and body weights recorded. Rodents were provided a voluntary resistance running wheel and daily distances in kilometers (km) ran were recorded. After animal sacrifice, whole blood was collected via heart puncture and processed for serum analyses of Collagen Type I C-Telopeptide (CTX), Sclerostin, Insulin-like Growth Factor-1 (IGF-1), Alkaline Phosphatase, and phosphorus. Femurs were excised, cleaned of visible connective tissue and weighed. One-way ANOVA’s with Tukey post hoc analyses were used to determine mean differences between groups with an alpha level (α) of p ≤ 0.05. All data are reported as mean ± standard deviation.

**Results**

There were no significant differences in running distance (KD: 62.49 ± 45.73; WD: 53.91 ± 28.73; SC: 47.61 ± 26.59 km) or total kcals (KD: 3310.9 ± 171.3; WD: 3493.7 ± 109.5; SC: 3294.9 ± 197.7) consumed over the duration of the intervention. Additionally, there were no differences in body weights at baseline. However, after 6 weeks of intervention, KD rats weighed significantly (p=0.004) less than WD and SC (412.9 ± 25.1 vs. 476.2 ± 25.1 vs. 458.6 ± 47.5 grams, respectively). There were no differences in femur masses between groups. There were no significant group differences for serum CTX, IGF-1, or phosphorous. However, there were differences in serum alkaline phosphatase (p<0.001) (KD: 199.04 ± 57.94; WD: 73.5 ± 13.23; SC: 94.67 ± 9.59) and Sclerostin (p=0.041) (KD: 307.49 ± 65.22; WD: 404.03 ± 114.63; SC: 299.87 ± 65.10).

**Conclusions**

Our data suggest that resisted wheel running exercise may help to mitigate bone loss commonly reported in sedentary rodent / human ketogenic diet interventions. Future analysis should include more in depth bone density and geometrical measurements to confirm these findings.

**Acknowledgements**

This study was supported financially in part by a subcontract from The Applied Science and Performance Institute (to M.D.R. from J.M.W.) as well as laboratory start-up funds from M.D.R.

## P27 Casein supplementation in trained men and women: morning versus evening

### Tobin Silver, Anya Ellerbroek, Richard Buehn, Leo Vargas, Armando Tamayo, Corey Peacock, Jose Antonio

#### Nova Southeastern University, Exercise and Sport Science Lab, Davie Florida USA

##### **Correspondence:** Jose Antonio PhD (ja839@nova.edu) – Nova Southeastern University, Exercise and Sport Science Lab, Davie Florida USA

**Background**

Protein supplementation in trained men and women has been shown to improve body composition in conjunction with alterations in their heavy resistance-training program. Casein, a slow protein derived from milk, has been posited to have beneficial effects when consumed prior to sleep because it provides a slow and steady influx of amino acids for several hours. However, it is not known if it is the mere addition of extra protein or the timing of protein (i.e. consumption at night) that confers these effects. Thus, the purpose of this investigation was to determine the effects of casein supplementation (~54 grams) in the morning (Casein-MOR) or evening (Casein-EVE) (90 minutes or less prior to sleep) on measures of body composition and exercise performance in trained men and women.

**Methods**

Twenty-six healthy trained men and women completed this study (mean±SD; Casein-MOR group [n=14, seven male, seven female]: 30.0±8.2 yr; 170.7±9.5 cm; 70.9±13.9 kg. Casein-EVE group [n=12, nine male, three female]: 28.9±9.5 yr; 172.9±7.3 cm; 72.6±10.9 kg). Subjects in each group supplemented with casein protein (~54 grams) either in the morning (prior to 12 noon) or evening (~90 minutes or less prior to sleep). Subjects were advised to not significantly alter their training program. They were instructed to log their workouts. Volume load as well as minutes of aerobic exercise was determined. Body composition was assessed via the Bod Pod®. Body mass, fat mass, fat-free mass (FFM) and percent body fat was ascertained. In addition, subjects provided dietary self-reports via MyFitnessPal®. Approximately 24 daily dietary self-reports were provided from each subject that self-monitored their diet. An ANOVA was utilized to examine changes in body composition and performance.

**Results**

The data for body composition are shown in Table 2. The data for nutritional intake are shown in Table 3. Both groups consumed significantly more protein post versus pre with no differences between groups. There were no significant differences in the volume of aerobic or resistance training between groups (data not shown).

**Conclusions**

In trained subjects who did not significantly alter their training program, the addition of 54 grams of casein protein in the morning or evening had no significant effects on body composition. Furthermore, the additional consumption of protein calories does not result in an increase in fat mass despite the fact that exercise volume did not change.

**Acknowledgements**

The casein protein was provided by MusclePharm®.

**Table 2 (abstract P27):** Body Composition

	Casein-MOR		Casein-EVE	
	Pre	Post	Pre	Post
Weight kg	70.90±13.87	71.22±13.08	72.62±10.85	73.68±9.94
Fat Mass kg	12.63±5.80	12.59±5.52	11.57±3.90	11.42±4.21
FFM kg	58.20±11.33	58.63±11.26	61.06±10.88	62.26±11.00
% Body Fat	17.54±6.68	17.51±6.74	16.22±5.78	15.87±6.53

Data are mean±SD. There were no significant differences within or between groups

**Table 3 (abstract P27):** Dietary Intake

	Casein-MOR		Casein-EVE	
	Pre	Post	Pre	Post
Kcal	1859±683	1975±658	2076±571	2158±552
CHO g	193±77	177±66	204±67	186±61
PRO g	119±47	167±49	142±54	173±46
Fat g	68±28	67±30	77±29	80±27
Kcal/kg/d	27±9	28±8	29±8	30±8
CHO g/kg/d	2.8±1.1	2.6±1.0	2.9±1.0	2.6±0.9
PRO g/kg/d	1.7±0.6	2.4±0.6*	1.9±0.6	2.4±0.5*
Fat g/kg/d	1.0±0.3	0.9±0.3	1.1±0.4	1.1±0.4

Data are mean±SD. *Significant difference (pre vs post, p < 0.05). There were no between group differences

## P28 A high protein diet has no harmful effects: a one-year crossover study in resistance-trained males

### Anya Ellerbroek, Tobin Silver, Richard Buehn, Leo Vargas, Armando Tamayo, Corey Peacock, Jose Antonio

#### Nova Southeastern University, Exercise and Sport Science Lab, Davie Florida USA

##### **Correspondence:** Jose Antonio PhD (ja839@nova.edu) – Nova Southeastern University, Exercise and Sport Science Lab, Davie Florida USA

**Background**

Protein supplementation in trained men and women has been shown to improve body composition in conjunction with alterations in their heavy resistance-training program. However, there are claims that consuming a high protein diet may have deleterious effects on various parameters of health. Thus, the purpose of this investigation was to determine the effects of a high protein diet over a one-year period.

**Methods**

Eleven healthy resistance-trained men completed the study (mean±SD. Age 25.8±4.0 yr; Height 179.1±9.0 cm; Average years of training 8.9±3.4 yr). In a randomized order, subjects consumed their habitual diet for 2 months and 4 months as well as a higher protein diet for 2 months and 4 months. Thus on average, each subject was on their habitual (i.e., normal) diet for 6 months and a higher protein diet for 6 months. Each subject was provided with protein powder so that they could attain their protein intake goals. No other instructions were given in terms of dietary alterations. Subjects were instructed to train as they normally would. They logged their resistance training workouts. Body composition was assessed via the Bod Pod®. Body mass, fat mass, fat-free mass (FFM) and percent body fat was ascertained. In addition, subjects provided dietary self-reports via MyFitnessPal®. Each subject provided approximately 100-168 daily dietary self-reports. A paired t-test was used to examine the differences between the normal and high protein intakes.

**Results**

The data for body composition are shown in Table 4. The data for nutritional intake are shown in Table 5. Subjects consumed more total calories and protein during the High protein phase. There were no differences between the Normal and High groups in any measure of health (Table 6).

**Conclusions**

In well-trained male subjects, consuming a diet high in protein had no harmful effects on any measures of health. Furthermore, there was no change in body weight, fat mass or lean body mass despite eating more total calories and protein. Contrary to popular belief, the consumption of a high protein diet is not mutually exclusive with a diet high in fiber. This is the first one-year longitudinal investigation in well-trained males that demonstrates the safety of a high protein diet.

**Table 4 (abstract P28):** Body Composition

	Normal	High
Weight kg	70.90±13.87	71.22±13.08
Fat Mass kg	12.63±5.80	12.59±5.52
FFM kg	58.20±11.33	58.63±11.26
% Body Fat	17.54±6.68	17.51±6.74

Data are mean±SD. There were no significant differences between groups

**Table 5 (abstract P28):** Dietary Intake

	Normal	High
Kcal	2515±410	2891±384*
CHO g	236±61	244±42
PRO g	197±34	267±51*
Fat g	87±22	94±23
Kcal/kg/d	30.01±5.38	33.93±4.12*
CHO g/kg/d	2.84±0.82	2.89±0.60
PRO g/kg/d	2.32±0.36	3.12±0.48*
Fat g/kg/d	1.04±0.27	1.10±0.25
Fiber g	28±14	32±14

Data are mean±SD. *Significant difference (Normal vs. High, p < 0.05)

**Table 6 (abstract P28):** Select Clinical Measures – Cardiovascular, Renal, Hepatic

	Normal	High
Glucose mg/dL	87±15	86±10
BUN mg/dL	22±5	22±4
Creatinine mg/dL	1.1±0.1	1.1±0.2
eGFR	98±17	95±16
AST U/L	27±7	30±14
ALT U/L	24±6	28±12
Total Chol mg/dL	144±21	152±25
HDL-C mg/dL	50±11	48±10
TG mg/dL	62±27	64±18
LDL-C mg/dL	81±16	92±23

Data are mean±SD. There were no significant differences between groups

## P29 SUP (Stand-up Paddling) athletes: nutritional intake and body composition

### Adam Pollock^2^, Anya Ellerbroek^1^, Tobin Silver^1^, Corey Peacock^1^, Jose Antonio^1^

#### ^1^Nova Southeastern University, Exercise and Sport Science Lab, Davie Florida USA; ^2^Kokopelli's Gym Inc. Casselberry, Florida USA

##### **Correspondence:** Jose Antonio PhD (ja839@nova.edu) – Nova Southeastern University, Exercise and Sport Science Lab, Davie Florida USA

**Background**

According to the 5^th^ Annual “Special Report on Paddlesports,” an estimated 21.7 million Americans (~7.4 % of the American population) have participated in the paddle sports (www.outdoorfoundation.org). Of that figure, 2.8 million were from stand-up paddling (SUP). Despite the millions of participants in SUP, there is a no data on this group of athletes. Thus, the purpose of this investigation was to ascertain the dietary habits and physical characteristics of highly competitive SUP athletes in the state of Florida.

**Methods**

Eighteen SUP athletes volunteered for this study. Body composition was assessed via the Bod Pod®. Body mass, fat mass, fat-free mass (FFM) and percent body fat was ascertained. A smaller subset of subjects also performed a treadmill VO_2_max test to determine aerobic power. In addition, subjects provided dietary self-reports via MyFitnessPal®. Approximately 24 daily dietary self-reports were provided from each subject that self-monitored their diet.

**Results**

The data for body composition are presented in Table 7. The data for nutritional intake are presented in Table 8 (i.e., 11 of the 18 paddlers provided sufficient dietary data). All data are shown as the mean±SD.

**Conclusions**

Female SUP athletes are on average leaner than college-age female athletes in basketball, softball and crew. Male SUP athletes have a similar body composition to college-age basketball, hockey and football players. The dietary habits of SUP men and women differ in that men tend to consume more kcals, carbohydrate, fat and protein per kg body weight. Overall, one would describe male SUP athletes as having a ‘high protein’ diet whereas females tend to consume a moderate amount. (Reference: Ode JJ et al. Body mass index as a predictor of percent fat in college age athletes and nonathletes. MSSE. 39(3):403-409, 2007).

**Table 7 (abstract P29):** Body Composition of Competitive Male and Female Stand-Up Paddlers

	Female (n=9)	Male (n=9)
Age years	33.2±7.1	40.3±8.8
Height cm	163.5±4.8	175.8±2.7
Weight kg	60.05±6.76	78.10±8.59
Fat Mass kg	11.69±4.27	10.83±5.90
Fat Free Mass kg	48.31±4.19	67.29±7.25
% Body Fat	19.10±5.83	13.62±6.37
VO_2_max ml/kg/min^a^	50.50±4.95	57.00±9.00

Data are mean±SD

There was a statistically significant difference (P<0.05) between females and males for all parameters tested except VO2max (likely due to the small sample)

^a^n=2 for female and n=3 for male

**Table 8 (abstract P29):** Dietary Intake

	Female (n=5)	Male (n=6)
Kcal	1586±249	3127±903
CHO g	171±45	306±89
PRO g	102±16	220±105
Fat g	55±14	114±39
Kcal/kg/d	26.1±4.3	40.7±11.7
CHO g/kg/d	2.5±1.1	4.0±1.2
PRO g/kg/d	1.7±0.5	2.8±1.2
Fat g/kg/d	0.8±0.3	1.5±0.6
Cholesterol mg/d	243±87	439±215
Sodium mg/d	1893±592	2752±849
Sugars g/d	66±16	111±33
Fiber g/d	19±4	38±19

Data are mean±SD. All values were significantly different between males and females (p <0.05)

## P30 The effects of 8 weeks of colostrum and bio-active peptide supplementation on body composition in recreational male weight lifters

### A. Kreutzer^1^, P. Zavala^2^, S. Fleming^2^, M. Jones^3^, J. M. Oliver^1^, A. Jagim^2^

#### ^1^Exercise & Sport Performance Laboratory, Kinesiology Department, Texas Christian University, Fort Worth, TX, USA; ^2^ Exercise & Sport Science Department, University of Wisconsin – La Crosse, La Crosse, WI, USA; ^3^ Division of Health and Human Performance, George Mason University, Fairfax, VA, USA

##### **Correspondence:** A. Kreutzer (a.kreutzer@tcu.edu) – Exercise & Sport Performance Laboratory, Kinesiology Department, Texas Christian University, Fort Worth, TX, USA

**Background**

A commercially available bio-active peptide product (Bio-Gro™) as well as bovine colostrum have previously been shown to increase fat-free mass gains when used in conjunction with a structured training program. To our knowledge, no study has compared the efficacy of these supplements in augmenting fat-free mass gains during a program designed to elicit hypertrophy. Therefore, the purpose of this study was to compare the effects of bio-active peptides and bovine colostrum in combination with hypertrophy-specific resistance training on body composition in recreationally trained men.

**Methods**

Thirty-two resistance trained men (20±1 y; 179.9±5.9 cm; 78.4±6.1 kg) participated in a double-blind, placebo controlled design to determine the effects of bio-active peptides and colostrum on body composition changes induced by a hypertrophic strength training protocol. Body mass (BM), fat-free mass (FFM), fat mass (FM), and percent body fat (PBF) were assessed before and after training using air displacement plethysmography (*BODPOD, Cosmed, USA*). Participants were matched for FFM following baseline testing, and randomly assigned to ingest Bio-Gro™ (*Isatori, USA* [BIO]; n = 12), colostrum (*Isatori, USA* [COL]; n = 9) or Placebo (PLA; n = 11). All groups completed an 8-week resistance training program consisting of a 4-day split designed to elicit muscle hypertrophy. In addition, each participant was provided instructions on total energy and macronutrient content to facilitate increases in FFM.

**Results**

BM increased significantly (p < 0.001) in all three groups (PLA: 1.6±1.8 kg; COL: 1.9±2.1 kg; BIO: 1.7±1.9 kg), with no Group*Time interactions present (p = 0.925). FFM increase was greater (p = 0.008) in COL (2.5±1.0 kg) when compared with PLA (0.8±1.3 kg), and trended toward being greater (p = 0.090) in COL when compared with BIO (1.5±1.6 kg). There was no difference in FFM gain between BIO and PLA (p = 0.233). FM increased in PLA (+0.9±1.1 kg; p = 0.044), did not change significantly in BIO (0.5±1.8 kg; p = 0.228), and trended toward a decrease in COL (-0.9±1.2 kg; p = 0.071). Participants in the PLA group experienced a PBF gain of 1.0±1.3 % (p = 0.058), while PBF decreased by 1.4±1.2 % (p < 0.05) in those supplementing with COL. No change (p = 0.464) in PBF was found in participants in the BIO group.

**Conclusion**

The addition of bovine colostrum to a resistance program designed to elicit hypertrophy appears to augment FFM gains to a greater degree when compared with a commercially available bio-active peptide supplement. Furthermore, bovine colostrum seems to allow decreases in fat mass and percent body fat concurrent with increases in FFM. However, further study is warranted in a larger sample size to elucidate differences in the effects of colostrum and bio-active peptide supplements on the body composition of resistance trained individuals engaging in a strength training program designed to elicit hypertrophy.

## P31 Effects of a Popular Women’s Thermogenic Supplement During an Energy-Restricted High Protein Diet on Changes in Body Composition and Clinical Safety Markers

### Cody T. Haun^1^, Petey W. Mumford^1^, Parker N. Hyde^2^, Ciaran M. Fairman^2^, Wesley C. Kephart^1^, Darren T. Beck^3,1^, Jordan R. Moon^4^, Michael D. Roberts^1,3^, Kristina L. Kendall^5^, and Kaelin C. Young^3,1^

#### ^1^School of Kinesiology, Auburn University, Auburn, AL, USA; ^2^Department of Human Sciences, Ohio State University, Columbus, OH, USA; ^3^Edward Via College of Osteopathic Medicine – Auburn Campus, Auburn, AL, USA; ^4^School of Health sciences, American Public University System, Charles Town, WV, USA; ^5^Bodybuilding.com, Boise, ID, USA

##### **Correspondence:** Cody T. Haun (cth0023@auburn.edu) – School of Kinesiology, Auburn University, Auburn, AL, USA

**Background**

The increasing interest in weight loss has coincided with a rise in the supplemental use of energy drinks and thermogenics to aid weight loss efforts. To date, the effectiveness and safety of supplemental, proprietary blend thermogenics has not been thoroughly evaluated; specifically, their more long-term effects on weight-loss and clinical safety markers are yet to be elucidated. The purpose of this study was to investigate the safety and efficacy of a calorie-restricted, high-protein diet, with and without the use of a proprietary blend thermogenic on body weight and body composition changes in college-aged females.

**Method**

Thirty-nine college-aged females who were regular exercisers completed this randomized, double-blind, placebo-controlled study. Subjects were divided into three groups: Bizzy Diet (~1000 kcal, 40%PRO, 20 % CHO, 40 % FAT) + FitMiss Burn^TM^ (BURN, N=12), Bizzy Diet + Placebo (PLA, N=13) or Control (CON, N=14) and underwent two testing sessions separated by approximately 3 weeks. Resting blood pressure (BP), resting heart rate (RHR), clinical serum safety markers, body weight (BW), and body composition were assessed at baseline and after the 3 week intervention. Repeated measures ANOVA was used to asses group x time interaction effects with an alpha level of ≤ 0.05. Follow up analyses included Bonferonni post-hoc analyses as well as dependent t-tests for within group differences.

**Results**

Repeated measures ANOVA revealed a significant interaction effect for both BW (p<0.01) and total fat mass (FM, p=0.005). Post-hoc analysis revealed the BURN and PLA groups experienced significant decreases in both BW (Pre vs. Post; BURN: 68.8±8.3 vs 66.1±8.5, p<0.01; PLA: 66.7±8.8 vs 64.7±8.6, p<0.01; CON: 67.2±9.6 vs 67.2±9.6 kg) and FM (BURN: 19.0±4.5 vs 17.1±4.0, p<0.001; PLA: 18.0±3.2 vs 16.3±2.9, p<0.001; CON: 19.2±5.6 vs 18.4±5.7 kg) compared to CON, with no difference between BURN and PLA. There were no significant group x time interactions for BP, RHR, or clinical safety markers over the course of the intervention.

**Conclusion**

The Bizzy diet, both with and without the addition of FitMiss Burn^TM^ thermogenic, appears to be safe for short-term use. However, consuming FitMiss Burn does not appear to further improve BW or body fat loss during a high-protein energy-restricted diet.

**Acknowledgements**

This study was supported by funds provided from MusclePharm Inc.

## P32 Three days of caffeine consumption following caffeine withdrawal yields small strength increase in knee flexors

### Geoffrey M Hudson^1^, Tara Hannings^2^, Kyle Sprow^1^, Loretta DiPietro^1^

#### ^1^The George Washington University, Washington, D.C., USA; ^2^La Salle University, Philadelphia, PA., USA

##### **Correspondence:** Geoffrey Hudson (ghudson2@gwu.edu) – The George Washington University, Washington, D.C., USA

**Background**

Acute, high doses of caffeine have demonstrated ergogenic effects in aerobic and anaerobic activities. However, it is unclear how daily caffeine reintroduction can influence strength and power following caffeine withdrawal. The purpose of this investigation was to determine the effects of caffeine consumption on peak torque (PT), average power, total work, and work fatigue during exercise with an isokinetic dynamometer following a caffeine withdrawal period.

**Methods**

Physically active, moderate to high level caffeine consumers (n=25; 21 female, 4 male; age: 22±3; mass: 62.14±8.26 kg; average reported daily caffeine consumption: 255.8±126.3 mg) performed anaerobic exercise tests following 4 days of caffeine withdrawal (T1) and then 3 days of 5 mg-kg^-1^ caffeine reintroduction (T2) Isokinetic PT, average power, and total work were tested in the subjects’ dominant leg at 60°·s^-1^, 180°·s^-1^, and 300°·s^-1^. Short duration endurance was assessed in 30 repetitions at 180°·s^-1^. Isometric PT was measured at 30° and 90° flexion. Data were analyzed with paired t-tests, an alpha of 0.05, and presented as means ± SD.

**Results**

Following caffeine reintroduction, knee flexion PT at 180°·s^-1^ increased by 3 N-m (49±17 N-m vs. 53±19; p=0.017); average PT at 180°·s^-1^ increased from 44±17 N-m to 46±17 N-m (p=0.04); and PT at 300°·s^-1^ increased by 3 N-m (44±16 N-m vs. 48±18; p=0.03). No changes were seen in knee extension PT at 60°·s^-1^, 180°·s^-1^, or 300°·s^-1^ from T1 to T2 (119±40 N-m to 119±36, 94±33 N-m to 94±28, and 72±26 N-m to 73±26). During 30 repetitions at 180°·s^-1^, flexion average PT saw a non-significant increase from T1 to T2 (39±13 N-m to 41±13; p=0.118). No significant differences in average power were seen in any of the tests. No significant changes were seen in isometric PT at 30° of knee flexion or at 90° of flexion for knee extension (71±21 N-m to 70±26; 127±55 N-m to 129±53) or knee flexion (66±29 N-m to 67±29; 59±24 N-m to 58±24) from T1 to T2.

**Conclusions**

The current study demonstrates that reintroduction of caffeine following caffeine withdrawal increased isokinetic torque in only a few of the measured variables. This is contrary to other data from this intervention that previously demonstrated that caffeine withdrawal resulted in decreased torque and power production.

## P33 Comparison of cellular nitric oxide production from various sports nutrition ingredients

### Doug Kalman^1^, Sara Perez Ojalvo^2^, James Komorowski^2^

#### ^1^Metavantage Sciences, Inc, Weston, FL, USA; ^2^Nutrition 21, LLC, Purchase, NY, USA

##### **Correspondence:** Doug Kalman (dougkalman@gmail.com) – Metavantage Sciences, Inc, Weston, FL, USA

**Background**

The purpose of this *in vitro* study was to evaluate the ability of various sports nutrition compounds, including inositol-stabilized arginine silicate (ASI; Nitrosigine®), to stimulate cellular production of nitric oxide (NO), a key factor in increasing vasodilation and the flow of blood and oxygen to skeletal muscles. Increasing NO is important for athletes, including bodybuilders and exercise enthusiasts who wish to improve their workouts and have faster recovery, as fueling NO production can lead to increased blood flow and delivery of oxygen and nutrients to muscles during and after a workout. Arginine is a NO precursor. As arginine becomes depleted throughout a workout, arginine supplements and other sports nutrition compounds may help restore this process, resulting in increases in NO and blood flow and leading to fitness benefits. ASI has been previously shown to significantly enhance nitric oxide in a clinical study, as well as significantly increasing blood levels of arginine up to six hours post-dose while an equivalent dose of arginine, from Arginine HCl, did so for only one hour. This *in vitro* study was designed to compare the cellular production of NO of several sports nutrition ingredients including ASI, L-Arginine, L-Arginine AKG, L-Citrulline, L-Citrulline Malate and Agmatine Sulfate.

**Methods**

To measure cellular production of nitric oxide, RAW 264.7 macrophage cells were cultured overnight in 96-well microplates followed by treatment with control, ASI, and the other sports nutrition test ingredients in a stepwise fashion. ASI was dosed at a concentration of 1.0 g/L. Cell culture concentrations of the other compounds were dosed relative to a 1.5 g dose of ASI using the following doses: L-Arginine 1.5 g, L-Arginine AKG 4.0 g, L-Citrulline 3.0 g, L-Citrulline Malate 3.0 g and Agmatine Sulfate 1.0 g. As NO is unstable and rapidly converts to nitrites or nitrates, nitrite levels were measured using the Greiss Method and also analyzed by the Measure-iT Assay, after 24 hours of culture.

**Results**

**Fig. 3 (abstract P33):**
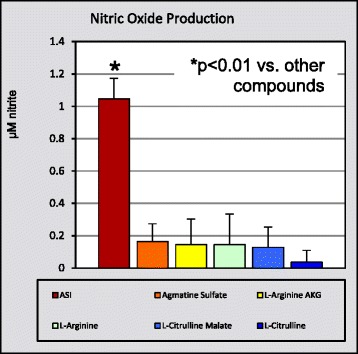
Nitrite Levels, as a marker of Nitric Oxide, of ASI and other sports nutrition compounds in an in vitro study

At the doses used in this study, ASI significantly increased NO production over each of the five other compounds tested (p<0.01; Fig. 3). There was a greater than 5X increase in NO production with ASI compared to the other tested sports nutrition ingredients. In addition, of the sports nutrition ingredients tested, only ASI significantly increased NO production versus control (p<0.01).

**Conclusions**

In this *in vitro* study to evaluate NO production of a number of sports nutrition ingredients using the established Greiss assay to detect cellular NO production, ASI significantly enhanced NO levels compared to the other compounds and also versus control. Increasing NO can lead to substantial benefits for training athletes and fitness enthusiasts as it can result in enhanced delivery of oxygen and nutrients to working muscles, positively affecting workouts and recovery. Further preclinical and clinical studies are recommended.

## P34 The effects of 8 weeks of bio-active peptide supplementation on training adaptations in recreational male weight lifters

### P. Zavala^1^, S. Fleming^1^, M. Jones^2^, J. Oliver^3^, A. Jagim^1^

#### ^1^Exercise & Sport Science Department, University of Wisconsin – La Crosse, La Crosse, WI, USA; ^2^Division of Health and Human Performance, George Mason University, Fairfax, VA, USA; ^3^Exercise and Sport Performance Laboratory, Kinesiology Department, Texas Christian University, Fort Worth, TX, USA

##### **Correspondence:** P. Zavala (zavala@uwlax.edu) – Exercise & Sport Science Department, University of Wisconsin – La Crosse, La Crosse, WI, USA

**Background**

Supplementation with bio-active peptides have been purported to enhance short-term improvements in strength development when combined with a structured training program by positively influencing recovery and work capacity. However, less is known regarding the benefits of long-term supplementation, and whether or not bio-active peptides are superior to a similar ergogenic aid, whole colostrum, in regards to their ability to enhance training adaptations. Therefore the purpose of this study was to examine the effects of bio-active peptide supplementation and colostrum in conjunction with an 8 week strength training program on measures of strength and power in recreational male weight lifters.

**Methods**

Thirty-two recreationally trained male weight lifters (20±1 y; 179.9±5.9 cm; 78.4±6.1 kg) participated in a double blind, placebo controlled design to determine the effects of bio-active peptides on training adaptations in recreational weightlifters. Before and following an 8-week strength training program consisting of a 4-day split designed to increase muscle hypertrophy upper and lower body strength were assessed by a 3 RM bench press (3RM_BP_) and 3RM back squat (3RM_BS_) test, respectively. Participants also completed a counter-movement vertical jump (CMVJ) which was later converted to Watts (W) to determine lower body power (*Sports Imports, Columbus, OH*). Following baseline measures, participants were randomly assigned to ingest Bio-Gro^TM^ (*Isatori, USA* [SUP]; n = 12), colostrum (COL; n =9) or Placebo (PLA; n = 11). In addition, each participant was provided instructions on total energy and macronutrient content to facilitate increases in fat-free mass. A two-way (group x time) repeated measures analysis of variance was used to examine changes in strength and power between groups. Data are presented as mean ±SD change from baseline. Statistical significance was determined at the p < 0.05 level.

**Results**

Upper (PLA, 6.27 ± 1.85 kg; BIO, 6.96 ±1.94 kg; COL, 10.87 ± 2.32 kg) and lower body (PLA, 14.13 ±2.83 kg; BIO, 17.21 ± 2.97 kg; COL, 15.60 ± 3.54 kg) strength increased in all groups as evidenced by a time effect in 3RM_BS_ (p < 0.001) and 3RM_BP_ (p < 0.001). However, no interaction effect was observed in either 3RM_BS_ (p = 0.757) or 3RM_BP_ (p = 0.290). Similarly, average power as measured by CMVJ also improved in all groups (PLA, 180 ± 49 W; BIO, 276 ± 52 W; COL, 215 ± 62 W), but no interaction was observed (p = 0.413).

**Conclusion**

The results of the current study indicate that the addition of bio-active peptides to a strength training regimen appears to offer minor advantages in the development of upper body strength and lower body power; however these changes were not statically different amongst groups. Further, colostrum supplementation appears to augment improvements in upper body strength compared to placebo and bio-active peptide supplementation albeit not statistically significant. Larger samples are likely needed to determine the long-term benefit of bio-active peptide supplementation and whether or not it is superior to colostrum as an ergogenic aid. Additionally, differences in training adaptations may be more evident in an untrained population.

## P35 Effects of MusclePharm Assault Black^TM^ on lower extremity spinal excitability and postactivation potentiation: A pilot study

### Brian Wallace, Haley Bergstrom, Kelly Wallace

#### Department of Kinesiology and Health Promotion, University of Kentucky, Lexington, KY 40506, USA

##### **Correspondence:** Brian Wallace (brian.wallace@uky.edu; brian.wallace@northern.edu) – Department of Kinesiology and Health Promotion, University of Kentucky, Lexington, KY 40506, USA

**Background**

Caffeine has been shown to increase muscular strength, power, and endurance, and is the main active ingredient in the dietary supplement Assault Black^TM^ (MusclePharm, Denver, CO, USA). Muscle performance can be enhanced via post-activation potentiation (PAP). PAP is achieved through the modulation of neuromuscular function via increased spinal excitability of motor units as measured by the Hoffmann Reflex (Hmax/Mmax ratio), or from increased calcium sensitivity as a result of myosin light chain phosphorylation (Mmax force), following a conditioning activity such as heavy resistance exercise or a maximum voluntary isometric contraction (MVIC). The purpose of this study was to investigate the effect of Assault Black^TM^ on PAP of the soleus.

**Methods**

Five men and three women (26.5 ± 6.4 years, 173.5 ± 13.9 cm, 79.9 ± 20.7 kg) with at least one year of continuous strength training experience volunteered. Men and women were required to back squat at least 1.5 and 1.0 times bodyweight, respectively. A randomized crossover design was utilized, where subjects received either the supplement or a placebo on alternating visits. Men received 1.5 servings and women received 1 serving of the supplement. Each serving contained 300 mg caffeine. Twenty minutes after consumption of the beverage subjects were positioned on a Biodex System 4 isokinetic dynamometer (Biodex Medical Systems, Shirley, NY) with standardized positioning. Their foot was attached to the foot plate where plantarflexion force was recorded. A Hmax-Mmax stimulus response curve was obtained by delivering 1 ms duration twitches of increasing intensity to the soleus via square-wave impulses sent to the tibial nerve (Model DS7AH, Digitimer, Ltd., UK). Electromyography (EMG) recorded action potentials of the soleus (Bagnoli-8, Delsys, Inc., Boston, MA). Electrode placements were marked to ensure consistent placement for the subsequent visit. Ten minutes after the stimulus response curve was obtained, subjects performed a 10 second MVIC plantarflexion conditioning activity. Ten seconds thereafter, they received a stimulation at the intensity which elicited a maximum amplitude M-wave (Mmax), +20 %, followed ten seconds thereafter by a twitch at the intensity that elicited a maximum H-wave (Hmax). The H/M ratio, force in response to the Mmax and Hmax twitches, and MVIC peak force were analyzed in SPSS (version 22) using paired t-tests. The level of significance was p<0.05.

**Results**

The supplement significantly increased PAP as measured by both increased spinal excitability (mean difference: 10.29 ± 10.91 %, p=0.031) and increased muscular calcium sensitivity (2.30 ± 2.74 Nm, p=0.049). The net PAP response to the conditioning activity, which is a combination of the neural and muscular contributions measured by force at Hmax, was not different (-0.73 ± 3.78 Nm, p=.0600). Subjects’ peak MVIC force was equal between conditions (p=0.226).

**Conclusions**

Ingesting Assault Black^TM^ lead to significantly greater spinal excitability and phosphorylation induced PAP, but not net PAP. After a conditioning activity spinal excitability has been shown to be moderated for several minutes before increasing. A longer period of time may be necessary for net potentiation to become apparent.

**Acknowledgement**

This study was funded by MusclePharm through the ISSN Unrestricted Educational Grant.

## P36 Effects of four weeks of Ketogenic Diet alone and combined with High intensity Interval Training or Continuous-Moderate intensity on body composition, lipid profile and physical performance on healthy males

### Matias Monsalves-Alvarez^1,2,3^, Sebastian Oyharçabal^1^, Victoria Espinoza^4^

#### ^1^Institute of Nutrition and Food Technology, University of Chile, Santiago, Chile; ^2^Exercise Physiology Laboratory, Department of Biological Sciences, Faculty of Biological Sciences, Universidad Andres Bello, Santiago de Chile; ^3^School of Kinesiology, Health Research Centre, Faculty of Medicine, Finis Terrae University, Santiago, Chile; ^4^Center of Biomedical and Applied Research, Medicine School, Faculty of Medicinal Sciences, University of Santiago de Chile USACH, Santiago, Chile

##### **Correspondence:** Matias Monsalves-Alvarez (matias.monsalves@inta.uchile.cl) – Institute of Nutrition and Food Technology, University of Chile, Santiago, Chile

**Background**

Ketogenic diets are gaining rapid popularity among sedentary, active people and by some athletes due to their rapid effects on body composition. These changes are caused by a stimulation of lipolysis, derived from the shortage of dietary carbohydrates. Exercise is also a useful tool recommended for weight loss. High Intensity Interval training (HIIT) and moderate intensity are the modalities that induce the most significant fat reduction, whichever the population trained. The purpose of this study is to evaluate if High intensity Interval Training (HIIT) has a greater impact on body composition parameters, lipid profile and physical performance than continuous moderate training, both modalities under ketogenic diet for one month.

**Methods**

Sixteen healthy males where recruited for a one-month intervention. At baseline participants were assessed to calculated VO_2peak,_ Resting Metabolic Rate, Respiratory Quotient (Qr), Anaerobic Threshold, Body Composition assessment by DEXA. A blood sample was obtained to determine total Cholesterol, HDL, LDL, triglycerides, SGOT and GPT. All assessments were repeated at the end of the study. Participants were randomly assigned to one of three groups. High Intensity Interval Training-Ketogenic Diet (n=6, HIIT-KD), where training consisted in 10x1 minute sprints at ~90%HR_max_ and 1 minute of recovery 3 times per week. Moderate-Continuous Exercise-Ketogenic-Diet (n=6, MOD-KD), who were trained during 30 minutes at 60-65%VO_2max,_ 3 times per week in a braked cycle ergometer. Ketogenic Diet alone (n=6, KD) who maintained their normal activities. The KD provided 75 % fat, 20 % protein and 5 % carbohydrates for all groups. Adherence to the diet was monitored with weekly measurements of capillary Beta-Hydroxybutyrate (BHB).

**Results**

At the end of the intervention there was a reduction of total fat mass in all groups (KD p=0.0021, MOD-KD p=0.0574 and HIIT-KD p= 0.0034). Trunk fat decreased significantly among HIIT-KD (p=0.0055) participants but not in the MOD-KD (p=0.0738) group. Muscle mass was unchanged in all three groups. BHB levels where higher at week 2 on HIIT-KD than in MOD-KD (p >0.0001). On physical performance, VO2_peak_ increased significantly on HIIT-KD (p=0.0082) but not in MOD-HIIT (p=0.0688) participants. Power output increased only in HIIT-KD (p=0.0236). KD group had an important decrease on the anaerobic threshold (%VO_2max_) (p=0.0139). The respiratory quotient declined significantly only in HIIT-KD (p=0.0076). Only the KD participants had significant changes in lipid profile (LDL increase and TG decrease p=0.0281). No changes on SGOT and GPT where observed in any of the groups after four weeks on ketogenic diet.

**Conclusion**

We conclude that HIIT-KD lead to greater total fat mass loss, VO_2peak_ and power output increments compared with MOD-KD with no impairments on lipid and hepatic markers. The KD alone leads also to a reduction in fat mass loss without changes muscle mass. However it hampers exercise capacity when is performed by itself.

## P37 Effect of branched-chain amino acid supplementation on creatine kinase, muscular performance, and perceived muscle soreness following acute eccentric exercise

### Trisha A. VanDusseldorp^1^, Kurt A. Escobar^1^, Kelly E. Johnson^1^, Nathan Cole^1^, Terence Moriarty^1^, Matthew Stratton^1^, Marvin R. Endito^1^, Christine M. Mermier^1^, Chad M. Kerksick^2^

#### ^1^Department of Health, Exercise, and Sport Sciences, University of New Mexico, Albuquerque, NM, USA; ^2^Department of Sport, Recreation, and Exercise Sciences, Lindenwood University, St. Charles, MO, USA

##### **Correspondence:** Trisha A. VanDusseldorp (tvandusseldorp@unm.edu) – Department of Health, Exercise, and Sport Sciences, University of New Mexico, Albuquerque, NM, USA

**Background**

The purpose of this study was to examine the effect of branched-chain amino acid (BCAA) supplementation on markers of muscular recovery following muscle-damaging eccentric exercise.

**Methods**

Twenty resistance-trained males (age 22±3 yr, height 175.5±6.9 cm, and body mass 91.6±15.4 kg) were randomly assigned to a supplement (n=10) or placebo (PLA) (n=10) group. Subjects consumed either a BCAA supplement (.1 g/lb body weight) or PLA for eight days total, with a four day loading period prior to a muscle damaging exercise bout. During the eight day protocol, subjects adhered to a diet consisting of 1.2 g/kg/d protein as administered by a registered dietician. On day five, the damaging exercise protocol was performed which consisted of ten sets of eight repetitions of four-second eccentric squats at 70 % one repetition maximum (1RM). Immediately following squat performance, subjects completed 5 sets of 20 split squat jumps (10 each leg). Plasma creatine kinase (CK), vertical jump (VJ), maximal voluntary contraction (MVC), jump squat (40 % 1RM), and perceived muscle soreness (DOMS; 0 – 10 cm scale) were measured as indirect markers of muscle damage. All variables were measured immediately before the exercise protocol, as well as at 1, 2, 4, 24, 48, and 72 hours (hrs) post-exercise.

**Results**

Plasma CK concentrations were significantly elevated above baseline (p<0.001) in both BCAA and PLA groups at 4, 24, 48, and 72 hrs post-exercise. While no significant group-by-time effect was detected for plasma CK (p=0.10), plasma CK levels were significantly lower for the BCAA group at 48 hrs post-exercise (p=0.02; BCAA: 799.2±197.6; PLA: 1422.9±630.8 IU/L). Perceived muscle soreness increased from baseline (p<0.01) in both groups at 1, 2, 4, 24, 48, and 72 hrs, however the BCAA group reported significantly less soreness (p<0.01) at 48 (BCAA: 4.59±1.42; PLA: 7.14±1.65 cm) and 72 hrs post-exercise (BCAA: 1.38±1.83; PLA: 3.90±1.52 cm). MVC measures were significantly higher for the BCAA group at 24 h post-exercise (p=0.04; BCAA: 270.8±68.8; PLA: 210.9±38.5 Nm), but no significant group-by-time effect was observed (p=0.18). No significant difference between groups (p>0.05) was detected for VJ or jump squat performance.

**Conclusions**

BCAA supplementation (.1 g/lb body weight) may mitigate perceived muscle soreness following muscle damaging eccentric exercise; however, when consumed amid a diet adequate in daily protein (1.2 g/kg/d), the apparent attenuation of muscular performance decrements or corresponding plasma CK levels seems modest.

## P38 Effects of endurance training on markers of ribosome biogenesis in rodents fed a high fat diet

### Matthew A. Romero^1^, C. Brooks Mobley^1^, Melissa Linden^2^, Grace Margaret-Eleanor Meers^2^, R. Scott Rector^2^, Michael D. Roberts^1^

#### ^1^School of Kinesiology, Auburn University, Auburn, AL, USA; ^2^Internal Medicine-Division of Gastroenterology and Hepatology/NEP, University of Missouri, Columbia, MO, USA

##### **Correspondence:** Michael D. Roberts, PhD (mdr0024@auburn.edu) – School of Kinesiology, Auburn University, Auburn, AL, USA

**Background**

Ribosome biogenesis (RiboBio), or the de novo synthesis of new ribosomes, is regulated in part by the mammalian target of rapamycin (mTOR) signaling pathway. Diet-induced diabetes abrogates mTOR signaling, albeit exercise training has been shown to reverse negative diabetic-induced skeletal muscle phenotypes. Hence, the purpose of the current study was two-fold: 1) examine how diet-induced diabetes affects markers of RiboBio, and 2) examine if exercise in the presence of a diabetic-inducing diet prevents potential decrements on markers of RiboBio.

**Methods**

Male Otsuka Long-Evans Tokushima Fatty (OLETF) rats were used in the current study. At 20-32 weeks of age rats were provided a standard chow diet (O-Con), a high-fat diet (O-HFC), or a high-fat diet coupled with endurance training (O-HFC/EX). O-HFC/EX were treadmill-trained 5d/wk. Speed and duration of the treadmill exercise were gradually increased over the first four weeks of training until the animals could maintain a running speed of 20 m/min for 60 min/d. At the end of the intervention, vastus lateralis muscles were harvested from each respective group for analysis of RiboBio markers.

**Results**

O-HFC rats exhibited a diabetic phenotype as evidenced by significant increases in hemoglobin A1c levels compared to O-Con rats, albeit this phenotype was diminished in O-HFC/EX rats. Total RNA, a surrogate of ribosome content, was 57 % lower in O-HFC/EX versus O-HFC rats (p=0.008). However, total myofibrillar protein to total RNA, a surrogate of translational efficiency, was 63 % greater in O-HFC/EX versus O-HFC rats (p=0.004). RNA polymerase I (Pol I) protein levels (which catalyzes rDNA transcription and RiboBio) were 77 % greater in O-HFC/EX versus O-HFC rats (p<0.01). O-HFC/EX rats also presented a greater expression in pre-45S rRNA versus O-Con and O-HFC groups, respectively (+65 and 93 %, p=0.001 and p=0.034). Upstream binding factor (UBF) (a transcriptional regulator of RiboBio) was 158 % greater in O-HFC/EX versus O-HFC rats (p=0.012). Given that ribosome content was lower in O-HFC/EX versus O-HFC rats despite other RiboBio markers being elevated in the former group we examined select ribophagy markers (i.e, USP10 and G3BP1 protein levels) with the expectation that exercise training increased ribosomal turnover. However, these markers were not elevated in O-HFC/EX rats versus other treatments.

**Conclusion**

Diet-induced diabetes, while appearing to increase skeletal muscle ribosome content, decreases markers of translational efficiency and RiboBio. Endurance training in the presence of a diabetes-inducing diet improves ribosome function (i.e., translational efficiency) and increases various markers of RiboBio, albeit the mechanism whereby ribosome content is lowered with endurance training warrants further investigation. Importantly, these data continue to outline how nutrition and exercise affect RiboBio, and future studies are needed in order to determine how select ‘anabolic’ nutritional supplements (i.e., dietary protein, amino acids, creatine, etc.) affect this aspect of skeletal muscle physiology.

## P39 The effects of acute citrulline-malate on lower-body isokinetic performance in recreationally active individuals

### Joshua L Gills^1^, Hocheng Lu^1^, Kimberly Parker^1^, Chris Dobbins^1^, Joshua N Guillory^1^, Braden Romer^2^, David Szymanski^1^, Jordan Glenn^3^

#### ^1^Louisiana Tech University, Ruston, LA, USA; ^2^High Point University, High Point, NC, USA; ^3^Omada Health, San Francisco, CA, USA

##### **Correspondence:** Joshua L Gills (jlg078@latech.edu) – Louisiana Tech University, Ruston, LA, USA

**Background**

Citrulline-Malate (CM) purportedly has an ergogenic benefit through nitric oxide production (NO), which may increase vasodilatory properties. A few studies have been completed examining CM supplementation on muscular endurance, but none potential gender effect of CM supplementation on muscular endurance. Therefore, the purpose of this investigation was to examine a potential gender effect of the acute effects of exogenous CM supplementation on lower-body isokinetic (ISO) muscular endurance test protocol.

**Methods**

21 recreationally active (*n* = 14 males, *n* = 7 females; Vo_2peak_ = 50.7 ± 8.7 ml•kg^-1^•min^-1^) subjects (19.1 ± 7.1 body fat %; 71.4 ± 13.5 kg; 170.5 ± 10.7 cm, 22.0 ± 2.9 years) completed randomized, double-blind trials consuming CM (12 g dextrose + 8 g CM) or a placebo (12 g dextrose). Prior to supplementation visits, subjects completed a Vo_2peak_ test and were familiarized to the cycling protocols on separate days. During the supplementation trials, participants performed a 50 repetition (muscular endurance) ISO protocol sixty minutes after consumption of the CM or placebo drink. A minimum of 1-week was required between trials. For each supplementation trial, females had to be on their menstrual cycle. No alcohol, caffeine, or vigorous exercise was permitted 24 hours before each trial. No food or drink intake was permitted up to 3 hours before each trial.

**Results**

During the 50 repetition ISO extension, females displayed better angle peak torque (*p* = .03), torque at 30 degrees (*p* = .01), total work (*p* = .04), and work during the 1^st^ third of the test (*p* = .05) while ingesting CM. Males had no reported significant effects during the 50 repetition ISO extension phase while consuming CM. Neither males nor females alone showed significant effects during the flexion phase while taking CM. However during the 50 repetition ISO flexion phase with males and females combined, the results revealed significant total work (*p* = .04) while consuming CM. And in the 50 repetition ISO extension phase, the work during the 1^st^ third of the test (*p* = .05) was significantly greater while taking CM with males and females data combined.

**Conclusion**

Females experienced an increased torque production and work with CM as compared to males. The estrogen levels of females reach a minimum during menstruation; therefore, as estrogen is a potent stimulator of nitric oxide synthase (NOS), CM may play a positive role in enhancing NOS production during menses.

## P40 The effect pre-ingested L-isoleucine and L-leucine on blood glucose responses and glycemic hormones in healthy inactive adults: Preliminary data

### Daniel E. Newmire^1^, Eric Rivas^2^, Sarah E. Deemer^1^, Robert Wildman^3^, Victor Ben-Ezra^1^

#### ^1^Exercise and Biochemistry Laboratory, Department of Kinesiology, Texas Woman’s University, Denton, TX, USA; ^2^Institute for Clinical and Translational Science & Department of Pediatrics, The University of California, Irvine, CA, USA; ^3^Dymatize Nutrition Sport Performance Institute, Dallas, TX, USA

##### **Correspondence:** Daniel E. Newmire (dnewmire@twu.edu) – Exercise and Biochemistry Laboratory, Department of Kinesiology, Texas Woman’s University, Denton, TX, USA

**Background**

The ingestion of whey protein or insulinogenic amino acids (AA) with a carbohydrate drink has been shown to blunt the elevated post-prandial glucose response. Though not fully elucidated, it has been suggested that AA may facilitate enteroendocrine hormones glucagon-like peptide-1 (GLP-1) and glucose-dependent insulinotropic peptide (GIP) that are 50-70 % responsible for regulating insulin secretion and inhibiting glucagon output. Therefore, the purpose of this study is to observe the “*priming*” effect of pre-ingested L-Isoleucine (ISO) and L-Leucine (LEU) on glucose metabolism and glycemic hormones in healthy inactive adults. Early data from this study was presented at the Texas ACSM Regional Conference 2016.

**Hypothesis**

We hypothesize that the pre-ingested ISO and LEU would diminish the post-prandial rise in glucose during a 75 g oral glucose tolerance test (OGTT) and have minimal effect on enteroendocrine hormone secretion.

**Methods**

To test this, 12 healthy adults (Females: n =6, Males: n=6, Age 27.39 ± 2.05 y; Height 167.42 ± 2.23 cm; Weight 77.77 ± 3.73 kg; BMI 26.30 ± 2.14 kg/m2; Lean body mass [LBM] 53.20 ± 4.67 kg; Body fat 34.14 ± 2.96 %; Fasting blood glucose [FBG] 89.5 ± 4.67 mg/dL) completed four trials in a randomized, single-blinded fashion. The four trials required participants to ingest either ISO + LEU in combination (1:1), ISO, LEU, and lastly a placebo (PLA). Each treatment was ingested 30 min prior to a 2 h 75 g OGTT. The amino acid drink (200 mL) was standardized by participant LBM (0.3 g/kg) while the control consisted of inert stevia and soy lecithin found in equal amounts as other treatment mixtures (3.54 g). Venous blood samples were taken at baseline (0), (amino acid drink ingestion), 10, 30, (75 g glucose drink ingestion), 40, 60, 90, 120, and 150 min. Plasma glucose (GLU) was analyzed using a YSI 2900 analyzer (Yellow Springs Instruments) and insulin (INS), C-peptide, glucagon (GCG), GIP_Total_, and GLP-1_Active_ concentrations were quantified by fluorescent bead-based multiplexing technology (MAGPIX, Luminex xMAP technology). A 2-way RMANOVA (Treatment x Time) was used to assess glucose and hormone data (GraphPad Prism Software).

**Results**

∆ glucose analysis (n=12), pre-ingestion ISO+LEU, ISO, and LEU blunted glucose response more so than PLA (*P*=0.005). ISO + LEU and ISO lower blood GLU at 60 and 90 min (*P*=<0.05) compared to PLA. Currently, only six individuals have been analyzed for INS, C-peptide, GCG, GLP-1_Active_, and GIP_Total_. At present, we have observed that pre-ingested LEU and ISO have minimal impact on glycemic hormones compared to PLA due to similar responses between treatments.

**Conclusion**

Based on these preliminary results, it appears that pre-ingestion of ISO and LEU combined or independently, at 0.3 g/kg/LBM diminish glucose responses more so than PLA in apparently healthy and inactive young adults.

## P41 Does protein and source impact substrate oxidation and energy expenditure during and after moderate intensity treadmill exercise?

### C Kerksick, B Gieske, R Stecker, C Smith, K Witherbee

#### School of Sport, Recreation and Exercise Sciences, Lindenwood University, St. Charles, MO, USA

##### **Correspondence:** C Kerksick (ckerksick@lindenwood.edu) – School of Sport, Recreation and Exercise Sciences, Lindenwood University, St. Charles, MO, USA

**Background**

Performing cardio while fasted is a popular strategy to stimulate greater fat burning, however, recent evidence indicates being fed may impact rates of fuel oxidation. Studies suggest that protein ingestion can trigger greater increases in energy expenditure and fat oxidation, but no studies to date have examined any impact of the source of protein on energy expenditure and fat oxidation during as well as post-exercise.

**Methods**

Eleven healthy, college-aged males (23.5 ± 2.1 years, 86.0 ± 15.6 kg, 184 ± 10.3 cm, 19.7 ± 4.4 %fat) completed four identical testing sessions in a randomized, counter-balanced, crossover fashion. All participants abstained from > 200 mg caffeine ingestion and other supplementation except for protein and multi-vitamins for 30 days prior to arriving for all testing after observing an overnight fast. Upon arrival participants completed a resting metabolic rate assessment using indirect calorimetry for determination of baseline substrate oxidation and energy expenditure. In a randomized, double-blind, crossover fashion, participants then ingested similar colored, isovolumetric (12 fl oz. cold water) solutions mixed with either whey isolate or casein protein powder delivering 25 g protein or an isocaloric maltodextrin powder or a non-caloric control condition. Participants then sat quietly for 30 minutes before completing a standardized warm-up protocol. Participants were then connected to a metabolic cart where they completed 30 minutes of treadmill exercise at 55 % heart rate reserve. Approximately 15 minutes after completing the exercise bout, study participants then completed a second resting metabolic rate assessment. All supplement ingestion, warm-up completion and exercise was directly supervised by a study investigator.

**Results**

4 × 2 mixed factorial ANOVA with repeated measures on test were completed to determine any significant main and interaction effects. Resting energy expenditure data was normalized to body mass (in kg) and a significant group x time interaction (p = 0.002) was found. Oneway ANOVA with the delta scores were found to be significant (p = 0.002) and post-hoc comparisons indicated that whey (3.41 ± 1.63 kcal/kg) was greater (p<0.05) than maltodextrin (1.57 ± 0.99 kcal/kg, p = 0.010) and tended to be greater than the non-feeding control group (2.00 ± 1.91 kcal/kg, p = 0.055). Casein (3.38 ± 0.82 kcal/kg) was greater than maltodextrin (p = 0.012) and tended to be greater than the non-feeding control group (p = 0.061). No significant group x time interaction effect (p = 0.116) was found for respiratory exchange ratio data, however, whey and casein were found to significantly decrease (p<0.05) during post exercise period while no change (p>0.05) was seen for maltodextrin or the non-feeding control group.

**Conclusions**

Consuming supplemental levels of protein or maltodextrin might not significantly impact fuel oxidation substrates ratio as commonly believed. Additionally, pre-exercise feeding with whey (15.9 ± 8.3 %) and casein (15.4 ± 3.5 %) led to significant increases in energy expenditure when combined with 30 minutes of treadmill exercise in comparison to calorie burning rates seen with maltodextrin (7.3 ± 4.8 %) or a non-feeding control condition (8.9 ± 6.7 %) suggesting a potential metabolic benefit.

**Acknowledgments**

This study was funded by Dymatize Nutrition Sport Performance Institute.

## P42 Effects of a pre-workout supplement on peak power and power maintenance during lower and upper body testing

### Michael T. Lane^1^, M. Travis Byrd^2^, Zachary Bell^1^, Emily Frith^1^, Lauren M.C. Lane^1^

#### ^1^Eastern Kentucky University, Richmond, KY, USA; ^2^University of Kentucky, Lexington, KY, USA

##### **Correspondence:** Michael T. Lane (michael.lane@eku.edu) – Eastern Kentucky University, Richmond, KY, USA

**Background**

The current research shows how the use of a pre-workout, performance-enhancing supplement may improve peak power production. However, there is limited research examining how, or if, this improvement of peak power is maintained during prolonged testing. The purpose of this research study was to investigate the effects of supplementation on power production and maintenance in college age males during vertical jumping, lower body high-intensity cycle ergometry sprint performance and an upper body, velocity-based strength movement.

**Methods**

Twenty-three males (22.9 ± 3.6 yrs, 175.6 ± 6.5 cm, 86.9 ± 15.1 kg, 19.1 ± 8.4 BF%) were familiarized with testing protocol and maximal bench press performances were attained. (109.1 ± 34.0 kg) Utilizing a double blind crossover design, subjects completed three trials of: five countermovement vertical jumps, before and after a high-intensity cycle sprint protocol, which consisted of ten maximal, 5 second cycle ergometer sprints, utilizing 7.5 % of the subject’s body weight as resistance, with 55 sec of recovery between each sprint. Twenty minutes prior to each trial, the subjects ingested, in a randomized order, Assault Black [Supp], Placebo+300 mg Caffeine [PL+Caff] or a Placebo [PL]). Peak power (PP) output (highest one second power output), mean power (MP) output (average power output for the 5 sec sprint) and minimum power (MNP) output (lowest one second power output) were recorded for each sprint. The average PP, MP and MNP for the 10 sprints were calculated. Then each subject performed a velocity bench press test, utilizing 80 % of each subject’s predetermined 1RM for 10 sets of 3 repetitions for maximal speed, with one-minute rest between each set. Maximal velocity and power output from each set was recorded. Blood lactate [bLa^-^] was measured immediately prior to the start of the testing session, within 2 minutes of the completion of the last cycle ergometer sprint and following the completion of the bench press test.

**Results**

Supp treatment showed significant performance improvements compared to the PL in regards to PP and maximum velocity. Wingate testing showed improved performance in PP, and total power with the Supp group compared with PL+Caff and PL groups. (p < 0.10, <.05 respectively). In the bench press, peak velocity was higher with both the Supp and PL+Caff treatments compared with placebo group (Supp: 0.689 ± 0.15w, PL+Caff: 0.69 ± 0.13w, PL: 0.644 ± 0.11w). Vertical Jump performance and lactate levels were not significantly different for any of the treatments (RMANOVA showed no significant differences from any treatments).

**Conclusions**

Supplementation with Assault Black or placebo with caffeine showed positive benefits in performance, specifically in peak power output and velocity.

**Acknowledgements**

Supported by a grant from MusclePharm Corp., and administered by the ISSN.

## P43 Effects of a pre-workout supplement on peak power production during lower and upper body testing in college-age females

### Michael T. Lane^1^, M. Travis Byrd^2^, Zachary Bell^1^, Emily Frith^1^, Lauren M.C. Lane^1^

#### ^1^Eastern Kentucky University, Richmond, KY, USA; ^2^University of Kentucky, Lexington, KY, USA

##### **Correspondence:** Michael T. Lane (michael.lane@eku.edu) – Eastern Kentucky University, Richmond, KY, USA

**Background**

The current research shows how the use of a pre-workout, performance-enhancing supplement may improve peak power production in males; however, there is limited research on the effects of pre-workout supplements on the power output and maintenance of female subjects. The purpose of this research study was to investigate the effects of supplementation on power production and maintenance in college age females during lower body a high intensity cycle ergometry sprint performance and an upper body, velocity-based strength movement.

**Methods**

Twenty-nine females (21.5 ± 1.8 yrs, 165 ± 6.1 cm, 63.1 ± 7.8 kg, 24.0 ± 7.3 BF%) were familiarized with testing protocol and maximal bench press performances were obtained (42.5 ± 14.3 kg) Using a double-blind crossover design, subjects were given either the Supplement (Supp), Placebo+Caffeine (PL+Caff) or Placebo(PL) drink twenty minutes prior to each trial in a randomized order. Subjects then completed three trials of: five countermovement vertical jumps, before and after a high intensity, Wingate cycle sprint protocol, which consisted of ten maximal, 5 sec cycle ergometer sprints, utilizing 7.5 % of the subject’s body weight as resistance with 55 sec of recovery between each sprint. Peak power (PP) output (highest one second power output), mean power (MP) output (average power output for the 5 sec sprint) and minimum power (MNP) output (lowest one second power output) were recorded for each sprint. The average PP, MP and MNP for the 10 sprints were calculated. Then each subject performed a velocity bench press test, utilizing 80 % of each subject’s predetermined 1RM for 10 sets of 3 repetitions for maximal speed, with one-minute rest between each set. Maximal velocity and power output from each set was recorded. Blood lactate [bLa^-^] was measured immediately prior to the start of the testing session, within 2 minutes of the completion of the last cycle ergometer sprint and following the completion of the bench press test.

**Results**

Supplementation with Ignite showed a statistically significant difference in many of the measures in the study. Vertical jump height was improved in both the Supp and PL+Caff treatments compared with the PL treatment (p < .05), while the Ignite treatment was associated with higher peak power during Wingate trials and peak velocity during the bench press (p < .05). Comparison of bench press peak velocity results to PL+Caff and PL treatment are as follows: Supp: 0.55 ± 0.11 m/s, PL+Caff: 0.53 ± 0.09 m/s, PL: 0.53 ± 0.15 m/s. No significant differences were seen regarding lactate response or total power produced in the bench press between the treatment groups.

**Conclusions**

Supplementation with Ignite was associated with positive improvements in vertical jump height, peak power output, and bench press velocity.

**Acknowledgements**

Supported by a grant from MusclePharm Corp., and administered by the ISSN.

## P44 A comparison of whey versus casein protein supplementation on resting metabolic rate and body composition: a pilot study

### Corey A. Peacock^1^, Tobin A. Silver^1^, Megan Colas^1^, Mauricio Mena^1^, Winter Rodriguez^1^, Gabriel J. Sanders^2^, Jose Antonio^1*^

#### ^1^Nova Southeastern University, Department of Health and Human Performance, Fort Lauderdale, FL, USA; ^2^Northern Kentucky University, Department of Kinesiology, Highland Heights, KY, USA

##### **Correspondence:** Jose Antonio PhD (ja839@nova.edu) – Nova Southeastern University, Department of Health and Human Performance, Fort Lauderdale, FL, USA

**Background**

Research from our laboratory suggests that consuming a very high protein diet may confer benefits vis a vis body composition. Although great deal of literature exists showing the benefits of both whey and casein supplementation, there is little data which compares casein versus whey with regards to body composition and resting metabolic rate. Thus, the purpose of this investigation was to compare the effects of whey versus casein protein supplementation on indices of body composition and resting metabolic rate in conjunction with a 4-week resistance-training program.

**Methods**

Twelve physically trained, healthy individuals (mean±SD; 22±4.1 yrs) completed this preliminary trial. The randomized, 2 condition [Combat**®** 100 % Isolate (Whey) versus Combat® 100 % (Casein)] intervention consisted of subjects completing 20 resistance-training sessions over a 4-week period. The resistance training protocol followed a traditional body builder split, focusing on muscle group training for volume and hypertrophy. Subjects supplemented only Whey or Casein throughout the 4-week intervention, and consumed 60 grams per day (2 × 30 g serving). One serving was mandated following all resistance training sessions. Laboratory testing sessions took place prior to the 4-week intervention (pre-) and immediately following the 4-week intervention (post-). During both laboratory testing sessions, subjects were first measured for Weight and % body fat (BF%) utilizing the Bod Pod®. Subjects were then measured for resting metabolic rate (RMR) utilizing the Parvo Metabolic Cart (Parvomedics Inc., Sandy, UT).

**Results**

A 2 time-point by 2 condition, repeated-measures analysis of variance (ANOVA) demonstrated non-significant (*p* ≥ 0.05) effects on body weight, body fat percentage and RMR (Table 9).

**Conclusion**

There are no differences in casein versus whey protein supplement with regards to body composition and RMR in trained subjects over a 4-week treatment period. Future research will examine different treatment periods as well as dosage.

**Acknowledgements**

This study was funded with a grant from MusclePharm®.

**Table 9 (abstract P44):** Body Composition and Resting Metabolic Rate

	Whey (n=6)		Casein (n=6)	
	Pre	Post	Pre	Post
Weight kg	77.7±9.4	77.6±8.1	74.4±11.3	75.1±11.3
% Body Fat	12.6±6.8	12.8±7.2	14.7±6.9	15.5±7.1
RMR	1969±456	1919±360	1648±285	1570±644

Data are mean±SD. There were no significant differences within or between groups

## P45 A novel mixed-tocotrienol intervention enhances recovery after eccentric exercise: preliminary findings

### Andrea Vansickle, Brittany DiFiore, Stephanie Stepp, Grant Slack, Bridget Smith, Kayla Ruffner, Ronald Mendel, Lonnie Lowery

#### University of Mount Union, Alliance, OH, USA

##### **Correspondence:** Lonnie Lowery (LoweryLM@MountUnion.edu) – University of Mount Union, Alliance, OH, USA

**Background**

Eccentric exercise is known to induce changes in muscle morphology (swelling, contracture, joint angle), psychometric variables (delayed-onset muscle soreness), and performance (power output). Part of the process is metabolic damage and muscle micro-trauma that involves leukocyte activation and increased reactive oxygen and nitrogen species. Tocotrienols act as antioxidants and have other qualities that may apply to eccentric recovery. We hypothesized that a novel form of vitamin E would enhance the recovery from eccentric exercise in comparison to an identical placebo.

**Methods**

In order to investigate the effects of two weeks’ supplementation with a daily 20 mg “melt-and-swallow” tocotrienol product (Gordagen nE1-ElitE), 14 Division III football players and similarly-trained weight trainers underwent an eccentric exercise session of 25 total repetitions using 80 % one-repetition maximum (1RM) in the biceps curl, bench press and squat. Subjects were instructed to maintain their usual diets throughout the study. The mixed tocotrienol group (TOC, n=8) and placebo (PBO, n=6) were compared pre- and 24 h-post-eccentric exercise in hanging arm angle (elbow goniometer), Likert-type (1-5 scale) soreness, and 30s Wingate cycling power. RPE (6-20 Borg Scale) was also assessed pre- and post-eccentric exercise during the Wingate test.

**Results**

Compared to the pre-eccentric exercise baseline, TOC responded differently to the “damaging” exercise at 24 h compared to PBO based on factorial ANOVA and Duncan post-hoc analyses where appropriate. There was an interaction for hanging arm angle (TOC 173.5±3.5^o^ [pre] vs. 169.6± 2.3^o^ [post]; and PBO 173.2± 4.9^o^ [pre] vs. 165.8± 6.1^o^ [post]; p=0.02) and Likert-type soreness (TOC 2.1±1.1 [pre] vs. 1.9±0.8 [post]; and PBO 1.5±0.6 [pre] vs. 2.8± 0.9 [post]; p=0.05). There was also a trend toward enhanced power production during the Wingate cycling test (TOC 813.8±161.4 W [pre] vs. 838.6±142.2 W [post]; and PBO 850.2±145.5 W [pre] vs. 788.8±119.3 W [post]=0.056). No differences in rating of perceived exertion (RPE) were noted within or between groups.

**Conclusions**

Within the parameters of this design, we conclude that our hypothesis was supported: The under-the-tongue mixed tocotrienol intervention enhanced various aspects of recovery 24 h after eccentric exercise.

**Acknowledgements**

This early-phase research was supported by a grant from Gordagen Pharmaceuticals, Victoria, Australia.

## P46 The effects of post-exercise ingestion of a high molecular weight glucose on cycle performance in female cyclists

### Katie R. Hirsch^1^, Meredith G. Mock^1^, Malia M.N. Blue^1^, Eric T. Trexler^1^, Erica J. Roelofs^2^, Abbie E. Smith-Ryan^1^

#### ^1^Applied Physiology Laboratory, Department of Exercise and Sport Science, University of North Carolina, Chapel Hill, NC, USA; ^2^Nutrition, Health, and Human Performance Department, Meredith College, Raleigh, NC, USA

##### **Correspondence:** Katie Hirsh (ktrose23@live.unc.edu) – Applied Physiology Laboratory, Department of Exercise and Sport Science, University of North Carolina, Chapel Hill, NC, USA

**Background**

Low molecular weight carbohydrate solutions (LMW) are commonly used in commercially available sport drinks for rapid glycogen resynthesis during long-duration aerobic exercise. In contrast, consumption of a high molecular weight glucose polymer solution (HMW) has previously been shown to increase work output during a 15-min time-trial compared to LMW in male cyclists, but potential benefits have not been tested in females. Therefore, the purpose of this study was to compare the effects of a HMW solution with an isoenergetic LMW solution on cycling performance in women.

**Methods**

In a randomized, double-blind, placebo controlled, cross-over design, ten competitive female cyclists (Mean ± SD; age = 25.7 ± 5.0 yrs; height = 167.7 ± 6.0 cm; weight = 62.6 ± 4.2 kg; VO_2_max = 49.7 ± 4.3 ml∙kg^-1^∙min^-1^) consumed three different carbohydrate solutions: LMW (maltodextrin/dextrose/fructose); HMW (VitargoR); or control (CON; non-caloric flavoring) on three randomly ordered visits separated by 7-10 days. Visits consisted of a ride-to-exhaustion (RTE) at a workload equal to 75 % of maximal oxygen uptake (VO_2_max), determined from a maximal graded exercise test at a prior visit. Immediately upon completion, subjects consumed a 700 mL solution containing 1.2 g∙kg^-1^ of either LMW or HMW, or 0.066 g∙kg^-1^ CON to match for taste. After a two-hour rest period, subjects performed a 15-minute time-trial at a self-adjusted speed and resistance, completing as much work as possible to determine total distance and average watts. To assess hydration status, total body water (TBW), intracellular water (ICW), and extracellular water (ECW) were measured using bioelectrical impedance prior to the RTE and again before the 15-minute time-trial. Palatability and gastrointestinal distress were evaluated using hedonic and visual analog scales (VAS), respectively.

**Results**

There were no significant differences in average time (98.0 ± 15 min) or distance (22.3 ± 4.6 km) for the RTE between treatments (p>0.05). There was no significant treatment effect (p=0.438) on 15-min time-trial total distance between LMW = 6.8 ± 1.8 km, HMW = 7.1 ± 1.1 km, and CON =7.6 ± 1.1 km. There was no treatment effect on average watts (p=0.594) between LMW = 161.6 ± 35.9 W, HMW = 164.5 ± 32.3 W, and CON = 161.6 ± 30.9 W. TBW was not significantly different pre to post for LMW (p=0.117), but was significantly reduced for HMW (p=0.007) and CON (p=0.044). ECW was significantly higher at pre than post for all trials (p<0.001); there were no changes in ICW. There was no significant treatment effect on hedonic scores (p=0.407; 5.4 ± 1.1) or VAS scores pre- to post-exercise (p=0.724).

**Conclusion**

Despite previous positive results of HMW in cycling performance in males, current results demonstrate no ergogenic effect of HMW or LMW on cycling performance in females. Carbohydrate alone may not be the most efficient way to rapidly resynthesize glucose in female cyclists. Due to the large proportion of branched chain amino acids oxidized during aerobic exercise in females, especially under fasted conditions, a combination of carbohydrates and protein may be more appropriate for glycogen resynthesis and subsequent improvements in performance.

**Acknowledgments**

This study was funded in part by Vitargo, Inc.

## P47 Inclusive vs. exclusive dieting and the effects on body composition in resistance trained individuals

### Laurin Conlin^1^, Danielle Aguilar^1^, Bill I. Campbell^1^, Layne Norton^1^, Katie Coles^1^, Eric T. Trexler^2^, Nic Martinez^1^

#### ^1^University of South Florida, Performance & Physique Enhancement Laboratory, Tampa, FL, USA; ^2^Human Movement Science Curriculum, University of North Carolina, Chapel Hill, NC, USA

##### **Correspondence:** Bill I. Campbell (bcampbell@usf.edu) – University of South Florida, Performance & Physique Enhancement Laboratory, Tampa, FL, USA

**Background**

There is limited research on how rigid vs. flexible dieting strategies affect weight loss and even less research investigating these effects on weight regain. The purpose of this study was to compare an inclusive vs. exclusive diet on weight loss and subsequent weight regain in resistance-trained (RT) males and females in a randomized, parallel group design.

**Methods**

Twenty-seven resistance-trained male (n=11) and female (n=16) subjects (25 ± 5.7 years; 169.6 ± 8.3 cm; 75.7 ± 11.4 kg) participated in this investigation. A 20-week trial period consisting of a 10-week diet phase and a 10-week post-diet phase was implemented for this study. Participants were randomized to an inclusive diet (ID; a flexible diet comprised of non-specific foods) or an exclusive diet (ED; a rigid diet comprised of specific foods) group. Participants adhered to a 25 % kcal reduction from their current calorie consumption during the first 10-weeks of the intervention (diet phase) and then were instructed to eat however they chose for the final 10-weeks of the study (post-diet phase). Also, participants were asked to not make changes to their current training program. Body composition was assessed via amplitude mode ultrasonography using a seven-site skinfold equation to evaluate percent body fat (%fat), fat mass (FM), and fat-free mass (FFM). Measurements were taken at 5 time points: (baseline, 5, 10,[end of diet phase], 16, and 20 weeks). Data were analyzed via a 2-factor [2x5] between-subjects repeated measures analysis of variance (ANOVA). The criterion for significance was set at p ≤ 0.05.

**Results**

No baseline differences existed between the two dietary groups on any dependent variable. During the 10-week weight loss phase, both groups significantly reduced bodyweight (ID: baseline = 77.5 ± 10.5 kg, post-diet = 75.6 ± 11.5 kg; ED: baseline = 73.9 ± 12.4 kg, post-diet = 71.3 ± 11.6 kg; p = 0.002); fat mass (ID: baseline = 16.7 ± 8.4 kg, post-diet = 15.0 ± 8.8 kg; ED: baseline = 16.7 ± 6.8 kg, post-diet = 14.1 ± 6.4 kg; p< 0.001) and body fat% (ID: baseline = 21.6 ± 9.3 %, post-diet = 19.5 ± 8.9 %; ED: baseline = 22.5 ± 7.4 %, post-diet = 19.9 ± 7.5 %; p< 0.001). FFM did not change during the diet phase. During the post-diet phase, no significant differences were observed between the dietary groups for body weight, fat mass, and body fat%. In contrast, a significant diet x time interaction (p < 0.001) was observed for FFM with the inclusive group gaining a significant amount of FFM (+1.53 kg) in comparison with the exclusive group (-0.59 kg).

**Conclusions**

An inclusive or an exclusive diet strategy is equally effective for weight loss during a caloric restriction diet in free-living, RT individuals. It appears that a flexible diet is beneficial in creating a greater anabolic environment post-diet. During the post-diet period, there were no differences in time spent in resistance and aerobic exercise modes nor were there any differences in protein intakes between the two diet groups.

More research should evaluate additional physiological effects of inclusive diets and weight regain in a variety of individuals (including lean individuals).

**Acknowledgements**

This study was supported by the Biolayne Foundation Research Grant

## P48 A whey protein hydrolysate may positively augment resting metabolism compared to intact whey protein

### Jordan M. Joy^1,2^, Roxanne M. Vogel^1^, Thomas H. Hoover^1^, K. Shane Broughton^1^

#### ^1^Department of Nutrition and Food Sciences, Texas Woman’s University, Denton, TX, USA; ^2^InnovaSolutions LLC, Denton, TX, USA

##### **Correspondence:** Jordon Joy (jmjoyx@gmail.com) – InnovaSolutions LLC, Denton, TX, USA

**Background**

Dietary proteins, often as supplemental whey proteins, are known to increase energy expenditure relative to isocaloric, non-protein or low-protein controls. Through enzymatic hydrolysis, whey proteins become partially “pre-digested” whey protein hydrolysates (WPH), which are more rapidly absorbed following consumption, yet knowledge is limited concerning practical applications of WPH. Limited data suggest WPH may be advantageous to whey protein concentrates (WPC) for reducing body fat, possibly by modulating post-prandial metabolism. Therefore, the purpose of this investigation was to examine the acute metabolic responses to isocaloric WPH, WPC, and carbohydrate supplements.

**Methods**

In a single-blind, within-subjects design, 7 recreationally-active males (175.3±4.8 cm; 77.8±13.8 kg; 23.3±2.4 years; 62.3±11.3 kg lean) ingested 0.3 g/kg lean soft tissue WPH (Lacprodan Hydro.365, Arla Foods Ingredients, Denmark), WPC (WPC80, Arla Foods Ingredients, Denmark), or carbohydrate (maltodextrin) and had their resting energy expenditure (REE) and respiratory quotient (RQ) measured using indirect calorimetry over a 3-hour post-prandial period and compared to a pre-treatment, fasting baseline. Average dose of treatment was 18.7 g of protein or carbohydrate. Participants were given 10 minute breaks after each hour of testing. Data were collected at pre, 15, 30, 45, 60, 90, 105, 120, 150, 165, and 180 minutes using the average of the previous 10 data points. Thermogenic effect of food (TEF) was estimated from the AUC of the REE delta values from baseline. Data were analyzed using 3x11 (treatment x time) repeated-measures ANOVA. Calculated delta and TEF values were analyzed using one-way ANOVA. If significant interactions were observed, Bonferroni post-hoc tests were used to locate differences. Data are reported as mean±standard deviation.

**Results**

No differences existed at baseline between treatments for REE (WPH: 0.015±0.003; WPC: 0.015±0.002; carbohydrate: 0.015±0.002 Cal/minute) or RQ (WPH: 0.81±0.04; WPC: 0.80±0.04; carbohydrate: 0.81±0.04). Significant interactions (p<0.05) were observed for REE and RQ. Wherein, WPH and WPC elevated REE at 45 (WPH: 0.164±0.099; WPC: 0.182±0.082 Cal/minute) and 60 (WPH: 0.211±0.162; WPC: 0.218±0.111 Cal/minute) minutes post-ingestion versus carbohydrate (45 m: 0.092±0.117; 60 m: 0.044±0.064 Cal/minute). Only WPH lowered RQ compared to carbohydrate at 90 minutes post-ingestion (WPH: 0.791±0.03; carbohydrate: 0.844±0.03). Delta from baseline indicated significant (p<0.05) increases in REE for both WPH (+0.026±0.069 Cal/minute) and WPC (+0.096±0.074 Cal/minute) versus carbohydrate (-0.073±0.033 Cal/minute) only at 90 minutes post-ingestion. TEF was significantly (p<0.05) greater for WPH (+12.2±8.0 Cal) and WPC (+11.9±4.4 Cal) than carbohydrate (+3.5±3.7 Cal) beginning at 90 minutes and remained greater through 180 minutes (WPH: +19.3±12.1 Cal; WPC: +19.9±8.1 Cal; carbohydrate: +3.8±9.2 Cal). RQ changes from baseline to 90 minutes and to 105 minutes post-ingestion were significant (p<0.05) only for WPH (90 m: -0.022±0.02; 105 m: -0.017±0.02) versus carbohydrate (90 m: +0.036±0.05; 105 m: +0.032±0.05).

**Conclusions**

Both WPH and WPC induce equal increases in REE over 3 hours versus carbohydrate of isocaloric dosage. WPH, but not WPC, significantly influenced substrate utilization with a directional shift favoring lipid metabolism versus the carbohydrate treatment, which may help explain previous findings of fat loss with WPH, but not WPC.

**Acknowledgements**

Supported by Arla Foods Ingredients Group P/S (Aarhus, Denmark) and Dymatize Nutrition Sport Performance Institute (Dallas, TX).

## P49 Seven days of high and low dose creatine nitrate supplementation I: hepatorenal, glucose and muscle enzyme function

### R Dalton^1^, R Sowinski^1^, T Grubic^1^, PB Collins^1^, A Colletta^1^, A Reyes^1^, B Sanchez^1^, M Kozehchain^1^, YP Jung^1^, C Rasmussen^1^, P Murano^2^, CP Earnest^1,3^, M Greenwood^1^, RB Kreider^1^

#### ^1^Exercise & Sport Nutrition Lab, Texas A&M University, College Station, TX, USA; ^2^Institute for Obesity and Program Evaluation, Texas A&M University, College Station, TX, USA; ^3^Nutrabolt, Bryan, TX, USA

##### **Correspondence:** R Dalton (ryanldalton@tamu.edu) – Exercise & Sport Nutrition Lab, Texas A&M University, College Station, TX, USA

**Background**

Creatine and nitrates are popular dietary supplements. While both have been examined in singularity, little is known regarding their co-ingestion relative to performance, side effects and safety. The purpose of this study was to examine the safety and efficacy of a creatine nitrate dietary supplement.

**Methods**

In a double-blind, crossover, randomized and placebo-controlled manner; 28 apparently healthy and recreationally active men and women (18 men, 10 women, 21.6±3.7 yr, 20.4±10.6 % fat, 24.7±2.9 kg/m^2^) ingested daily supplements for 7-d consisting of a dextrose flavored placebo (PLA); a low dose of creatine nitrate (Low, 3 g) and a high dose of creatine nitrate (6 g). Participants repeated the experiment with the alternate supplements randomly with a 7 day washout period between each. Participants had each blood donation after 8+ hours fasting on days 1, 2, 6, and 7 for each supplement. Data were analyzed by repeated measure 4 x 3 MANOVA with Time and Group as factors using Greenhouse-Geisser as appropriate and are presented as mean±SD or mean change from baseline ± 95 % CI.

**Results**

Significant time effects (p<0.001) were observed for blood urea nitrogen (BUN), alkaline phosphatase (ALP), LDL cholesterol (LDL), triglycerides (TRI), and glucose (GLU) (p<.01). Significant group x time interactions were found for CRE (p<.05). No significant differences for time, group, or interactions were found for aspartate amino transferase, and alanine amino transferase, for total cholesterol, HDL cholesterol, creatine kinase, and lactate dehydrogenase (p>0.05). Respective blood values at Day1, Day2, Day6, and Day7 are: BUN (5.07±1.29, 5.00±1.47, 5.29±1.42, 5.26±1.49 mmol/L) with Day2 lower than days 3 & 4, ALP (80.43±22.19, 77.85±20.14, 81.10±21.35, 79.60±20.77 U/L) with Day2 lower than days 1 & 3, LDL (2.61±1.32, 2.53±1.17, 2.29±0.96, 2.35±0.96 mmol/L) with Day3 lower than days 1, 2, & 4, TRI (0.88±0.39, 0.81±0.31, 0.91±0.42, 0.83±0.33 mmol/L) with Day2 lower than days 1 & 3, GLU (5.05±0.48, 4.89±0.40, 4.96±0.54, 5.03±0.44 mmol/L) with Day2 lower than days 1&4, and mean changes for CRE (Day2 PLA: 1.60, 95 % CI -2.00, 5.18, Day2 Low: -1.67, 95 % CI -5.26, 1.92, Day2 High: 4.25, 95 % CI 0.66, 7.84, Day6 PLA: -0.53, 95 % CI -5.38, 4.33, Day6 Low: -3.37, 95 % CI 8.22, 1.49, Day6 High: 7.58, 95 % CI 2.72, 12.43, Day7 PLA: -1.13, 95 % CI -5.32, 3.05, Day7 Low: -5.83, 95 % CI -10.01, -1.65, Day7 High: 4.45, 95 % CI 0.27, 8.64 μmol/L). CRE was found to be higher at Day2 for High compared Low and at Day6 and Day7 for High vs Low & PLA, CRE increased over time for group High compared to PLA and Low, which did not change.

**Conclusion**

Ingesting a creatine nitrate supplement demonstrated minor time x group interaction differences in CRE. However, these changes did not exceed normal clinical limits. This and the results observed for other clinical markers associated with hepatorenal and muscle enzyme function demonstrate that creatine nitrate appears to be safe when ingested for seven days.

**Acknowledgments**

This study was supported by Nutrabolt (Bryan, TX) through an unrestricted grant to Texas A&M University.

## P50 Seven days of high and low dose creatine nitrate supplementation II: performance

### T Grubic^1^, R Dalton^1^, R Sowinski^1^, PB Collins^1^, A Colletta^1^, A Reyes^1^, B Sanchez^1^, M Kozehchain^1^, YP Jung^1^, C Rasmussen^1^, P Murano^2^, CP Earnest^1,3^, M Greenwood^1^, RB Kreider^1^

#### ^1^Exercise & Sport Nutrition Lab, Texas A&M University, College Station, TX, USA; ^2^Institute for Obesity and Program Evaluation, Texas A&M University, College Station, TX, USA; ^3^Nutrabolt, Bryan, TX, USA

##### **Correspondence:** R Dalton (ryanldalton@tamu.edu) – Exercise & Sport Nutrition Lab, Texas A&M University, College Station, TX, USA

**Background**

Creatine and nitrates are popular dietary supplements. While both have been examined in singularity, little is known regarding their co-ingestion relative to performance, side effects and safety. The purpose of this study was to examine the safety and efficacy of a creatine nitrate dietary supplement.

**Methods**

In a double-blind, crossover, randomized and placebo-controlled manner; 28 apparently healthy and recreationally active men and women (18 men, 10 women, 21.6±3.7 yr, 20.4±10.6 % fat, 24.7±2.9 kg/m^2^) ingested daily supplements for 7-d consisting of a dextrose flavored placebo (PLA); a low dose of creatine nitrate (Low, 3 g) and a high dose of creatine nitrate (6 g). Participants repeated the experiment with the alternate supplements randomly with a 7 day washout period between each. Performance outcomes were: Bench and Leg Press 1RM, reps to fatigue during the 3^rd^ set of BP and LP 1RM at 70 % 1RM repeated 30 mins post supplement on days 1 and 6 and 4 km cycling time trial performance on days 2 and 7. Data were analyzed by repeated measure 4 × 3 × 2 MANOVA with Time, Group, and Gender as factors using Greenhouse-Geisser as appropriate and are presented as mean±SD.

**Results**

No significant group x time interactions were observed among supplementation groups. Therefore, results for the pooled cohort, separated by gender, are presented. Overall, we observed significant time, gender, and time x gender interaction for bench press and leg press 1RM and bench press lifting reps to fatigue (*all*, p<0.001), as well as for time to complete the 4-km trial (p<0.01) and average time trial power (p<0.001). A time x gender interaction for only bench press 1RM was observed. Female vs. Male bench press 1RM are: Day1 (89.2±16.9 vs. 203.6±43.8), Day1 Post Supplement (84.2±18.4 vs. 185.9±46.7), Day6 (92.2±19.5 vs. 206.5±45.2), Day 6 Post Supplement (87.3±16.7 vs. 193.0±46.4 lbs). Female vs. Male leg press 1RM: Day1 (630.2±113.0 vs.1047.7±210.9), Day1 Post Supplement (606.3±111.1 vs. 1002.9±215.4), Day6 (652.5±121.1 vs. 1062.9±215.4), Day 6 Post Supplement (611.3±106.4 vs. 1020.4±213.7 lbs). All comparisons are significantly higher for males vs. females. Women decreased less post supplement compared to men for bench press 1RM. Female vs. Male reps to fatigue for the BP are: Day1 (14.9±6.0, vs. 13.7±4.8), Day1 Post Supplement (15.6±4.4 vs. 13.2±4.7), Day6 (15.9±5.2 vs. 14.1±4.9), and Day6 Post Supplement (17.2±4.9 vs. 14.5±5.2). All comparisons are significantly higher for females vs. males. No differences were found for leg press reps to fatigue. Time trial performance was significantly slower for women vs. men, respectively, at all testing time points: Day2 (390.8±79.3, vs. 209.9±35.2), Day7 (390.0±97.9 vs. 203.9±3.6 sec), with men also demonstrating significantly higher average power: Day2 (158.6±26.8, vs. 292.9±54.5), Day7 (161.6±32.3vs. 304.1±59.3 sec).

**Conclusion**

Ingesting 3 and 6 g/d of creatine nitrate for 7-days had no significant effects on performance measures in comparison to ingesting a placebo. Males were found to be stronger than females, but with significantly lower bench press endurance. Males also performed better on the bicycle tests regarding time to complete the time trial and the average power output associated with the cycling task.

**Acknowledgments**

This study was supported by Nutrabolt (Bryan, TX) through an unrestricted grant to Texas A&M University.

## P51 Seven days of high and low dose creatine nitrate supplementation III: hemodynamics

### R Sowinski^1^, R Dalton^1^, T Grubic^1^, PB Collins^1^, A Colletta^1^, A Reyes^1^, B Sanchez^1^, M Kozehchain^1^, YP Jung^1^, C Rasmussen^1^, P Murano^2^, CP Earnest^1,3^, M Greenwood^1^, RB Kreider^1^

#### ^1^Exercise & Sport Nutrition Lab, Texas A&M University, College Station, TX, USA; ^2^Institute for Obesity and Program Evaluation, Texas A&M University, College Station, TX, USA; ^3^Nutrabolt, Bryan, TX, USA

##### **Correspondence:** R Dalton (ryanldalton@tamu.edu) – Exercise & Sport Nutrition Lab, Texas A&M University, College Station, TX, USA

**Background**

Creatine and nitrates are popular dietary supplements. While both have been examined in singularity, little is known regarding their co-ingestion relative to performance, side effects and safety. The purpose of this study was to examine the safety and efficacy of a creatine nitrate dietary supplement.

**Methods**

In a double-blind, crossover, randomized and placebo-controlled manner; 28 apparently healthy and recreationally active men and women (18 men, 10 women, 21.6±3.7 yr, 20.4±10.6 % fat, 24.7±2.9 kg/m^2^) ingested daily supplements for 7-d consisting of a dextrose flavored placebo (PLA); a low dose of creatine nitrate (Low, 3 g) and a high dose of creatine nitrate (6 g). Participants repeated the experiment with the alternate supplements randomly with a 7 day washout period between each. Participants had systolic and diastolic blood pressure and heart rate measured after lying supine for 15 minutes and again after 2 minutes lying vertical on a tilt table. This was conducted both pre and 30 minutes post supplement, post resistance exercise on days 1 and 6. Data were analyzed by repeated measure 8 x 3 MANOVA with Time and Group as factors using Greenhouse-Geisser as appropriate and are presented as mean change from Day1 Supine ± 95%CI.

**Results**

Statistical analysis revealed significant changes over time for DBP and heart rate (HR) (p<.001), but not SBP (p>0.05). No significant group differences or group x time interactions were observed among groups. Therefore, the following reflects pooled cohort time point comparisons. Mean change± 95%CI from Day1 Supine for DBP (mmHg) are: Day 1 vertical (2.8, 95%CI 1.3, 4.3), Day1 Post Supplement, Post Exercise Supine (0.7, 95%CI -0.8, 2.3), Day1 Post Supplement, Post Exercise Vertical (4.0, 95%CI 2.2, 5.8), Day 6 supine (0.8, 95%CI -1.3, 2.9), Day6 vertical (3.8, 95%CI 1.5, 6.2), Day6 Post Supplement, Post Exercise Supine (1.0, 95%CI -1.1, 3.0), and Day6 Post Supplement, Post Exercise Vertical (3.5, 95%CI 1.3, 5.6). Significant differences were found between all supine and vertical DBP measures except between Day1 Vertical and Day6 Supine & Day6 Post Supplement, Post Exercise Supine. Mean change ± 95%CI for HR (bpm) are: Day 1 vertical (11.8, 95%CI 10.0, 13.5), Day1 Post Supplement, Post Exercise Supine (14.5, 95%CI 12.3, 16.6), Day1 Post Supplement, Post Exercise Vertical (24.4, 95%CI 21.8, 27.0), Day 6 supine (-1.4, 95%CI -3.4, 0.5), Day6 vertical (12.4, 95%CI 10.0, 14.8), Day6 Post Supplement, Post Exercise Supine (15.2, 95%CI 12.5, 17.9), and Day6 Post Supplement, Post Exercise Vertical (24.8, 95%CI 21.5, 28.0). Significant differences were found between all time points, Day1 Supine & Day6 Supine, Day1 Vertical & Day6 Vertical, Day1 Post Supplement, Post Exercise Supine & Day6 Vertical, Day1 Post Supplement, Post Exercise Supine & Day6 Post Supplement, Post Exercise Supine, and Day1 Post Supplement, Post Exercise Vertical & Day6 Post Supplement, Post Exercise Vertical.

**Conclusion**

Changes in body position and exercise increased DBP and HR. However, supplementation of 3 or 6 g/d of creatine nitrate did not significantly affect hemodynamic responses in comparison to a placebo.

**Acknowledgments**

This study was supported by Nutrabolt (Bryan, TX) through an unrestricted grant to Texas A&M University.

## P52 The efficacy of a β-hydroxy-β-methylbutyrate supplementation on physical capacity, body composition and biochemical markers in highly-trained combat sports athletes

### Krzysztof Durkalec-Michalski^1,2^, Jan Jeszka^1^, Tomasz Podgórski^3^

#### ^1^Department of Hygiene and Human Nutrition, Poznan University of Life Sciences, Wojska Polskiego 31, 60-624 Poznan, Poland; ^2^Polish Wrestling Federation, Żelazna 67/73, 00-871 Warsaw, Poland; ^3^Department of Biochemistry, University School of Physical Education in Poznan, Królowej Jadwigi 27/39, 61-871 Poznań, Poland

##### **Correspondence:** Krzysztof Durkalec-Michalski (durkmich@up.poznan.pl) – Polish Wrestling Federation, Żelazna 67/73, 00-871 Warsaw, Poland

**Background**

β-hydroxy-β-methylbutyric acid (HMB) is a popular supplement in sports from almost 20 years. Some published studies claim that HMB uptake may promote anticatabolic action, changes in body composition, strength, and reduced levels of muscle damage, as well as promote the aerobic capacity. Drawn attention the fact that the effect of HMB uptake on physical capacity has rarely been verified in combat sports. In view of the inconclusive character of the study results conducted to date, and the relatively low number of studies investigating the effectiveness of HMB over a longer period on athletes, the aim of this study was to verify the effect of HMB supplementation on physical capacity, body composition and the levels of biochemical parameters in highly-trained combat sports athletes.

**Methods**

42 highly-trained in combat sports males was subjected to 12 weeks supplementation with HMB (3x1g_HMB_·day^-1^) and a placebo (PLA) in randomised, placebo controlled, double-blind crossover trials. Over the course of the experiment, aerobic [exercise test with increasing intensity on a cycloergometer (Kettler, Germany), using a ergospirometer (K4b^2^, Cosmed, Italy)] and anaerobic [classical Wingate test (Monark 894E, Sweden)] capacity were determined, while analyses were conducted on body composition [bioelectric impedance (BIA 101S AKERN-RJL, Italy)] as well as levels of creatine kinase, lactate dehydrogenase, testosterone, cortisol and lactate (commercial diagnostic tests). A normal distribution of variables was tested using the paired 2-tailed t-tests; the Mann–Whitney *U*-test or the Wilcoxon-signed rank test were applied for non-normally distributed variables.

**Results**

Following HMB supplementation fat-free mass increased (+0.7 kg_HMB_ vs. -0.5 kg_PLA_, *P*=0.02), with a simultaneous reduction of fat mass (-0.8 kg_HMB_ vs. +0.7 kg_PLA_, *P*<0.001). In turn, after HMB supplementation, in comparison to placebo: time to reach ventilatory threshold (+58 s_HMB_ vs. -25 s_PLA_, *P*<0.0001), threshold load (+15 W_HMB_ vs. -6 W_PLA_, *P*=0.005), and the threshold HR (+7 bpm_HMB_ vs. -2 bpm_PLA_, *P*<0.0001), as well as anaerobic peak power (+1.15 W·kg^-1^_HMB_ vs. -0.23 W·kg^-1^_PLA_, *P*<0.001), average power (+0.31 W·kg^-1^_HMB_ vs. -0.07 W·kg^-1^_PLA_, *P*=0.016), and post-exercise lactate concentrations (+1.6 mmol·L^-1^_HMB_ vs. -0.1 mmol·L^-1^_PLA_, *P*=0.02) increased significantly. However, in relation to the placebo, no differences were observed in blood marker levels.

**Conclusion**

The results indicate that supplying HMB promotes advantageous changes in body composition and stimulates an increase in aerobic and anaerobic capacity in combat sports athletes, while seems not to significantly affect the levels of the analyzed blood markers.

**Acknowledgments**

The authors wish to thank the coaches and athletes for their help and participation in the research project. We gratefully acknowledge financial support for this work provided by the Polish National Science Centre, grant number N N312 262340.

## P53 Does protein and source impact substrate oxidation and energy expenditure during and after moderate intensity treadmill exercise?

### C Kerksick, B Gieske, R Stecker, C Smith, K Witherbee

#### School of Sport, Recreation and Exercise Sciences, Lindenwood University, St. Charles, MO USA

##### **Correspondence:** C Kerksick (ckerksick@lindenwood.edu) – School of Sport, Recreation and Exercise Sciences, Lindenwood University, St. Charles, MO USA

**Background**

Performing cardio while fasted is a popular strategy to stimulate greater fat burning, however, recent evidence indicates being fed may impact rates of fuel oxidation. Studies suggest that protein ingestion can trigger greater increases in energy expenditure and fat oxidation, but no studies to date have examined any impact of the source of protein on energy expenditure and fat oxidation during as well as post-exercise.

**Methods**

Eleven healthy, college-aged males (23.5 ± 2.1 years, 86.0 ± 15.6 kg, 184 ± 10.3 cm, 19.7 ± 4.4 %fat) completed four identical testing sessions in a randomized, counter-balanced, crossover fashion. All participants abstained from > 200 mg caffeine ingestion and other supplementation except for protein and multi-vitamins for 30 days prior to arriving for all testing after observing an overnight fast. Upon arrival participants completed a resting metabolic rate assessment using indirect calorimetry for determination of baseline substrate oxidation and energy expenditure. In a randomized, double-blind, crossover fashion, participants then ingested similar colored, isovolumetric (12 fl oz. cold water) solutions mixed with either whey isolate or casein protein powder delivering 25 g protein or an isocaloric maltodextrin powder or a non-caloric control condition. Participants then sat quietly for 30 minutes before completing a standardized warm-up protocol. Participants were then connected to a metabolic cart where they completed 30 minutes of treadmill exercise at 55 % heart rate reserve. Approximately 15 minutes after completing the exercise bout, study participants then completed a second resting metabolic rate assessment. All supplement ingestion, warm-up completion and exercise was directly supervised by a study investigator.

**Results**

4 × 2 mixed factorial ANOVA with repeated measures on test were completed to determine any significant main and interaction effects. Resting energy expenditure data was normalized to body mass (in kg) and a significant group x time interaction (p = 0.002) was found. Oneway ANOVA with the delta scores were found to be significant (p = 0.002) and post-hoc comparisons indicated that whey (3.41 ± 1.63 kcal/kg) was greater (p<0.05) than maltodextrin (1.57 ± 0.99 kcal/kg, p = 0.010) and tended to be greater than the non-feeding control group (2.00 ± 1.91 kcal/kg, p = 0.055). Casein (3.38 ± 0.82 kcal/kg) was greater than maltodextrin (p = 0.012) and tended to be greater than the non-feeding control group (p = 0.061). No significant group x time interaction effect (p = 0.116) was found for respiratory exchange ratio data, however, whey and casein were found to significantly decrease (p<0.05) during post exercise period while no change (p>0.05) was seen for maltodextrin or the non-feeding control group.

**Conclusions**

Consuming supplemental levels of protein or maltodextrin might not significantly impact fuel oxidation substrates ratio as commonly believed. Additionally, pre-exercise feeding with whey (15.9 ± 8.3 %) and casein (15.4 ± 3.5 %) led to significant increases in energy expenditure when combined with 30 minutes of treadmill exercise in comparison to calorie burning rates seen with maltodextrin (7.3 ± 4.8 %) or a non-feeding control condition (8.9 ± 6.7 %) suggesting a potential metabolic benefit.

**Acknowledgments**

This study was funded by Dymatize Nutrition Sport Performance Institute.

## P54 Effects of 30 days of Cleanse™ supplementation on measure of body composition, waist circumference, and markers of gastrointestinal distress in females

### Stacie Urbina, Emily Santos, Katelyn Villa, Alyssa Olivencia, Haley Bennett, Marissa Lara, Cliffa Foster, Colin Wilborn, Lem Taylor

#### Department of Exercise & Sports Science, Human Performance Lab, University of Mary Hardin-Baylor, Belton, TX, USA

##### **Correspondence:** Lem Taylor (LTaylor@umhb.edu) – Department of Exercise & Sports Science, Human Performance Lab, University of Mary Hardin-Baylor, Belton, TX, USA

**Background**

The purpose of this study was to examine the effects of chronic supplementation (i.e. 30 days) of Cleanse™ on measures of body composition, waist circumference, hydration, and blood clinical safety profiles in healthy females.

**Methods**

Twenty-two physically fit females (20.7 ± 12 years, 164.7 ± 30.9 cm, 63.65 ± 37.4 kg, 23.27 ± 9.2 kg/m^2^) participated in a 30-day double-blind placebo controlled study. Participants were randomly assigned to two groups (CL= 1350 mg Cleanse, PL= 1350 mg Maltodextrin) based on body fat mass. Participants reported to the Human Performance Lab for two testing sessions (Day 0 and Day 30) to complete various lab tests. Dependent variables included body composition (FM, LM, BF%), waist circumferences, and scores on the GSRS questionnaire. Data was analyzed using repeated measure ANOVAs and the a-priori p-value was set at 0.05.

**Results**

The results indicated that supplementation produced a significant (group x time interaction) difference in waist circumference measurements with a significant difference (p < 0.05) in upper waist circumference measures for the CL group while an increase was observed in the PL group. No significant main effects (group or time) were observed for percent body fat via DEXA and InBody. Additionally, there were no observed differences in the questionnaires (GSRS) were observed for markers of gastrointestinal distress.

**Conclusion**

The findings suggest that the dietary supplement Cleanse™ does have potential benefits in terms of reducing waist circumference measurements but it should be noted that these changes are not the result of an improvement in body fat percentage. Supplementation had no negative effects on gastrointestinal distress in supplementing females.

**Acknowledgements**

This study was supported by an external grant awarded from the ISSN that was sponsored by MusclePharm.

## P55 The effects of moderate- versus high-load training on body composition, muscle growth, and performance in college aged females

### Jason M Cholewa^1^, Amy Hewins^1^, Samantha Gallo^1^, Ashley Micensky^1^, Christian De Angelis^1^, Christopher Carney^1^, Bill Campbell^2^, Laurin Conlin^2^, Layne Norton^3^, Fabricio Rossi^1,4^

#### ^1^Department of Kinesiology, Coastal Carolina University, Conway, SC 29528, USA; ^2^University of South Florida, Performance & Physique Enhancement Laboratory, Tampa, FL, USA; ^3^Biolayne, LLC. Lutz, FL, USA; ^4^Institute of Bioscience, Department of Physical Education, University Estadual Paulista, Rio Claro, São Paulo, Brazil

##### **Correspondence:** Jason M Cholewa (jcholewa@coastal.edu) – Department of Kinesiology, Coastal Carolina University, Conway, SC 29528, USA

**Background**

A common perception held in mainstream fitness is that heavy resistance training will result in rapid muscular hypertrophy leading to a “bulky” appearance in females. A recent study demonstrated no difference in muscle hypertrophy improvements between 8 weeks of high- and low-load resistance training in trained men; however, this work has not been replicated in women. The purpose of this study was to investigate the effects of moderate- vs. high-load resistance training (RT) on changes in body composition and performance in females.

**Methods**

Thirty young women (20.3±1.5 years, 164±6 cm, 68.7±13.8 kg) without prior structured resistance training experience were recruited for this study. Body composition (BodPod), compartmental water (Bioelectrical Impedance), 7-site skinfold, and arm and thigh cross sectional area were assessed pre- and post-training. Performance testing consisted of vertical jump, 6 kg chest pass peak velocity, squat 1RM and military press 1RM. Following 2 weeks of familiarization training, subjects were matched for body composition and relative squat strength, and randomly assigned to either a high- (n=12; 4 sets of 5-6 repetitions) or moderate-load (n=11; 2 sets of 10-12 repetitions) group. Training was divided into two lower and one upper body training sessions per week and was performed on non-consecutive days for 8 weeks. Each training session consisted of 6-7 exercises performed to momentary muscular failure. Subjects were instructed not to change their dietary habits during the study. Statistical analyses were performed utilizing separate two-way repeated measures ANOVA for each criterion variable with an alpha level p ≤ 0.05.

**Results**

There were no significant group x time interactions for any variable assessed. There were no significant differences in pre- or post-training for sum of skinfolds or fat mass. Main effects for time (pre vs. post) include: a trend (p=.051) for an increase in arm CSA (95.9±17.4 vs. 99.1±13.9 cm^2^). Thigh CSA significantly (p<.001) increased (196.6±26.2 vs. 203.2±22.6 cm^2^). Body mass significantly (p=.029) increased (69.7±13.8 vs. 69.5 ±12.9 kg). Percent body fat significantly (p=.017) decreased (31.2±7.7 vs. 30.1±7.4). Lean mass significantly (p<.001) increased (46.5±5.9 vs. 48.0±6.2 kg). Total body water (TBW) significantly (p=.004) decreased (32.6±4.4 vs. 32.0±3.9 kg). TBW percent of fat free mass significantly (p<.001) decreased (69.2±4.2 vs. 67.6±3.8). Water free fat free mass significantly (p<.001) increased (14.1±1.4 vs. 15.4±1.6 kg). Vertical jump significantly (p=.003) increased (40.8±6.0 vs. 43.7±7.3 cm). Chest pass velocity significantly (p=.015) increased (3.98±0.48 vs. 4.19±0.45 m/s). Squat 1RM significantly (p<.001) increased (59.6±20.7 vs. 81.1±16.5 kg). Military press 1RM significantly (p=.001) increased (27.9±5.7 vs. 30.9± 4.2 kg). Effect size (Cohen’s *d*) differences were found for arm CSA (mod: .50, high: 0.30), TBW (mod: -.51, high: -.84), TBW percent FFM (mod: -.86, high: -1.38) vertical jump (mod: 1.54, high: .45) chest pass velocity (mod: .40, high: .75), and 1RM squat (mod: 1.66, high: 3.77)

**Conclusions**

The results of this study indicate that both moderate- and high-load training are effective at improving muscle growth, body composition, strength and power in college-aged females. Small effect sizes for arm CSA and decreased TBW suggest neither moderate- nor heavy-load training will result in a “bulky” appearance.

## P56 Effect of a multi-ingredient preworkout supplement on cognitive function and perceptions of readiness to perform

### MS Koozehchian^1^, PB Collins^1^, R Sowinski^1^, T Grubic^1^, R Dalton^1^, A O’Connor^1^, SY Shin^1^, Y Peter Jung^1^, BK Sanchez^1^, A Coletta^1^, M Cho^1^, A Reyes^1^, C Rasmussen^1^, CP Earnest^1,3^, PS Murano^2^, M Greenwood^1^, RB Kreider^1^

#### ^1^Exercise & Sport Nutrition Lab, Texas A&M University, College Station, TX, USA; ^2^Institute for Obesity Research & Program Evaluation, Texas A&M University, College Station, TX, USA; ^3^Nutrabolt, Bryan, TX, USA

##### **Correspondence:** MS Koozehchian (majidk@tamu.edu) – Exercise & Sport Nutrition Lab, Texas A&M University, College Station, TX, USA

**Background**

The purpose of this study was to examine the short-term effects of ingesting multi-ingredient preworkout supplements (PWS) and PWS at 1.5 times recommended dose (PWS150) on cognitive function and perceptions of readiness to perform.

**Methods**

We recruited 16 apparently healthy and recreationally active men (21.56 ± 2.11 yr, 20.51 ± 7.64 % fat, 27.28 ± 4.25 kg/m^2^) to participate in a double-blind, crossover, randomized and placebo-controlled manner. Supplements were (1) a dextrose placebo (PLA, 12 g/d); (2) a PWS supplement containing 1.6 g β-alanine, 1.0 g arginine AKG, 1.0 g creatine nitrate, 250 mg ascorbic acid, 150 mg N-acetyl tyrosine, 150 mg caffeine, 5 mg tetramethyluric acid or (3) PWS at ~150 % dosage (PWS150) of the base formula for seven days. Participants were required oral administration of one serving of the supplement mixed in ~8 fl oz. of plain water. On testing days, ingesting followed immediately following 30 minutes resting energy expenditure measurement. During non-testing days of the supplementation week, participants ingested their assigned supplement in two forms: during workout days, they ingested the supplement immediately prior to their workout; during non-workout days, they ingested their assigned supplement around noon. In the supplementation week, participants performed a Stroop-Color cognitive function test (CFT) and rated perceptions of readiness to perform on a visual analogue scale (RTP-VAS) on days one, three, five, and seven. On testing days (days one and seven) participants completed CFT and RTP- VAS approximately 30 minutes following supplementation. On days three and five, they referred to the lab to only perform CFT and RTP-VAS. Participants repeated the experiment after a one week washout period with the alternate supplements in a randomized and counterbalanced manner. Data were analyzed by repeated measure MANOVA or ANOVA and are presented as means ± SD and 95 % confidence interval from baseline.

**Results**

The CFT results indicate a significant interaction between groups for the Word test (p = 0.04). In addition, there was a Time effect between groups for the Word test (p = 0.001), Color test (p = 0.002), and Word-Color test (p = 0.001). A significant change from baseline was seen in cognitive function as determined by the Stroop Word-Color Test (p < 0.05). In the regard, we observed a significant improvement in Word count in supplement groups at day three: PWS (6.56 counts, 95 % CI, 1.99, 11.13), PWS150 (4.75 counts, 95 % CI, 0.17, 9.32), but not the PLA (3.06 counts, 95 % CI, -1.50, 7.63); at day five for supplement groups: PWS (6.56 counts, 95 % CI, 1.99, 11.13), PWS150 (4.75 counts, 95 % CI, 0.17, 9.32), but not the PLA (3.06 counts, 95 % CI, -1.50, 7.63); and at day seven for supplement groups: PWS (6.12 counts, 95 % CI, 0.23, 12.01), PWS150 (13.06 counts, 95 % CI, 7.17, 18.94), but not the PLA (1.81 counts, 95 % CI, -4.07, 7.69); For Color recognition, significant improvements were seen in PWS150 and PLA groups at day seven: PWS150 (8.12 counts, 95 % CI, 3.89, 12.35), PLA (4.25 counts, 95 % CI, 0.02, 8.47), but not the PWS group (1.93 counts, 95 % CI, -2.29, 6.16). For the Word-Color assessment, the improvement was seen only in PWS150 at day five: PWS150 (4.87 counts, 95 % CI, 0.22, 9.52), but not the PWS (1.81 counts, 95 % CI, -2.83, 6.46), and PLA groups (1.68 counts, 95 % CI, -2.96, 6.33); and at day seven for PWS150 and PLA groups: PWS150 (4.87 counts, 95 % CI, 0.22, 9.52), PLA (1.81 counts, 95 % CI, -4.07, 7.69); but not the PWS (3.06 counts, 95 % CI, -2.46, 8.58). No significant interactions or changes from baseline were observed between groups regarding perceived readiness to perform as determined by the VAS (p > 0.05).

**Conclusion**

These data indicate that ingesting a multi-ingredient PWS results in a statistically significant improvement in measured indices of cognitive function test compared to placebo. Furthermore, a significant dose-dependent difference was also observed, as PWS150 showed a higher impact on cognitive performance compared to PWS.

